# Analysis of the Genome and Transcriptome of *Cryptococcus neoformans* var. *grubii* Reveals Complex RNA Expression and Microevolution Leading to Virulence Attenuation

**DOI:** 10.1371/journal.pgen.1004261

**Published:** 2014-04-17

**Authors:** Guilhem Janbon, Kate L. Ormerod, Damien Paulet, Edmond J. Byrnes, Vikas Yadav, Gautam Chatterjee, Nandita Mullapudi, Chung-Chau Hon, R. Blake Billmyre, François Brunel, Yong-Sun Bahn, Weidong Chen, Yuan Chen, Eve W. L. Chow, Jean-Yves Coppée, Anna Floyd-Averette, Claude Gaillardin, Kimberly J. Gerik, Jonathan Goldberg, Sara Gonzalez-Hilarion, Sharvari Gujja, Joyce L. Hamlin, Yen-Ping Hsueh, Giuseppe Ianiri, Steven Jones, Chinnappa D. Kodira, Lukasz Kozubowski, Woei Lam, Marco Marra, Larry D. Mesner, Piotr A. Mieczkowski, Frédérique Moyrand, Kirsten Nielsen, Caroline Proux, Tristan Rossignol, Jacqueline E. Schein, Sheng Sun, Carolin Wollschlaeger, Ian A. Wood, Qiandong Zeng, Cécile Neuvéglise, Carol S. Newlon, John R. Perfect, Jennifer K. Lodge, Alexander Idnurm, Jason E. Stajich, James W. Kronstad, Kaustuv Sanyal, Joseph Heitman, James A. Fraser, Christina A. Cuomo, Fred S. Dietrich

**Affiliations:** 1 Institut Pasteur, Unité Biologie et Pathogénicité Fongiques, Département Génomes et Génétique, Paris, France; 2 INRA, USC2019, Paris, France; 3 University of Queensland, School of Chemistry and Molecular Biosciences, Brisbane, Queensland, Australia; 4 Institut Pasteur, Plate-forme Transcriptome et Epigénome, Département Génomes et Génétique, Paris, France; 5 Duke University Medical Center, Department of Molecular Genetics and Microbiology, Durham, North Carolina, United States of America; 6 Jawaharlal Nehru Centre for Advanced Scientific Research, Molecular Biology and Genetics Unit, Bangalore, India; 7 Genotypic Technology Private Limited, Bangalore, India; 8 Institut Pasteur, Unité Biologie Cellulaire du Parasitisme, Département Biologie Cellulaire et Infection, Paris, France; 9 INRA, UMR 1319 Micalis, Jouy-en-Josas, France; 10 Yonsei University, Center for Fungal Pathogenesis, Department of Biotechnology, Seoul, Republic of Korea; 11 Rutgers New Jersey Medical School, Department of Microbiology and Molecular Genetics, Newark, New Jersey, United States of America; 12 Washington University School of Medicine, Department of Molecular Microbiology, St. Louis, Missouri, United States of America; 13 Broad Institute of MIT and Harvard, Cambridge, Massachusetts, United States of America; 14 University of Virginia, Department of Biochemistry and Molecular Genetics, Charlottesville, Virginia, United States of America; 15 California Institute of Technology, Division of Biology, Pasadena, California, United States of America; 16 University of Missouri-Kansas City, School of Biological Sciences, Division of Cell Biology and Biophysics, Kansas City, Missouri, United States of America; 17 Canada's Michael Smith Genome Sciences Centre, BC Cancer Agency, Vancouver, British Columbia, Canada; 18 Clemson University, Department of Genetics and Biochemistry, Clemson, South Carolina, United States of America; 19 University of North Carolina, Department of Genetics, Chapel Hill, North Carolina, United States of America; 20 University of Minnesota, Microbiology Department, Minneapolis, Minnesota, United States of America; 21 University of Queensland, School of Mathematics and Physics, Brisbane, Queensland, Australia; 22 Duke University Medical Center, Duke Department of Medicine and Molecular Genetics and Microbiology, Durham, North Carolina, United States of America; 23 University of California, Department of Plant Pathology & Microbiology, Riverside, California, United States of America; 24 Michael Smith Laboratories, Department of Microbiology and Immunology, Vancouver, British Columbia, Canada; Oregon State University, United States of America

## Abstract

*Cryptococcus neoformans* is a pathogenic basidiomycetous yeast responsible for more than 600,000 deaths each year. It occurs as two serotypes (A and D) representing two varieties (i.e. *grubii* and *neoformans*, respectively). Here, we sequenced the genome and performed an RNA-Seq-based analysis of the *C. neoformans* var. *grubii* transcriptome structure. We determined the chromosomal locations, analyzed the sequence/structural features of the centromeres, and identified origins of replication. The genome was annotated based on automated and manual curation. More than 40,000 introns populating more than 99% of the expressed genes were identified. Although most of these introns are located in the coding DNA sequences (CDS), over 2,000 introns in the untranslated regions (UTRs) were also identified. Poly(A)-containing reads were employed to locate the polyadenylation sites of more than 80% of the genes. Examination of the sequences around these sites revealed a new poly(A)-site-associated motif (AUGHAH). In addition, 1,197 miscRNAs were identified. These miscRNAs can be spliced and/or polyadenylated, but do not appear to have obvious coding capacities. Finally, this genome sequence enabled a comparative analysis of strain H99 variants obtained after laboratory passage. The spectrum of mutations identified provides insights into the genetics underlying the micro-evolution of a laboratory strain, and identifies mutations involved in stress responses, mating efficiency, and virulence.

## Author Summary

*Cryptococcus neoformans* var. *grubii* is a major human pathogen responsible for deadly meningoencephalitis in immunocompromised patients. Here, we report the sequencing and annotation of its genome. Evidence for extensive intron splicing, antisense transcription, non-coding RNAs, and alternative polyadenylation indicates the potential for highly intricate regulation of gene expression in this opportunistic pathogen. In addition, detailed molecular, genetic, and genomic studies were performed to characterize structural features of the genome, including centromeres and origins of replication. Finally, the phenotypic and genome re-sequencing analysis of a collection of isolates of the reference H99 strain resulting from laboratory passage revealed that microevolutionary processes during *in vitro* culturing of pathogenic fungi can impact virulence.

## Introduction

Fungal pathogens pose a major threat to human health because of their proclivity to infect immunocompromised individuals, particularly those afflicted by HIV/AIDS or who have received organ transplants and immunosuppressive therapy [1]. Among these pathogens, the basidiomycete yeast *Cryptococcus neoformans* is globally distributed and causes pneumonia and meningoencephalitis in an estimated 1 million people annually, leading to ∼620,000 deaths per year [2]. The burden of cryptococcal disease is remarkably high in developing nations (i.e., in India, Africa, and southeast Asia), where it accounts for approximately one-third of all deaths in HIV/AIDS patients, surpassing mortality rates attributable to tuberculosis in some areas [2]. *C. neoformans* comprises two varieties (var.), *grubii* (serotype A) and *neoformans* (serotype D); a former third variety (*gattii*, serotype B) is now recognized as the separate species *Cryptococcus gattii* [3].

The *Cryptococcus* research community initially mapped out genome sequencing projects for the commonly studied strains of *C. neoformans* representing the different varieties [4]. This strategy yielded a comparative analysis of the genomes of two var. *neoformans* isolates and employed a large set of expressed sequence tags to establish robust gene annotations [5]. Importantly, this study revealed that *C. neoformans* genes are intron-rich with the frequent occurrence of alternative splicing and antisense transcription. Subsequently, the community responded to a remarkable outbreak of *C. gattii* disease among immunocompetent people in western North America by sequencing two genomes, one representing the major outbreak genotype and the other representing the more common global type [6]. This analysis provided evidence for further speciation within the *C. gattii* complex of genotypes as well as a view of extensive genome variation within the complex and between *C. gattii* and *C. neoformans* genomes.

**Figure 1:**
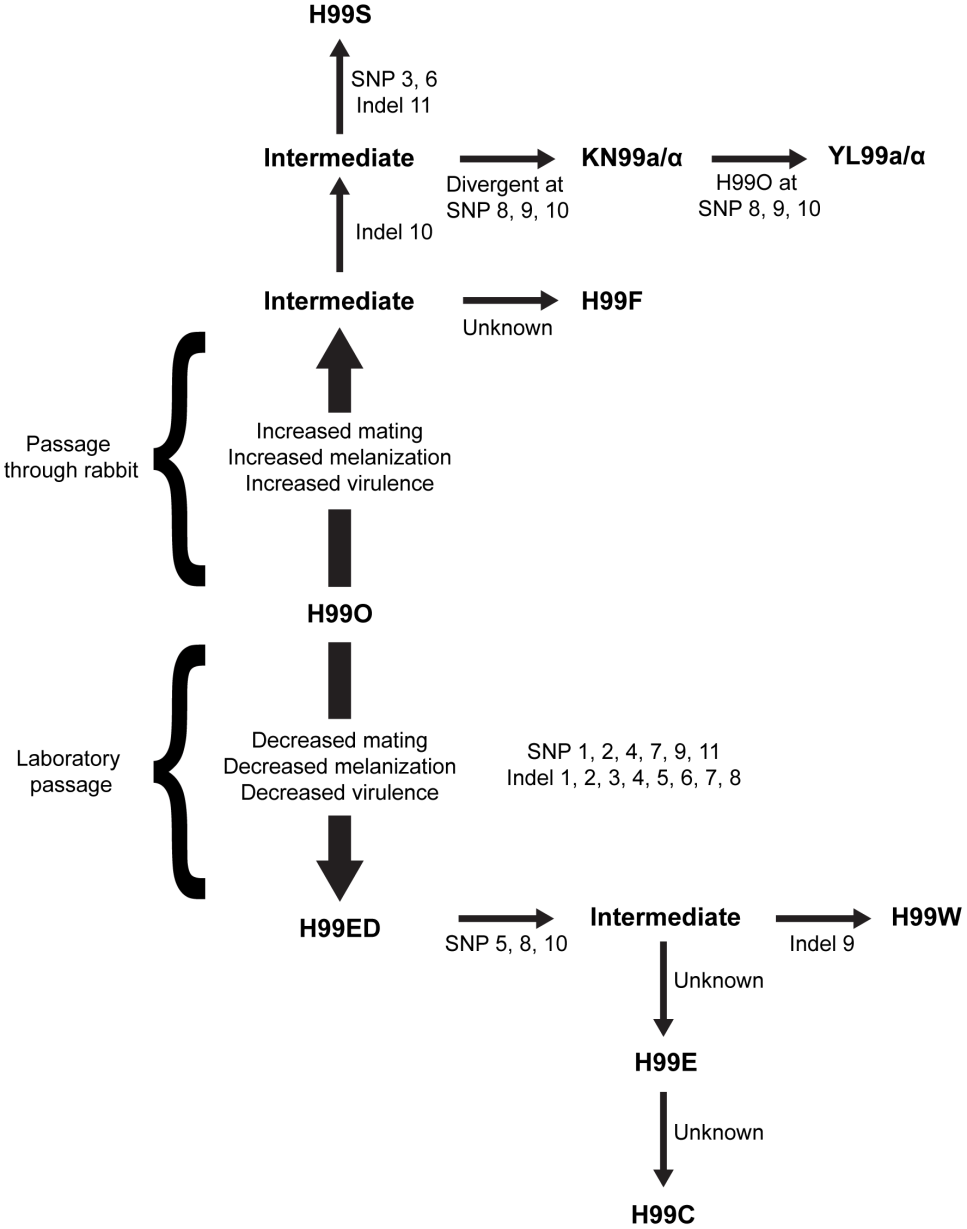
Origins of the independent lineages of H99. Since the initial publication, the isolate has lost virulence following laboratory passage (possibly multiple independent times) and was subsequently passaged through the rabbit model of infection to increase virulence and distributed to many labs. All variants were derived from the original sequenced H99 isolate (H99O), and the major strain variants of this study have been termed H99W, H99E, and H99S. The origins of this strain series are as follows. During laboratory passage by repeated growth on YPD rich medium, the H99W/H99ED isolates arose from the H99O original stock (frozen in 1994). H99W and H99ED are distinguished from the parental strain by reduced melanin production, impaired mating, and attenuated virulence. This isolate or a closely related derivate of H99O was sent to the Lodge laboratory (Washington University, St Louis, USA) (H99E), and was subsequently distributed to the Madhani laboratory (University of California, San Francisco, USA) (H99CMO18, hereafter named H99C). Thus, isolates H99W and H99ED (Duke University), H99E (Washington University), and H99C (UCSF) are all closely related to one another. Additionally, John Perfect (Duke University Medical Center, USA) derived the H99S isolate via passage of a mixed H99 frozen stock through the well-validated rabbit model of central nervous system (CNS) infection. The pedigree was constructed based on SNPs and indels identified from sequence analysis. Specific mutations separating independent strains are annotated.

Here, we report the latest community effort to enhance genomic resources for *C. neoformans* by analyzing the genomes and transcriptomes of lineage H99, derived from the primary strain (H99O) of *C. neoformans* var. *grubii* (Figure 1). Importantly, strain H99 has been used for virtually all genetic, molecular, and virulence studies conducted with *C. neoformans* var. *grubii* and for the majority of virulence studies in recent years with *C. neoformans* in general. This fact is relevant to human cryptococcosis because var. *grubii* strains are generally more virulent than var. *neoformans* strains and, globally, strains of var. *grubii* cause the vast majority of disease including >99% of infections in AIDS patients and >95% of those overall. A working draft of the H99 genome has been available for several years, and it has been used extensively by the community for the examination of fungal pathogenesis and aspects of unisexual and opposite-sex mating dynamics [7]–[9].

The current analysis employed extensive RNA-Seq experiments to significantly improve the annotation and to provide an exceptionally robust analysis of RNA expression in the context of intron splicing, strand-specific transcription, and non-coding RNAs. This analysis revealed a high complexity of the transcriptome structure. In addition, detailed studies were performed to characterize structural features of the genome, including centromeres and origins of replication.

Finally, resequencing and genetic analyses were employed to explain a long-standing phenomenon in pathogen biology: the loss of virulence and other attributes such as fecundity upon laboratory passage. Taken together, these studies provide a detailed characterization of the genome of an essential reference strain to support further efforts in understanding cryptococcal pathogenesis.

## Results/Discussion

### Genome sequencing and chromosome assembly

**Figure 2:**
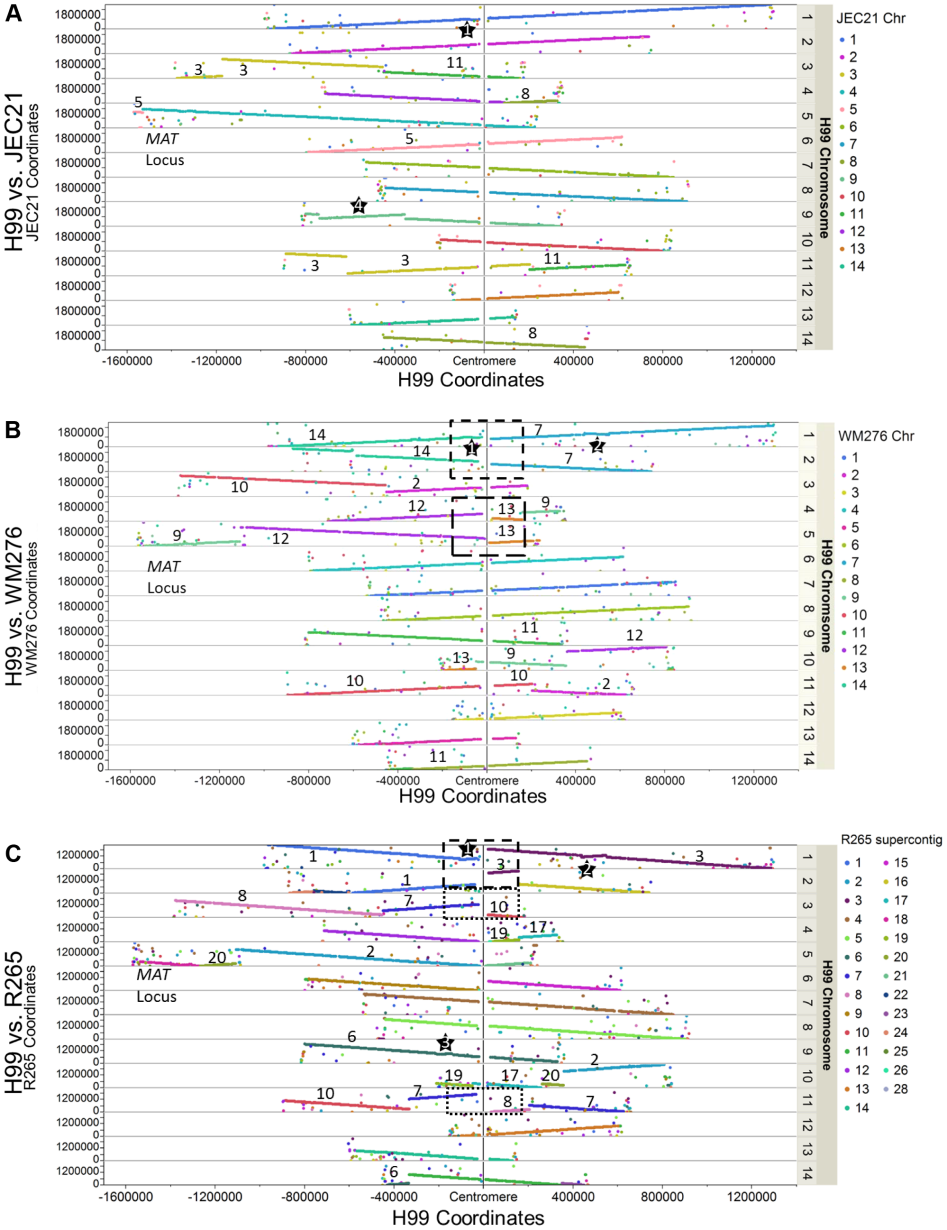
Genome comparisons between H99 and other *Cryptococcus neoformans* (JEC21 - A) and *Cryptococcus gattii* (WM276 - B and R265- C) strains. Each dot represents the best tBLASTn return in the target genome when a protein sequence of H99 was used as query. The X axis shows the coordinates of the H99 chromosomes anchored on the centromeres at the middle. The Y axis shows the coordinates of the tBLASTn hits on their respective supercontigs/chromosomes in the target genomes. When the two chromosomes under comparison are in synteny, the BLAST hits of that H99 chromosome form a straight line composed by dots of same color (e.g. H99 chromosome 1 in Figure 2A). If there are chromosomal translocations, the BLAST hits of the H99 chromosome are composed of dots with different colors. Additionally, large-scale inversions (>60 kb in size) are highlighted by stars and boxes showing the potential translocations mediated by centromeres (see Results/Discussion). Numbers indicate the chromosomes/supercontigs in the target genome that have undergone translocations relative to the H99 genome.

The genome of the *C. neoformans* var. *grubii* H99 strain was sequenced using Sanger technology and assembled into 14 finished scaffolds. Each sequence scaffold corresponds to a single chromosome, with a total length of 18.9 Mb, a size very similar to the ones previously published for *C. neoformans* var. *neoformans* and *C. gattii* [5], [6]. We conducted whole genome comparisons between H99 and three other *C. neoformans* and *C. gattii* genomes (JEC21 – serotype D, WM276 – VGI, and R265 – VGII; Figure 2). The comparison between H99 and JEC21 showed that the two genomes are in overall synteny with a few chromosomal rearrangements. Specifically, we identified three translocations that involve H99 chromosomes 3, 4, 5, and 11 (Figure 2A). Additionally, our analysis identified a 400-kb region on H99 chromosome 9 that is inverted between H99 and JEC21, demarcated with star 4. We also identified a second large inversion on H99 chromosome 1 with respect to all three genomes, suggesting via parsimony that there has been a single inversion in H99 relative to the shared common ancestor (star 1). It should be noted that the chromosomal rearrangements identified between H99 and JEC21 genomes herein are consistent with those that have been reported previously [10], [11].

Comparisons between H99 and the two *C. gattii* genomes revealed more extensive chromosomal rearrangements. Six translocations involving nine H99 chromosomes were apparent when comparing H99 and WM276, while there are at least six translocations involving nine H99 chromosomes when H99 and R265 are compared with each other (Figures 2B and 2C). We identified one large chromosomal inversion on H99 chromosome 1 when it is compared to WM276, which is also apparent by comparison to R265 (star 2). This inversion is shared between H99 and JEC21 and distinguishes the *C. neoformans* (A and D) and *C. gattii* lineages. There is an additional inversion when H99 is compared to R265, which is located on H99 chromosomes 9 (star 3) (Figures 2A–2C). These chromosomal rearrangements identified between H99 and the *C. gattii* genomes are in overall agreement with those reported previously between serotype D *C. neoformans* and *C. gattii*, suggesting that these rearrangements may be ancestral to the *C. gattii* split from *C. neoformans* [6].

### Gene prediction and conservation

An initial set of 6,967 protein-coding genes was predicted by combining the results of different gene prediction programs (see Material and Methods). To validate and refine the predicted gene structures, deep-coverage RNA-sequence was generated from different conditions using independent methods. For strand-specific sequencing, poly(A) RNA was purified from cells grown under three different conditions sampled in duplicate: YPD, starvation medium (low glucose and nitrogen medium), and pigeon guano broth (PG) (see Material and Methods). For non-strand-specific sequencing, poly(A) RNA was purified from cells growing under six different conditions in duplicate: YPD exponential phase 30°C; YPD exponential phase 37°C, YPD stationary phase 30°C, YPD exponential phase with 0.01% SDS, YPD exponential phase with fluconazole (10 mg/mL) and YP galactose stationary phase. Trimmed reads were aligned to the H99 genome using Bowtie and TopHat [12], [13]. After elimination of the reads specific to the rRNA loci, a total of 795×10^6^ reads and 244×10^6^ strand-specific reads covered 92% of the genome with at least two reads. Read alignments were compared to the initial gene set of 6,967 predicted genes.

Incorporation of the RNA-Seq data improved gene structure accuracy by validating and modifying predicted intron-exon boundaries. We found at least 30 reads spanning predicted exon/intron boundaries for 87% of the introns present in the annotation (n = 32,345), confirming the *in silico* predicted gene structures. In contrast, 7% of the annotated introns had no spanning reads despite being within an expressed gene, suggesting a potential incorrect annotation. More importantly, we identified 4,724 new introns, resulting in the alteration in the sequence of nearly one-third of the coding sequences (n = 2,705). We identified relatively few new coding genes (n = 53) and deleted about the same number (n = 58), mainly through gene fusion (Table S1). Overall, 6,962 protein-coding genes were predicted, which occupy 85% of the total genome. The remaining 15% are centromeres and intergenic regions. The poly(A) site positions (see below) and strand-specific RNA-Seq data were used to identify precisely the start and stop sites of the transcripts for 92% of these genes.

In order to check the validity of these changes, the sequences of the old protein set and the sequences of the protein set based on the updated annotation were compared with the *S. cerevisiae* set of protein sequences. This comparison was carried out for the 1766 proteins where the sequence was changed, excluding proteins that were added or deleted from the gene set as well as those where the new annotation was for a completely different transcript. These putative proteins were compared to a modified set of the *S. cerevisiae* proteins, where highly similar duplicate genes were removed. These highly similar *S. cerevisiae* proteins were removed to reduce, but not completely eliminate, the possibility of aligning the two *C. neoformans* proteins to different *S. cerevisiae* orthologs. Proteins were aligned using BLAST. In cases where the new annotation version of the *C. neoformans* gene was aligned to an *S. cerevisiae* protein with more than 30% identity, the percent identity between the *S. cerevisiae* protein and the new and old *C. neoformans* annotations were compared. This percent identity cutoff was determined empirically to eliminate low similarity spurious alignments. A total of 848 *C. neoformans* protein pairs met these criteria (Table S2). Of these 848 protein pairs, 575 proteins from the new annotation showed a higher BLAST bit score in comparison with the putative *S. cerevisiae* homolog, 218 showed no change in BLAST bit score, and 55 showed a lower BLAST bit score. For the 55 cases with a lower BLAST bit score in the new annotation, the change in bit score was very small (less than 2) in 52 cases, the majority of which appeared to be spurious changes in calculations of the bit score resulting from differences in the length of the proteins. For the remaining 3 cases, the new version of the H99 protein set has less similarity to the *S. cerevisiae* protein set, although the changes in BLAST scores remain minor (Table S2).

Comparison of the predicted proteins of H99 to those of the two other *Cryptococcus* lineages and other basidiomycetes identified unique properties of the *Cryptococcus* genomes. We compared proteins from H99 to those of the *C. neoformans* var. *neoformans* JEC21 genome and the *C. gattii* WM276 genome (Figure S1A). A core set of 5,569 orthologs is shared among all three species, with the number of paralogs totaling between 5,749 to 5,793 proteins in each genome. Single-copy orthologs share an average of 93% identity between the two *C. neoformans* genomes and 89% identity between either of these two genomes and that of *C. gattii* (Figure S1B). The H99 genome contains the largest set of unique proteins (n = 573); however, the differences in annotation methods between these three genomes and in particular the use of RNA-Seq for H99 may account for such differences in gene counts.

Comparing the three *Cryptococcus* genomes to four diverse basidiomycetes identified protein families amplified in the *Cryptococcus* lineage. The comparison included two other agaricomycetes (*Coprinopsis cinereus* and *Phanerochaete chrysosporium*) and two ustilaginomycetes (*Ustilago maydis* and *Malassezia globosa*). Of these species, only *M. globosa* is human-associated; *Malassezia* species are commonly found on skin where they are the most common cause of dandruff. Compared to these four basidiomycetes, the three *Cryptococcus* genomes are most highly enriched for transporter families, both Major Facilitator Superfamily (MFS) and sugar transporters (Table S3). In addition, the two *C. neoformans* species (H99 and JEC21) contain larger numbers of transporters than *C. gattii*; for example, the most common MFS family is found in 174, 173, and 149 copies in H99, JEC21, and WM276, respectively. MFS transporters are the largest class of transporters found in fungal genomes; MFS subfamilies transport small molecules, including drugs, metabolites, sugars, and other small molecules [14]. Other notable expansions in the *Cryptococcus* species include fungal-specific transcription factor domains, glucose-fructose oxidoreductases, and phytanoyl-CoA dioxygenases (Table S3). Overall these expansions suggest an increased capacity for transport, a rewiring of transcriptional circuits, and metabolic differences compared to other basidiomycetes.

**Figure 3:**
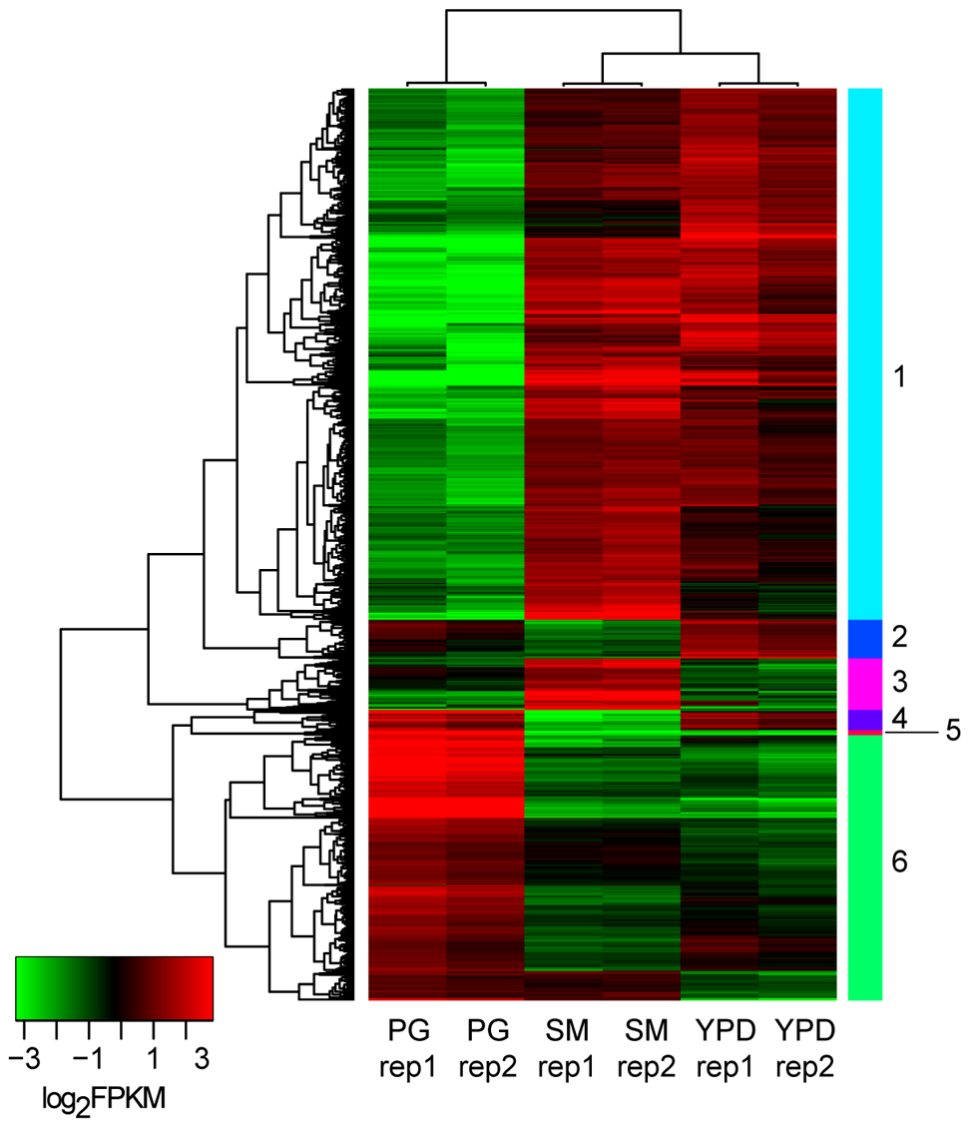
Differentially expressed gene clusters. Genes differentially expressed between the three conditions (PG, pigeon guano; SM, starvation medium; YPD, rich media) were identified from strand-specific RNA-Seq using EdgeR with two biological replicates per condition (rep1, rep2). Expression profiles are ordered based on hierarchical clustering tree; 6 clusters were defined using the kmeans algorithm (Material and Methods).

We identified differentially expressed genes using the strand-specific RNA-Seq data to highlight the major expression shifts between these culture conditions. Reads from two biological replicates from each of the three conditions (YPD, starvation medium, and pigeon guano broth) were mapped to transcripts to quantify their abundance (see Material and Methods). Normalized expression levels (FPKM) for the most highly differentially expressed genes (corrected p-value <0.001; log_2_ fold-change >2) were clustered to identify groups of co-regulated genes. Among these three conditions, rich and limited media produced the most similar expression profiles, while many genes were differentially regulated between both these conditions and pigeon guano (Figure 3). Two clusters of genes (5 and 6) were more highly expressed in pigeon guano as compared to rich and limited media. These clusters of genes were found to be enriched for transporters, transcription factors, and genes involved in lipid metabolism (Table 1). We did not detect significant functional enrichment in the other four clusters. The high expression of transporters and transcription factors under certain growth conditions suggests that these proteins may provide a more diverse repertoire, enabling growth in different ecological niches, including pigeon guano.

**Table 1:** Functional enrichment of PFAM and TIFRfam domains in differentially expressed gene clusters.

Cluster 6; 699 transcripts						
Pfam or TIGRfam domain	Cluster 6	Other genes	p-value	Corr p-value	Relative proportion	Role
PF07690.11 Major Facilitator Superfamily	69	107	0	0	5,39	Transport
TIGR00879 MFS transporter, sugar porter (SP) family	24	24	8,19E-12	5,40E-09	8,37	Transport
PF00083.19 Sugar (and other) transporter	33	58	5,89E-11	2,59E-08	4,76	Transport
PF04082.13 Fungal specific transcription factor domain	26	47	1,05E-08	3,47E-06	4,63	Transcription
PF01408.17 Oxidoreductase family, NAD-binding Rossmann fold	12	6	1,99E-08	5,25E-06	16,73	Redox reactions
PF00172.13 Fungal Zn(2)-Cys(6) binuclear cluster domain	28	62	8,37E-08	1,84E-05	3,78	Transcription
PF13561.1 Enoyl-(Acyl carrier protein) reductase	14	18	1,62E-06	3,05E-04	6,51	Lipid metabolism
PF00106.20 short chain dehydrogenase	16	28	5,51E-06	9,09E-04	4,78	Lipid metabolism
PF00441.19 Acyl-CoA dehydrogenase, C-terminal domain	6	1	9,20E-06	1,21E-03	50,2	Lipid metabolism
PF02770.14 Acyl-CoA dehydrogenase, middle domain	6	1	9,20E-06	1,21E-03	50,2	Lipid metabolism
PF08028.6 Acyl-CoA dehydrogenase, C-terminal domain	5	0	1,36E-05	1,64E-03	4183060,11	Lipid metabolism
PF00501.23 AMP-binding enzyme	6	3	9,14E-05	7,54E-03	16,73	
PF01266.19 FAD dependent oxidoreductase	10	14	8,67E-05	7,54E-03	5,98	Redox reactions
PF02771.11 Acyl-CoA dehydrogenase, N-terminal domain	5	1	7,47E-05	7,54E-03	41,83	Lipid metabolism
PF02894.12 Oxidoreductase family, C-terminal alpha/beta domain	6	3	9,14E-05	7,54E-03	16,73	Redox reactions
PF08659.5 KR domain	12	21	8,37E-05	7,54E-03	4,78	
PF00701.17 Dihydrodipicolinate synthetase family	4	0	1,29E-04	9,44E-03	3346448,09	
PF07350.7 Protein of unknown function (DUF1479)	4	0	1,29E-04	9,44E-03	3346448,09	Unknown

Cluster 5; 13 transcripts						
Pfam or TIGRfam domain	Cluster 5	Other genes	Fisher p	Corr p	Relative proportion	
PF00083.19 Sugar (and other) transporter	5	85	1,02E-06	7,07E-04	25,3	Transport
PF07690.11 Major Facilitator Superfamily	6	168	1,04E-06	7,07E-04	15,36	Transport
PF12006.3 Protein of unknown function (DUF3500)	2	0	4,93E-06	2,24E-03	86033333,33	Unknown

### RNA-Seq analysis identified a large number of miscRNAs

**Figure 4:**
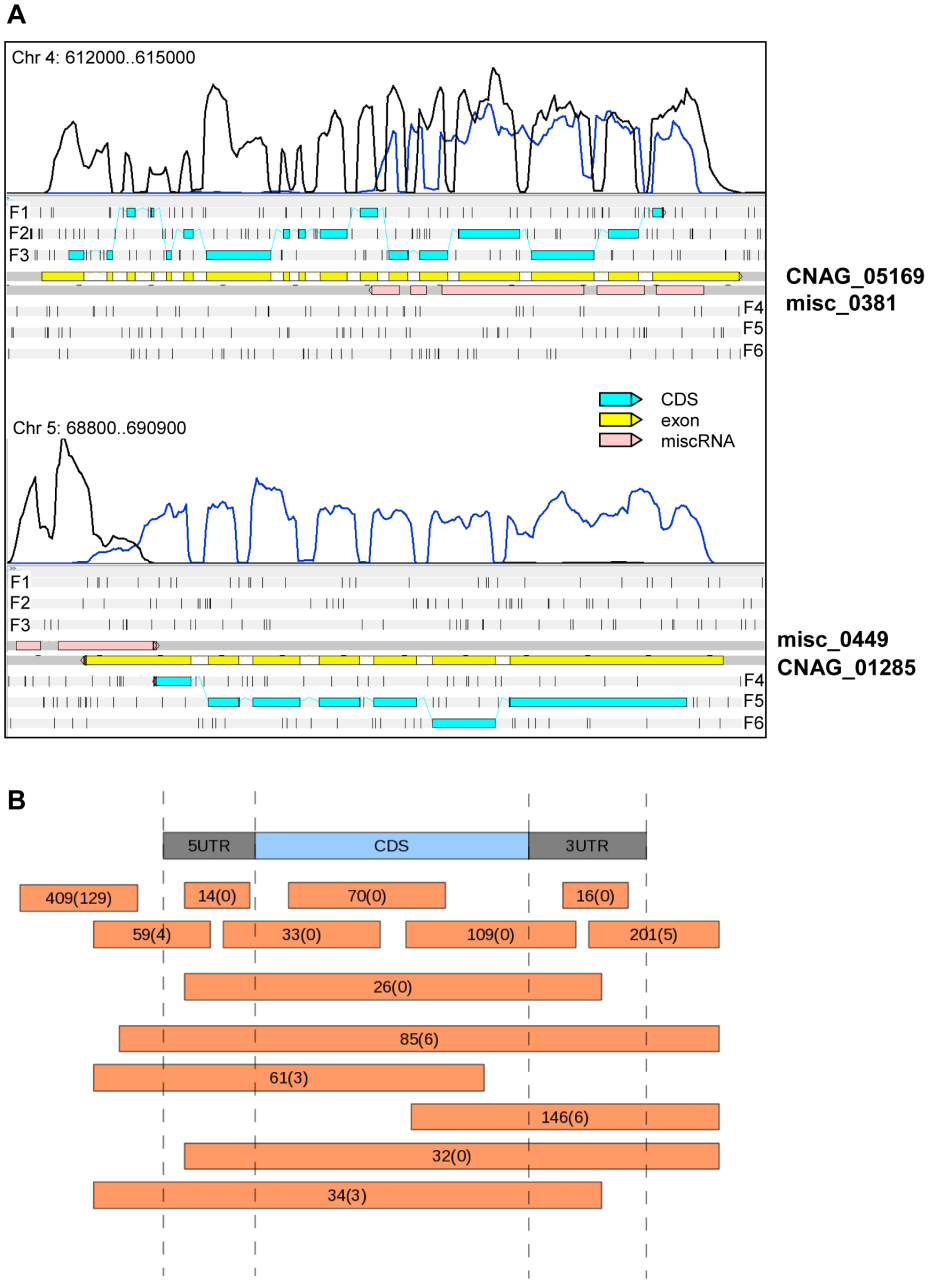
miscRNAs in *C. neoformans* var. *grubii*. **A.** Two examples of a miscRNA as visualized through Artemis. The coverage of the plus stand is represented by the black curve. The coverage of the minus strand is represented by the blue curve. These results were obtained when cells grown in low glucose and nitrogen medium (starvation medium) underwent strand-specific sequencing. F1, F2, and F3 stand for 5′ to 3′ frames 1, 2, and 3, respectively. F4, F5, and F6 stand for 3′ to 5′ frames 1, 2 and 3, respectively. The small black vertical bars indicate the position of the stop codons for each frame. **B.** Schematic representation of the positions of the miscRNAs in the *C. neoformans* var. *grubii* genome as compared to coding sequences. The numbers of miscRNAs at each position is indicated. The number of miscRNAs in the antisense strand of other miscRNAs is indicated between brackets.

In addition to coding genes, intron identification and manual annotation of strand-specific and non-specific RNA-Seq data allowed the identification of 1,197 transcribed regions that were named miscellaneous RNA (miscRNA) (Figure 4). These miscRNAs can be very short (minimum size = 106 nt) or span several kbs (maximum size = 5,555 nt). Several lines of evidence argue that these are present in the cell and are not artifacts resulting from the sequencing or/and alignment process. First, most of the miscRNAs contain spliced introns (n = 765) or/and a poly(A) site (n = 486), suggesting that they are processed in the same way as coding gene mRNAs. In addition, although their coding capacity is unknown, some of these miscRNAs may in fact code for small proteins, as small hypothetical ORFs can be identified in some. Indeed, virulence-associated small proteins have been previously identified in several different plant-pathogenic fungi [15]. Moreover, ribosome profiling has recently revealed the widespread occurrence of functional peptides encoded by small ORFs (smORF) in metazoans [16], [17]. In *C. neoformans* var. *grubii*, the putative proteins encoded by these small ORFs share no sequence homology with any known proteins in other organisms, and the existence of the encoded small proteins in this yeast will require experimental validation. A subset of these miscRNAs could be noncoding RNAs with structural or regulatory roles. The hypothesis of a regulatory role of some miscRNAs is supported by the fact that they are mostly antisense of a coding gene or of another miscRNA (Figure 4), suggesting a potential role in gene expression regulation (see below). One *Cryptococcus* non-coding RNA has been reported as unpublished data in a recent review as critical for the morphologic switch between the yeast and hyphal form [18]. More experiments are clearly needed to characterize the roles of these miscRNAs in *C. neoformans*. Finally, we have considered only the polyadenylated RNA, whereas some studies in *S. cerevisiae* and in mammals suggest the existence of a non-polyadenylated ncRNA population, which would further increase the complexity of the transcriptome structure [19], [20].

### Introns in *C. neoformans*

*C. neoformans* and other basidiomycetes are the most intron-rich fungal species [21], and these introns have been recently shown to be important modulators of gene expression in this yeast [22]. We identified 40,946 introns in the genome, and 99.5% of the expressed genes were found to contain at least one intron. Most of these introns are located within the coding sequences (n = 36,855), but 1,632 and 1,025 introns are located in the 5′-UTRs and 3′-UTRs, respectively. As noted above, the miscRNAs also contain introns, and we found 1,434 introns in miscRNA sequences. The measured intron-density is high (3.35 introns/kb of coding sequence) and similar to what has been reported for some other basidiomycetous fungi based on automatic annotation [23]. Accordingly, exons are small in *C. neoformans* var. *grubii* (median size = 194 nt). Remarkably, some exons are as small as 1 bp. making them difficult to identify through an automatic process (see Material and Methods). A typical *C. neoformans* gene contains 5.7 introns per gene on average, although extreme cases with many more or no introns have been observed. The most intron-rich gene, which encodes Tco4p, one of seven hybrid histidine kinases, contains 42 introns (CNAG_03355) [24]. On the other hand, we identified only 35 genes (Table S4) that are expressed in at least one condition without any intron in their sequences; 10 of these encoded proteins are unique to *C. neoformans* species. Interestingly, one of the 35 encoded proteins (CNAG_02933) shares homology with bacterial quinone oxidoreductases, suggesting a possible horizontal gene transfer from a bacterium into the ancestor of the *C. neoformans*/*C. gattii* species complex.

**Figure 5:**
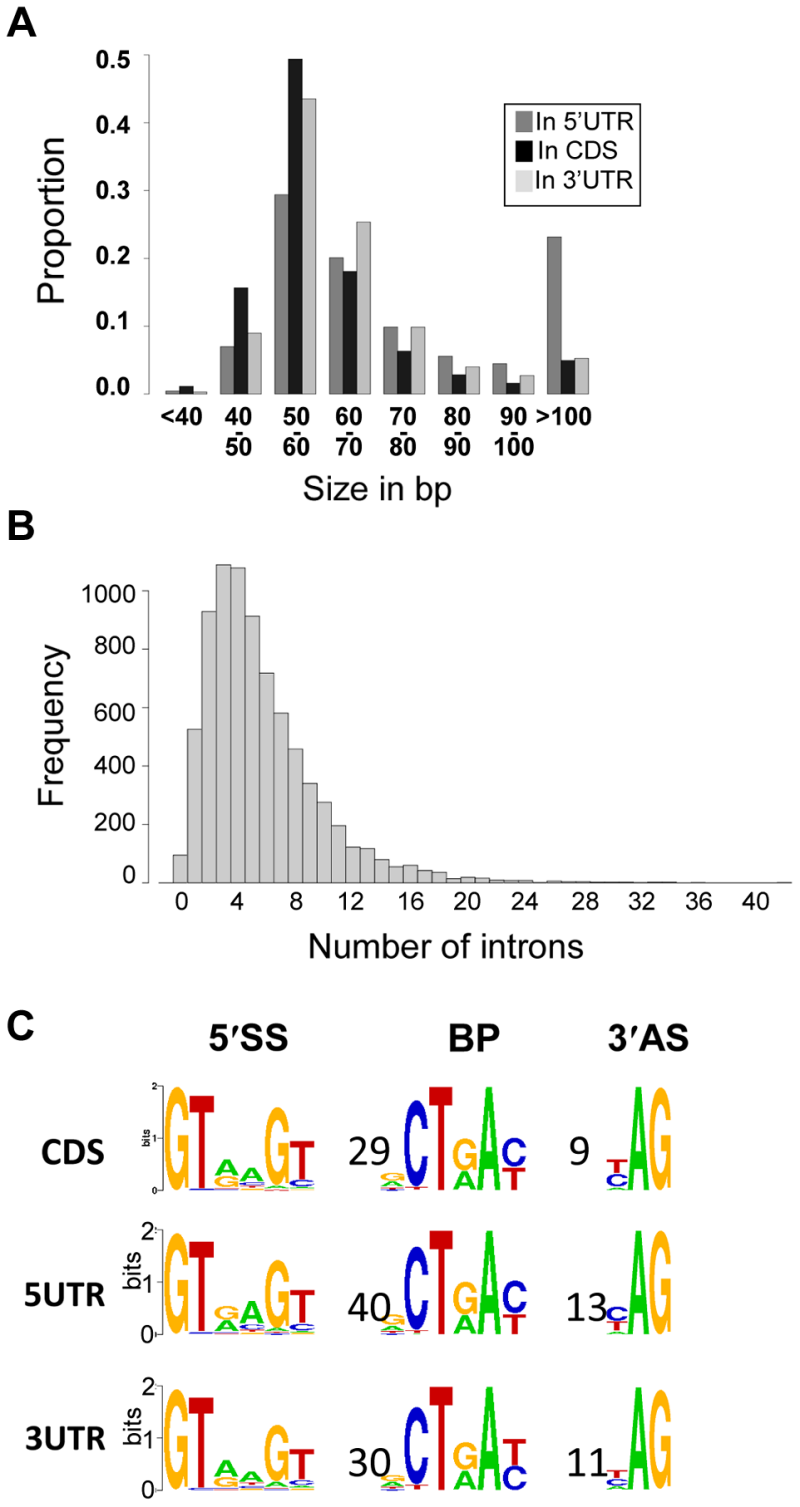
Introns in *C. neoformans* var. *grubii*. **A.** Distribution of the introns according to their sizes. **B.** Distribution of the number of introns per gene. **C.** Motifs associated with introns in *C. neoformans* var. *grubii*. Numbers represent the average distance in bp between the motifs. The overall height of the stack indicates the sequence conservation at that position, while the height of symbols within the stack indicates the relative frequency of the nucleic acid at that position.

Most of the *C. neoformans* var. *grubii* introns are small (median size = 56 nt) whereas some larger ones are present (maximum size = 2,124 nt). Overall, there is very little difference in the characteristics of the introns according to their location within transcripts. Nevertheless, we found that introns within the coding sequences are slightly shorter (median size = 55 nt) than introns within the 5′-UTR (median size = 65 nt) and 3′-UTR (median size = 59 nt). Analysis of the motifs associated with the introns confirmed the splice site consensus sequences previously identified using a smaller set of data [25] (Figure 5), and we found no variation of these motifs based on the intron location.

**Figure 6:**
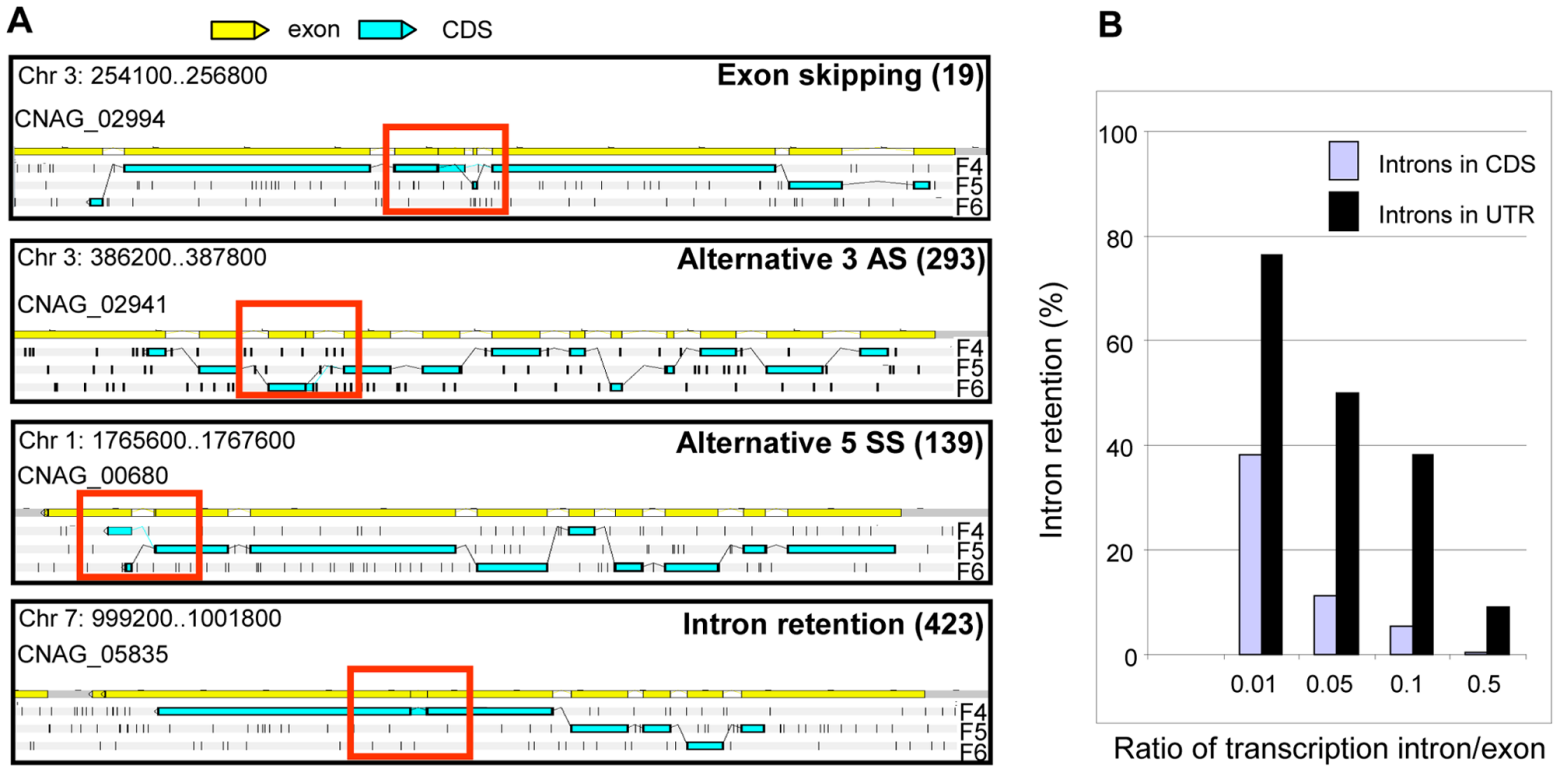
Alternative splicing in *C. neoformans* var. *grubii*. **A.** Examples of alternative splicing. F4, F5, and F6 stand for 3′ to 5′ frames 1, 2 and 3, respectively. The small black vertical bars indicate the position of the stop codons for each frame. The numbers for each type of alternative splicing events annotated in the genome are indicated between brackets. **B.** Evaluation of intron retention level in *C. neoformans* according to the ratio of transcription intron/exon threshold used is represented.

We identified alternatively spliced transcripts based on the RNA-Seq data for 741 genes, a level similar to that previously reported for *C. neoformans* var. *neoformans* [5]. In the 10.6% of genes with more than one mRNA transcript, these isoforms are the consequence of exon skipping, alternative 3′ or 5′ splice site selection, or intron retention (Figure 6). Analysis of PFAM domains revealed that the 741 genes with alternative transcripts are significantly enriched for transporters (MFS and sugar transporter domains) by 2 to 3 fold (Fisher's exact test, q-value <0.05).

Manual examination of intron splicing revealed additional alternative forms shifted by a few base pairs upstream or downstream from the predominant transcript. Most of these alternative forms were not included in the annotation largely because their proportion was often small compared to the major splicing pattern. In addition, the multi-intronic character of *C. neoformans* transcripts renders the number and nucleotide sequences of transcript isoforms difficult to predict in cases where more than one intron displays alternative splicing. Overall, we determined that more than 6% of the introns located within the protein-coding regions displayed an alternative form. This proportion reaches 27% and 19% in the 5′-UTRs and 3′-UTRs, respectively. In the CDS, we found that 49% of these alternative splicing events maintained the frame of the coding sequence, whereas 51% caused frame shifts or introduced premature termination codons. Finally, we noticed that it was very common for intron retention to introduce a stop or a frame-shift in a protein. As shown in Figure 6, the ratio of transcription of the intron compared to one of the surrounding exons is above 0.05 for more than 11.4% of the introns. Strikingly, if one considers a ratio of 0.01, this percentage of retained introns reaches 38% in CDS and 76% in UTRs (Figure 6). Further studies are needed to determine whether this type of alternative splicing results from stochastic errors by the splicing machinery or if this is biologically regulated.

### Identification of poly(A) sites

**Figure 7:**
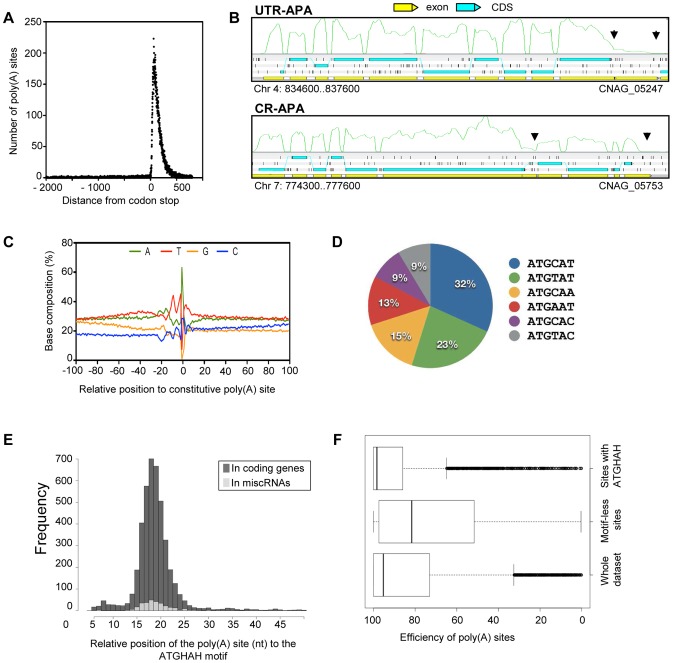
Polyadenylation sites in *C. neoformans* var. *grubii*. **A.** Poly(A) reads are enriched within 500 nt from the stop codon of the gene models. **B.** Examples of CR-APA and UTR-APA as visualized through Artemis. The black arrows indicate the position of the alternative poly(A) sites. The green curves indicate the plus strand coverage. The small black vertical bars indicate the position of the stop codons for each frame. **C.** Sequence composition around the poly(A) cleavage sites. **D.** Proportion of the variants of the AUGHAH motif. **E.** Relative position of the poly(A) site (nt) to the AUGHAH motif in coding genes and miscRNAs. **F.** Efficiency of the poly(A) sites to induce transcription termination according to the presence or absence of an AUGHAH motif.

We screened all of the unaligned RNA-Seq reads derived from the different strand non-specific sequencing experiments for those that contain poly(A) tails. These were used to identify poly(A) sites in *C. neoformans* var. *grubii* (see Material and Methods) [26]. To validate the identified poly(A) sites, we assessed the location of poly(A) sites relative to stop codons for all gene models. As expected for valid sites, most poly(A) sites fall within 500 nt downstream of the codon stop (Figure 7A). These results suggest that most of the identified poly(A) sites are authentic. In total, we defined a poly(A) site for about 82% (n = 5,634) of the gene models. The median distance from the poly(A) site to the corresponding stop codon is 106 nt.

Examples of micro-heterogeneity in poly(A) cleavage sites in metazoans are well documented [27]. Here, we quantified micro-heterogeneity observed in *C. neoformans*. First, we grouped the poly(A) sites into clusters by allowing certain cut offs of the maximum distance between sites within a cluster. About 80% of the sites could be grouped as clusters when a maximum cut off of 15 nt between sites was allowed; further increasing the cut off did not significantly increase the percentage of clustered sites (Figure S2). This result suggests that most of the sites are in proximity to other sites within a range of 15 nt; we therefore chose 15 nt as the cut off to define poly(A) clusters. In fact, 95% of poly(A) clusters are less than 30 nt. Each poly(A) cluster was represented by a constitutive site (i.e. the “peak” with the most number of reads), and the rest were termed alternative sites.

We defined alternative polyadenylation (APA) as multiple poly(A) clusters on the same mRNA. In this case, we considered only clusters that are at least 50 nt away from each other. Indeed, ∼99% of poly(A) clusters are less than 50 nt in size (Figure S2). Although 95% of the poly(A) sites are within 425 nt of the stop codon, automatic annotation and manual curation of 3′-UTRs using strand-specific data identified poly(A) clusters very distant from the stop codon (maximum = 3,018 nt). APA was observed in the 3′-UTR of ∼37% (n = 2069) of the genes for which at least one poly(A) site was identified in the 3′-UTR region. This type of APA (previously named UTR-APA [28]) alters the size of the 3′-UTR without affecting the sequence of the encoded protein (Figure 7B). Notably, 165 of these APAs were located in introns, suggesting a competition between splicing and polyadenylation of the mRNA in *C. neoformans*. We also searched for “internal poly(A) clusters”, i.e. poly(A) clusters between the start and stop codons of the genes. In total, 789 genes were found to have internal poly(A) clusters. In this case, APA should result in production of an alternative protein (Figure 7B). This type of APA has been previously named CR-APA [28]. As expected, these APA were found in introns for 194 genes. In addition, and as previously reported in plants, we also found APA in exonic sequences [29]. APA has been recently recognized as a global phenomenon acting as a major player in regulating gene expression in different eukaryotic organisms [30], [31]. Indeed, the length of 3′-UTR is known to regulate mRNA stability, mRNA export to the cytoplasm, and translation efficiency [32]. Because we had to group all of our RNA-Seq data to be able to obtain enough poly(A)-containing reads to perform this analysis, we have been unable to study the dynamics of APA in *C. neoformans* and the influence of growth conditions on the regulation of the 3′ end position. Nevertheless, the extent of APA observed in this analysis suggests a major role for APA on *C. neoformans* gene expression regulation.

We next explored the base composition and sequence motifs surrounding the major poly(A) sites of coding genes. We aligned 200-nt sequences surrounding all poly(A) clusters in mRNA 3′-UTRs. The base composition profile is characterized by a small A-rich peak at around −20 nt and a broad T-rich region surrounding the cleavage site (Figure 7C). The A-rich peak at −20 nt could have corresponded to the location of canonical polyadenylation signal sequences found in other model organisms. However, we found AAUAAA motifs in less than 5% of the coding mRNA. The enrichment of C at the −1 nt (Figure 7C) supports the observation that a CA dinucleotide immediately 5′ to the cleavage site is preferred but not absolutely required. Using the DREME software [33], we identified an AUGHAH motif at around the −20 nt (e = 5.9^−1434^), different from the canonical polyadenylation signal in mammalian species [34] (Figure 7D). This AUGHAH motif is highly position-specific at −20 nt (Figure 7E). Interestingly, this motif is also associated with the best efficiency of 3′-end formation (Figure 7F). Finally, while about 76% of the main poly(A) sites contain the motif, AUGCAU is the most prominent variant, representing nearly 28% of the cases (Figure 7D). As noted above, we also identified at least one poly(A) site for 40% of the miscRNAs, among which 65% are associated with the AUGHAH motif. The proportion of the different subtypes is very similar in coding genes and miscRNAs, with AUGCAU being the most commonly found variant in both.

Strikingly, we also identified 317 poly(A) sites in 5′-UTR regions, although only 32% were associated with an AUGHAH motif. Premature 3′ end formation may be involved in the regulation of gene expression, but it might also provide a way to produce short coding RNA. Indeed, the first AUG of the transcript is not the one used as the start codon for ∼50% of the proteins in our annotation. This proportion reaches ∼87% when one considers only 5′-UTRs containing an upstream AUG, suggesting the existence of a number of uORFs associated with regulation in *C. neoformans*.

### Antisense transcription

Strand-specific RNA sequencing revealed that 21% of the genome is transcribed from both strands. Antisense transcription has two main sources, with the first being overlapping transcription of 3′-UTRs caused by tail-to-tail gene arrangements. In fact, out of 2,189 gene pairs oriented tail-to-tail for which both 3′-UTRs have been annotated, 72.7% displayed an antisense overlapping transcription. The overlapping region (median size = 172 nt) is small in some cases and is restricted to only a few bp, but in other cases spans the entire gene. Both overlapping transcripts can be spliced, and the overlapping region does not appear to be restricted by the coding region as recently suggested in *S. cerevisiae* [35]. Although these transcriptomic features are very common, they are difficult to explain, as both RNA types should not be transcribed at the same time from the same genomic locus. Indeed, as shown in *S. cerevisiae*, convergent transcription results in the cessation of RNA polymerase and the production of truncated RNA molecules [36], [37]. Thus, one must imagine a mechanism by which one or the other convergent RNA molecule would be produced alternately from the same genomic locus.

**Figure 8:**
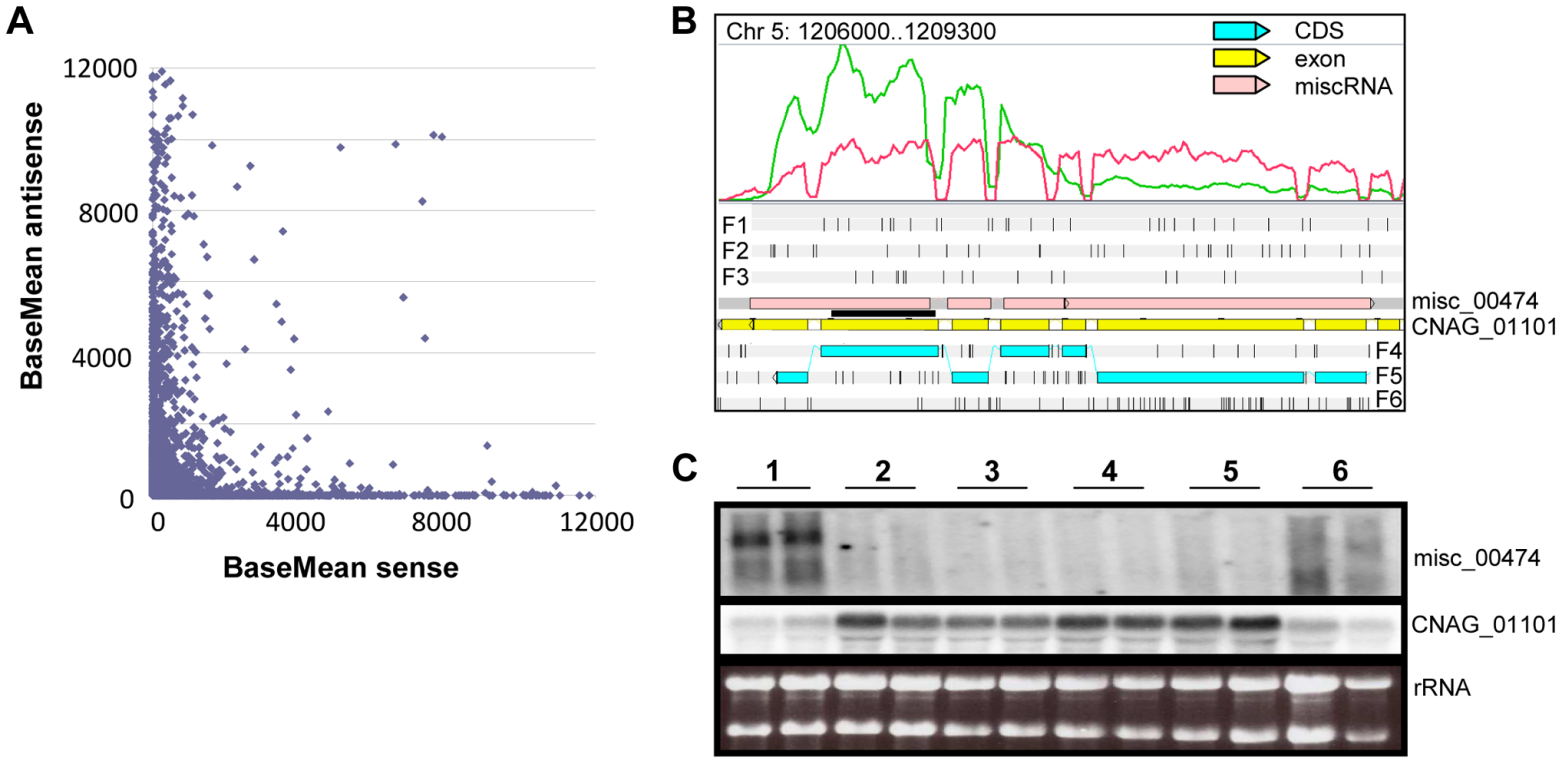
Antisense/sense transcription in *C. neoformans* var. *grubii*. **A.** Comparison of sense/antisense transcription when an antisense transcript is present. Strand-specific data obtained from cells grown on YPD is shown. The BaseMean values represent the normalized reads count for each transcript and measure the level of sense transcription (x axis) and antisense transcription (y axis) as calculated by DESeq [126]. Outliers with a BaseMean above 12,000 were not represented. **B.** Example of differential expression of miscRNA antisense of a coding gene as observed through Artemis. The red curve represents the non-strand-specific coverage observed when cells were grown in YPD to stationary phase at 30°C (condition 1); the green curve shows the non-strand-specific coverage observed when the cells were grown in YPD to log phase at 30°C (condition 2). F1, F2, and F3 stand for 5′ to 3′ frames 1, 2, and 3, respectively. F4, F5, and F6 stand for 3′ to 5′ frames 1, 2, and 3, respectively. The small black vertical bars indicate the position of the stop codons for each frame. **C.** Northern blot obtained after hybridization with strand-specific probes. RNA was extracted from cells growing in YPD (2×10^8^ cells/mL) at 30°C (condition 1), YPD (5×10^7^ cells/mL) at 30°C (condition 2), YPD with 0.01% SDS (5×10^7^ cells/mL) at 30°C (condition 3), YPD with 10 mg/mL fluconazole (5×10^7^ cells/mL) at 30°C (condition 4), YPD (5×10^7^ cells/mL) at 37°C (condition 5), and YP galactose (2×10^8^ cells/mL) at 30°C (condition 6) in duplicate. Then, 5 µg RNA were loaded on a denaturing electrophoresis agarose gel, electrophoresed, and transferred to a nylon membrane. The horizontal black line represents the position of the probes.

The second and major source of antisense transcription results from natural antisense transcripts (NATs). In *C. neoformans* var. *grubii*, most NATs are miscRNAs and do not appear to have any coding capacity, although we identified a few examples where two CDS cross one another (Table S5). The existence of NATs has been previously reported in fungi [38], as well as in *C. neoformans* var. *neoformans* [5]. Different mechanisms of gene regulation (i.e. transcriptional interference, chromatin remodeling, and RNA interference) associated with antisense transcripts have been described in fungi (see [38] for review), and all of them probably occur in *C. neoformans*. First, the global comparison of sense/antisense transcription ratios suggests that high sense transcription is associated with low antisense transcription and conversely, is indicative of global transcriptional interference regulation (Figure 8A). As shown, loci in which both strands are transcribed at the same level are poorly transcribed. Interestingly, we identified some loci in which the sense/antisense ratio is regulated by growth conditions, suggesting a complex mechanism of gene expression regulation (see examples in Figure 8B and Figure S3). For example, in *S. cerevisiae*, the level of transcription of the antisense strand can alter the level of transcription of the sense strand in a histone H3 lysine methylase-dependent pathway [39]. In *C. neoformans*, the regulation of sense/antisense level of expression by the chromatin structure remains to be demonstrated. In addition, double-stranded RNA has been shown to activate diverse RNA interference pathways in *C. neoformans* [40]–[43]. It is thus likely that part of the NATs-dependent regulation depends on these pathways. The formation of these double-stranded RNAs might also regulate mRNA maturation (capping, splicing, and polyadenylation), although this effect has not yet been demonstrated experimentally. Finally, NAT transcription level has been shown to regulate gene expression through chromatin remodeling. For example, in *S. cerevisiae*, the level of transcription of the NAT, named *GAL1ucut*, antisense of the *GAL10-GAL1* locus regulates chromatin structure at this locus and galactose assimilation [44]. A similar *GAL* cluster is present in *C. neoformans* [45]. Interestingly, we also identified a NAT at the *UGE2/GAL10* gene locus in *C. neoformans* (misc_01075), suggesting that a similar mechanism of gene expression regulation could operate in this yeast.

### Centromeres

**Figure 9:**
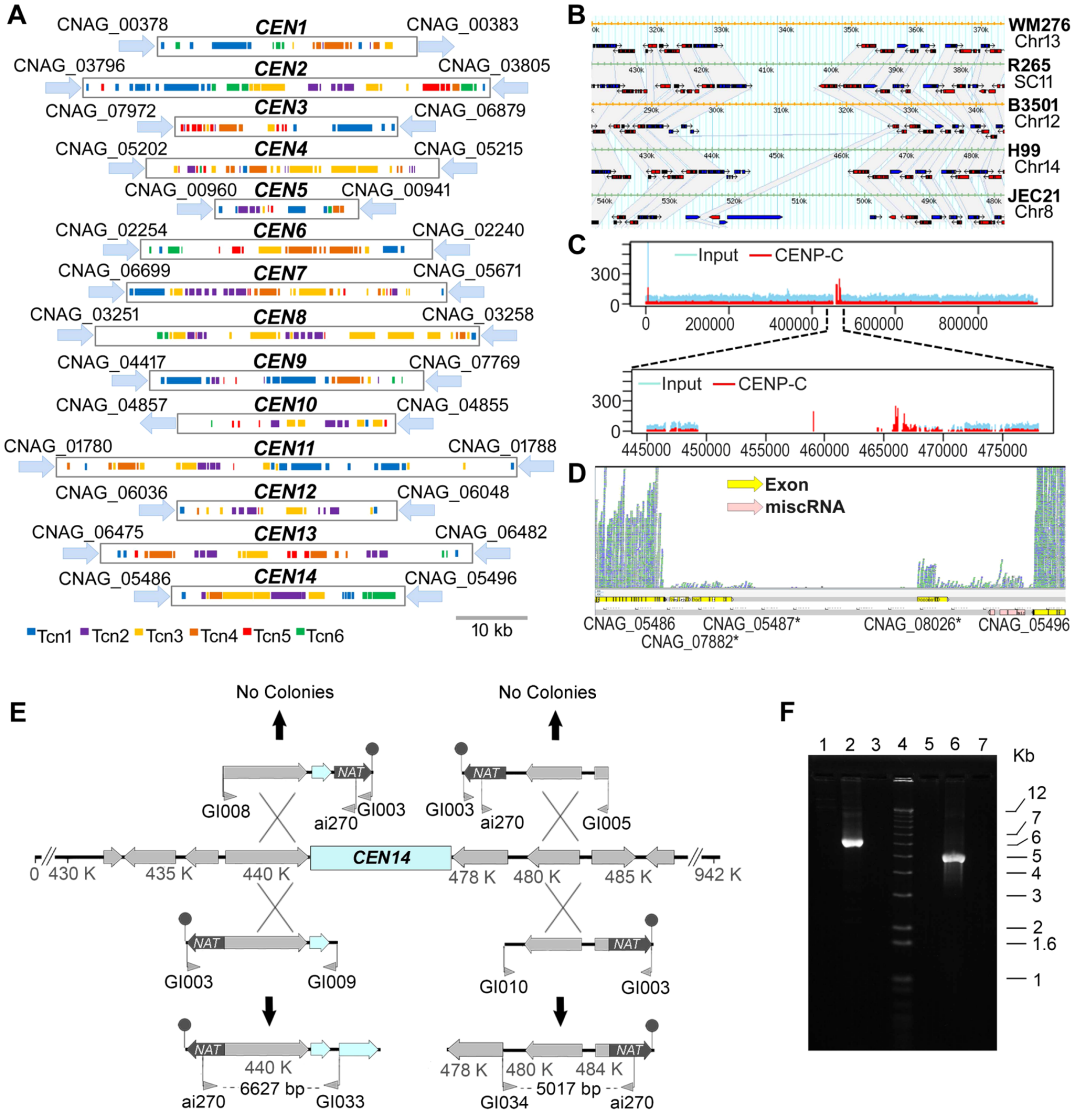
Organization of the centromeres in *C. neoformans* strain H99 and a comparison with other serotypes. **A.** Schematic showing the distribution of transposons, Tcn1–Tcn6, in the presumptive centromeres of all 14 chromosomes of *C. neoformans* strain H99. Each region was identified as the largest ORF-free region on its respective chromosome and contains transposons or its footprints, which are clustered in these sites. **B.** A comparative analysis of the largest ORF-free regions predicted to be centromeres between *C. neoformans* var. *grubii* (H99), *C. neoformans* var. *neoformans* (JEC21 and B3501A), and *C. gattii* (WM276 and R265) using FungiDB reveal conserved synteny of the flanking genes in chromosome 14. The grey color represents the regions that show synteny among different strains. The ORFs present in the centromeric regions are either pseudogenes or have similarity with transposons. **C.** ChIP-Seq analysis showed the enrichment of a conserved kinetochore protein, CENP-C, at the centromeric regions. Here, the enrichment on centromeric region of chromosome 14 (*CEN14*) is shown. The upper panel shows the enrichment on the whole chromosome. In the lower panel, the putative centromeric region is enlarged to show the enrichment profile of CENP-C. **D.** RNA-Seq analysis reveals the absence of poly(A) RNA from *CEN14*. **E.** Targeted truncation mutagenesis on either side of the *CEN14* centromere DNA. Four DNA fragments were produced and transformed into a diploid strain of *C. neoformans*. The stick-and-ball represents the telomeric seed sequence added to the constructs by amplification with primer GI003. No targeted recombination was observed for two constructs, whereas the other two PCR analyses indicated integration of the DNA in those regions. **F.** PCR confirmation of recombination. Lanes 1–3 contain PCRs with primers ai270-GI033, and lanes 5–7 contain PCRs with primers ai270-GI034. Lanes 1 and 5 are amplification results from the diploid strain AI187; lanes 2 and 6 are from strains with integration on the left and right sides, respectively; and lanes 3 and 7 are negative PCR controls. Lane 4 is the Invitrogen 1 kb+ size marker.

The centromere is essential for accurate segregation of replicated chromosomes. Despite its conserved role in chromosome segregation, the underlying centromere DNAs are highly variable in length, sequence, and organization, even among related species [46]–[48] Studies on centromeric regions of different ascomycetous fungi revealed that centromeres are rapidly diverging loci in the genome [47], [49] and may be a driving force for speciation [46]. However, centromeres have not been identified in any basidiomycetous fungi, including *C. neoformans*. Previously, the largest intergenic regions on each of the 14 chromosomes in *C. neoformans* var. *neoformans* (strain JEC21) have been suggested to be putative centromeres [5]. In the present study, we identified the largest intergenic regions on each of 14 chromosomes in the H99 strain, which span from 20 to 65 kb, as the presumptive centromeres (Figure 9A, Table S6). Our sequence analysis of these regions showed an enrichment of transposable elements (Tcn1-Tcn6) or their remnants. An abundance of Tcn transposons is found at the predicted centromeric regions of JEC21 as well. It is important to note that ORFs with similarities to transposons have not been considered to be true ORFs (Figure 9A).

The presence of Tcn-rich centromeres prompted us to compare the flanking regions of *CEN14* in *C. neoformans* var. *grubii* (strain H99) and *C. neoformans* var. *neoformans* (strains JEC21 and B3501). Using FungiDB (http://fungidb.org/fungidb/) [50], this analysis revealed that synteny of the genes across the flanking regions is largely maintained across *Cryptococcus* species (though they were present on different chromosomes in respective strains) (Figure 9B). We also found synteny of these regions with *C. gattii* strains WM276 and R265 (Figure 9B), even though the R265 genome is not completely assembled.

Subsequently, three lines of evidence obtained by ChIP-Seq, RNA-Seq, and chromosome truncation experiments validated *CEN14*, one of the presumptive *CEN* regions, as the functional centromere. First, we performed chromatin immunoprecipitation (ChIP) assays in a strain derived from KN99**a** where CENP-C, a conserved kinetochore protein, was tagged with mCherry [51]. ChIP-Seq analysis revealed that CENP-C-mCherry specifically bound to a single gene-devoid region on chromosome 14 that coincides with the predicted *CEN14* in the H99 strain (Figure 9C). Because CENP-C proteins are known to bind to the functional kinetochores in a variety of organisms, this result confirmed that *CEN14* is indeed the centromere of chromosome 14. Second, the RNA-Seq analysis showed the near absence of mRNA transcription in this region (Figure 9D). The RNA-Seq analysis for other chromosomes showed a similar low level of transcription in all predicted centromeres (Figure S4), suggesting that these regions are largely transcriptionally silent. Finally, the centromere of chromosome 14 was selected and tested for its ability to support stability of the chromosome. Constructs were designed in which a fragment flanking either side of *CEN14* targeted the nourseothricin acetyltransferase (*NAT*) via one crossover event through an approach described previously for *S. cerevisiae* [52]. These constructs also included a terminal stretch of 11 copies of 5′-TTAGGGGG-3′ as a seed sequence for telomere formation that would help stabilize the truncated chromosome. Four constructs were produced, with the *NAT*-telomeric sequence on either side of the targeting region (Figure 9E, F), and transformed by biolistic transformation into the diploid strain AI187, which is derived from H99 variants KN99**a** and a precursor of H99O [53]. Use of a diploid strain avoided the potential problem of loss of essential gene functions, which could occur in a haploid background. Nourseothricin-resistant transformants were obtained, and the targeted integration to the flanks of *CEN14* was tested by PCR. Two constructs, one on each side of *CEN14*, were targeted successfully (Figure 9E). In contrast, the two other constructs, which would generate a chromosome without the putative *CEN14*, were not targeted to that location. These data further support that the intermediary region of chromosome 14 can support segregation of the chromosome, in accordance with its role as a centromere. However, these strains exhibited unstable drug resistance, and pulsed-field gel electrophoresis analysis to resolve chromosomal changes was unable to detect the formation of new, smaller chromosomes in these strains, most likely reflecting instability of these artificial chromosomes.

Some chromosomal rearrangements, including the inversions, appear to be mediated by ectopic recombination between homologous sequences. For example, the inversion denoted by star 1 (Figure 2) is flanked by two alanine tRNAs that likely recombined to produce this inversion in H99. Interestingly, when comparing H99 and WM276, out of the six translocations that we identified, two (between chromosomes 1 and 2 and between chromosomes 4 and 5) seem to have been mediated by, and occurred within, the centromeres, as they resulted in the exchange of chromosomal arms between the two chromosomes (Figures 2B and 2C). The translocation between chromosomes 1 and 2 is also present when H99 is compared to R265, suggesting it is an ancient chromosomal rearrangement that might be shared by *C. gattii*. It is not clear, although it appears likely, that the event between chromosomes 4 and 5 is also present in R265, as the genome of R265 has yet to be assembled into complete chromosomes. Additionally, there is another unique chromosomal translocation in R265 that appears to also have involved the centromere (between H99 chromosomes 3 and 11, Figure 2C). Previous studies have shown that in *C. neoformans* and *C. gattii*, some chromosomal translocation breakpoints are associated with transposable elements [6], [10]. *C. neoformans* and *C. gattii* centromeres are chromosomal regions enriched in transposable elements. Thus, it is not surprising that they might have been involved in chromosomal translocations other than the fact that recombination was traditionally thought to be repressed within centromeres. This has been challenged recently by findings of gene conversion within centromeres in maize and *Candida albicans* [54], [55]. Thus, our results suggest that centromeres, especially regional centromeres, might be more fluid than anticipated. Ectopic recombination between centromeres of non-homologous chromosomes could have dramatic effects on the fitness and evolutionary trajectories of the resulting progeny and play an important role in shaping genome architecture during evolution. The rearranged chromosomal arms could pose a significant reproduction barrier, thus facilitating diversification and possibly eventual speciation.

It has been recently shown that the *CEN* regions are primary sites of siRNA production to silence transposons [42]. Moreover, such transposon silencing is known to occur via the RNAi pathway during sexual reproduction [41], [43]. On the other hand, the RNAi machinery plays a crucial role in centromere function by establishing pericentric heterochromatin in the fission yeast *Schizosaccharomyces pombe*. Mutations in genes encoding the RNAi machinery, such as Ago1, Rdp1, and Dcr1 [56], [57] affect centromere silencing and impair centromere function. It has been recently reported that sexual reproduction increases chromosomal aberrations, including aneuploidy, in *C. neoformans* [58]. Thus, it will be intriguing to determine the effect of silencing on pericentric heterochromatin formation and chromosome segregation during meiosis in *C. neoformans*.

### Replication origins

We next characterized the origins of replication in *C. neoformans*. Preliminary experiments have demonstrated that linear plasmids [59], [60] can not be used to identify replication origins in *Cryptococcus* (Figure S5). We thus used a gel strategy developed by Hamlin and colleagues [61] to enrich for replication bubble-containing DNA to identify *Cryptococcus* replication origins. First, we examined the enriched DNA for the presence of the 3.0-kb *Eco*RI fragment containing the non-transcribed spacer of the multicopy rDNA repeat and confirmed the presence of a replication origin, as it is present in most other eukaryotes examined to date (not shown). We also cloned seven additional independent fragments of chromosomal DNA containing replication origins. These replication origins were mapped to five different chromosomes of *C. neoformans* var. *neoformans* strain JEC21 (Table S7) and all but one of them were conserved in *C. neoformans* var. *grubii* genome sequences.

**Figure 10:**
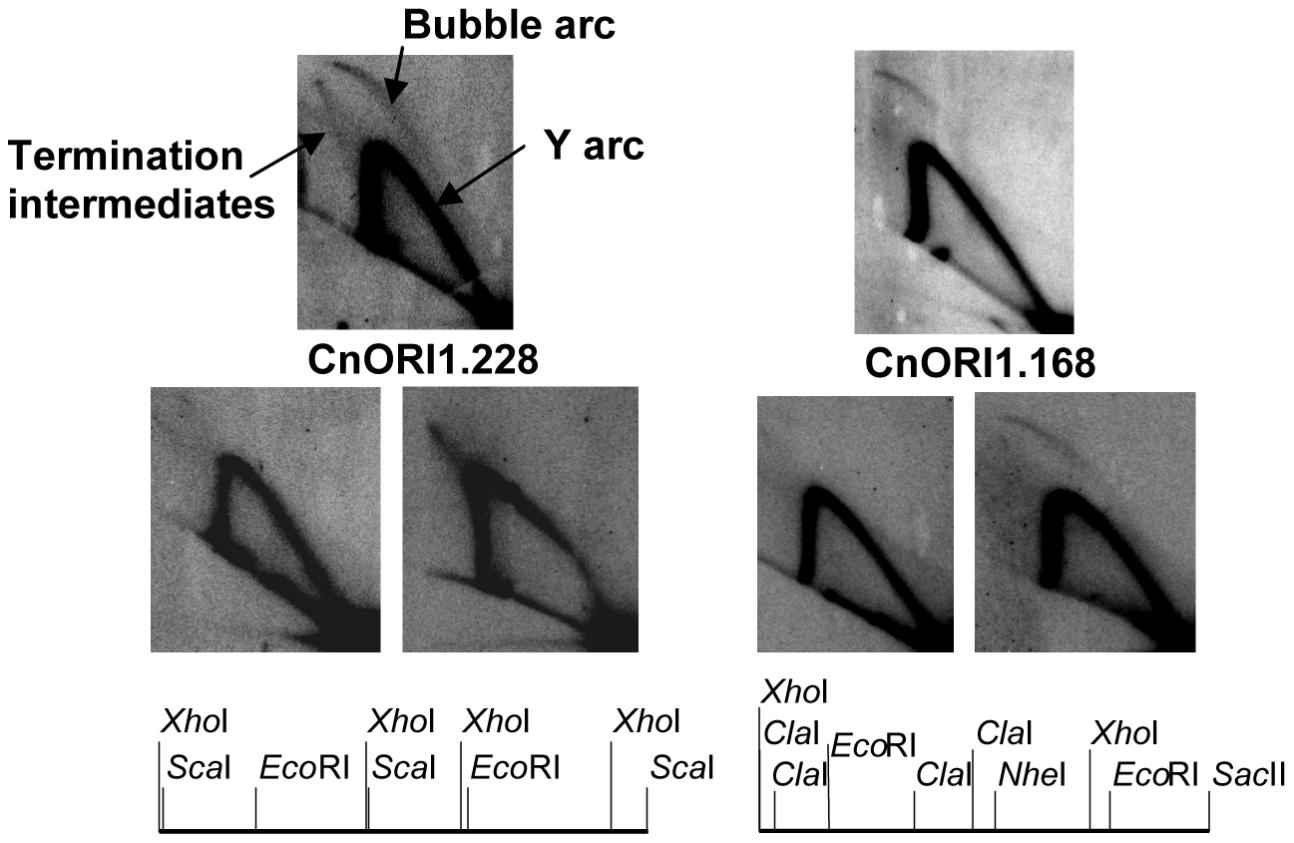
Identification of replication origins. The two sets of three panels show 2D gel patterns of replication intermediates in the regions of CnORI1.168 and CnORI1.228, as diagramed below. The arcs of bubble-shaped replication intermediates, Y-shaped replication intermediates, and replication intermediates are labeled on the upper left panel. The upper autoradiogram in each set shows the 2D gels of the *Eco*RI fragments that defined the origins. The two lower autoradiograms in each set show 2D gels of genomic restriction fragments that overlap the *Eco*RI fragments, also shown in the diagrams below. CnORI1.228: upper panel, 4,722-bp *Eco*RI fragment; lower left panel, 4,655-bp *Xho*I fragment overlapping left end of *Eco*RI fragment; lower right panel, 6,297-bp *Sca*I fragment overlapping right end of EcoRI fragment. CnORI1.168: Upper panel, 5,728-bp *Eco*RI fragment; lower left panel, 4,803-bp *Xho*I-*Nhe*I fragment overlapping left end of *Eco*RI fragment; lower right panel, 4,810-bp *Cla*I-*Sac*II fragment overlapping right end of *Eco*RI fragment. See text for details.

The 2D gel patterns of replication intermediates, which show both complete bubble arcs and strong complete Y-arcs, indicate that all of the replication origins are inefficient and are probably used in ≤25% of cell cycles (Figure 10). The pattern of inefficient origin usage distinguishes *Cryptococcus* from *S. cerevisiae*, in which many replication origins are used efficiently [62]. The pattern of origin use in *Cryptococcus* is more similar to *S. pombe* [63] or mammalian cells [61]. In mammalian cells, the apparent inefficient use of replication origins frequently reflects the presence of large replication initiation zones, in which replication initiates relatively inefficiently at many different sites within the zone. To determine whether *Cryptococcus* has replication initiation zones, we analyzed restriction fragments overlapping six of the origins that we identified (see Figure 10 for two examples). Three of the six generated patterns like CnORI1.168, in which a complete bubble arc was observed in one of the two overlapping fragments. These patterns indicate that these three fragments are parts of initiation zones. The three other replication origins yielded patterns similar to CnORI1.228 with no evidence of bubble arcs in the overlapping fragments. Replication termination intermediates were present in the 2D gel pattern of the *Eco*RI fragment containing CnORI1.228, and the termination signal was stronger in the overlapping fragment to the right, indicating the presence of a replication termination zone in addition to the replication origin in this region of chromosome 1.

Like centromeres, replication origins are required for chromosome maintenance, but their underlying sequences have diverged rapidly despite the conservation of the proteins that make up the DNA replication machinery, including replication initiation proteins that are recruited to replication origins. At one extreme, replication origins of *S. cerevisiae* and closely related strains are short sequences that recruit the replication initiator protein, Origin Recognition Complex (ORC), and feature an easily unwound region of DNA. At the other extreme, mammalian origins are within initiation zones ranging in size from a few kb to several hundred kb. The replication origins identified in *C. neoformans* provide the first view of origins in the Basidiomycota. In addition to evidence for at least small initiation zones in *Cryptococcus* chromosomes, plasmid replication is reminiscent of plasmid replication in mammalian cells in the sense that replication may initiate throughout the plasmid (Figure S5). Although the requirement of telomeres for extrachromosomal plasmid maintenance is unusual, other fungi, including *Histoplasma capsulatum* and *Fusarium oxysporum*, have been shown to maintain foreign DNA as linear plasmids with telomeres ([64] and references therein).

Despite the huge variation in the structure of eukaryotic replication origins, their spacing in chromosomes (50–100 kb) is similar in eukaryotes, and the protein components of the replication machinery are generally highly conserved. The role of replication origins is to assemble a prereplicative complex (preRC) that can be triggered to initiate replication during S phase by regulatory phosphorylation events (reviewed by [65]). We identified probable orthologs for all preRC proteins except Orc6 (Supplemental Table S8). Like *S. cerevisiae*, *Cryptococcus* has a third paralog of Orc1, which encodes the Sir3 protein in budding yeast [66]. It remains to be seen whether the apparent absence of Orc6 from *Cryptococcus* is real or whether it reflects the poor amino acid sequence conservation of Orc6. It is clear from several examples that Orc subunits have diversified among well-studied organisms to also interact with other proteins [67].

### Analysis of phenotypic variation in H99 passaged isolates

**Figure 11:**
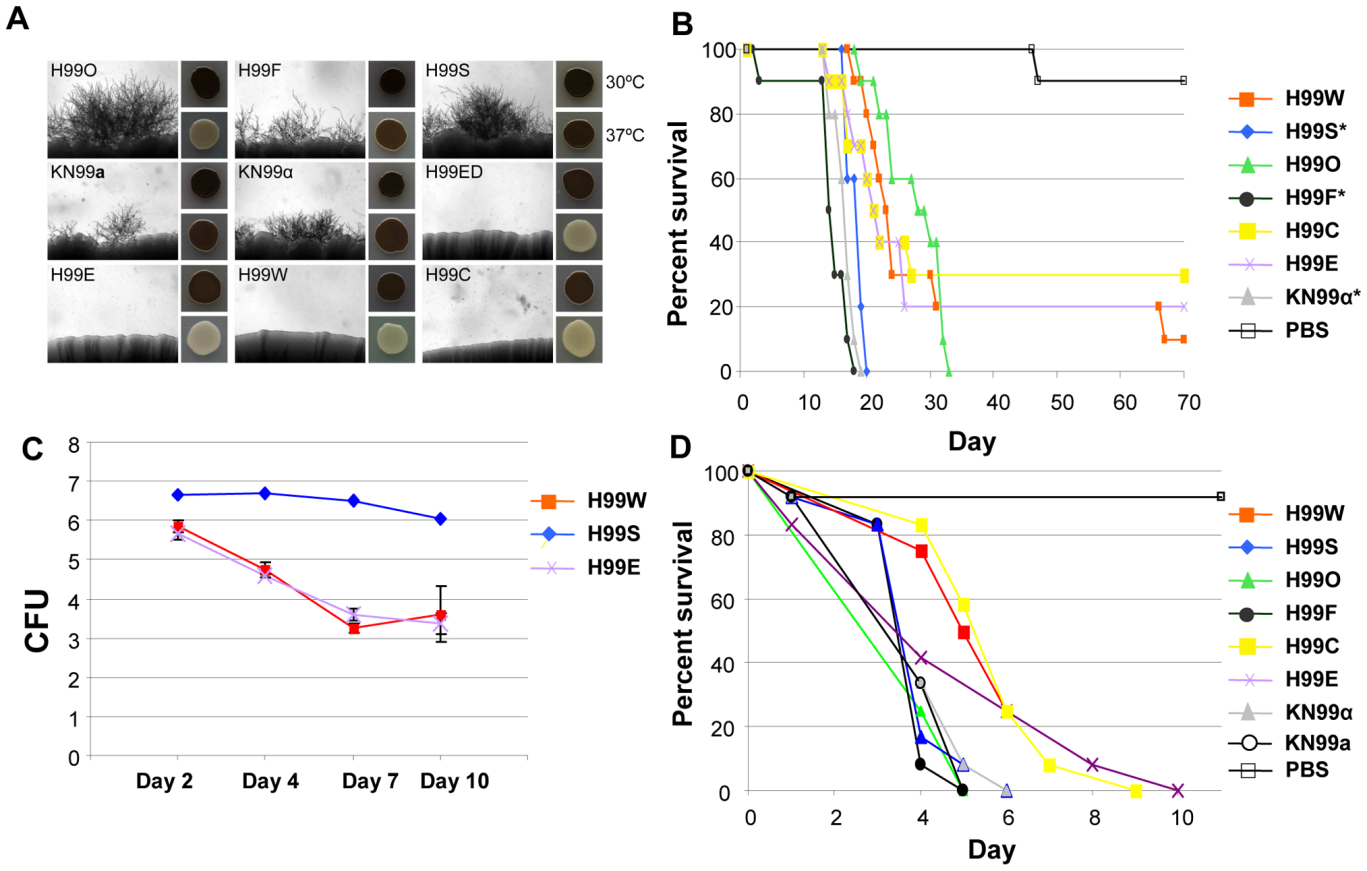
H99 passaged strains exhibit phenotypic variability. **A.** Mating assays on V8 agar were incubated at room temperature for seven days in the dark. Each strain was mated with KN99**a** (except KN99**a**, which was mated with KN99α). Melanization assays were conducted on l–DOPA agar incubated at 30°C or 37°C for two days. **B.** H99 variants differ in virulence in the murine model of infection. A group of 10 animals was each infected with an inoculum of 5.0×10^5^ cells via intranasal instillation for each strain. The results illustrate virulence variations between these well-defined H99 lineage isolates. A PBS control in which no cells were inoculated was also included. We compared the survival data for the seven strains using the Kaplan-Meier method. The significance of the pairwise comparisons to H99O was determined by the Mantel-Cox log rank test. The average time of survival was significantly shorter for the H99S, H99F, and KN99α strains compared to H99O. The survival times of the other strains compared to those of H99O were not significantly different. **C.** H99 variants differ in virulence in the rabbit CNS model of infection. For each of the variants (H99S, H99W, and H99E), three rabbits were infected directly into the CNS. All rabbits were immunosuppressed with steroid treatment. Spinal taps were taken on days 2, 4, 7, and 10 and measured for CFU (log scale). All animals were euthanized at the conclusion of the experiment. **D.** H99 variants differ in virulence in a heterologous host model of infection. For each strain, a group of 12 *Galleria mellonella* larvae was infected with an inoculum of 1.0×10^5^ cells. Survival was monitored and plotted daily for 10 days. Isolates were significantly virulent (*p*<0.005) in comparison with the mock control (sterile PBS) infection, and isolates H99C and H99E were significantly less virulent than the H99O reference strain (*p*<0.05).

Since the *C. neoformans* var. *grubii* type strain H99 was originally isolated in 1978 at Duke University Medical Center (Figure 1; Text S1), laboratory passage has led to the establishment of a number of known lineages with their own distinct phenotypes. All of these variants were derived from the original H99 sequenced isolate (H99O), which was frozen in 1994. For example, the variants H99S and H99F were isolated after passage of a mixed frozen stock through the well-validated rabbit model of central nervous system (CNS) infection. KN99α and KN99**a** are congenic strains obtained after backcrossing using the H99F isolate [68]. As shown in Figure 11B, H99S, H99F, and KN99α appeared to be significantly more virulent than the other strains in a mouse model of infection. This hyper-virulent phenotype of the H99S strain was confirmed in a rabbit model of meningoencephalitis (Figure 11C), but not in the insect *Galleria mellonella* model (Figure 11D), in which these three strains appeared to be as virulent as the H99O strain.

Some other strains, including H99E, H99W, and H99C, originated following laboratory passage at various institutions. These isolates are impaired for melanin production and are virtually sterile in genetic crosses (Figure 11A). They also appear to be less virulent than other strains in all animal models tested (Figure 11B, 11C, and 11D), although this difference was not significant in the mouse model of infection. A description of the origins of each of these strains is given in Figure 1. Overall, nine H99 variants with unique phenotypes have been identified: H99O, H99S, H99C, H99F, H99ED, KN99**a**, KN99α, H99W, and H99E.

We assembled the set of H99 variants from different laboratories and examined phenotypes that would relate to pathogenesis, sexual reproduction, and survival in the wild. The strains differ in melanization, mating capacity, and virulence in three different animals models of cryptococcosis (Figure 11). In addition, the different H99 passaged strains exhibit significant phenotypic variation in environmental stress responses, antifungal drug resistance (Figure S6), and urease production (Figure S7).

Laboratory passage is an inevitable consequence of microbiology research, and there are many anecdotal accounts in the fungal research community of passaged isolates having reduced pathogenicity or fertility. This property has been previously recognized in passaged isolates of a var. *neoformans* strain [69]. The collection of phenotypically characterized H99 variants provides a unique opportunity to understand the molecular, genetic, and possibly epigenetic events that underlie the observed phenotypic variation in *C. neoformans*. As noted above, different versions of this isolate were used to sequence the genome (H99O), construct a congenic strain pair (KN99**a**/α, derived from H99F) [68], construct large-scale mutant libraries (derived from H99C) [70], and a genomic tiling array (H99C) [70]. Thus, providing additional insights into the nature of the changes that have occurred will considerably advance the field, both technically for research on *C. neoformans* and to understand what changes occur during *in vitro* passaging.

The differences between the H99 strains were ascertained through two approaches: one focused on resolving chromosomal length DNA polymorphisms and the other on genome resequencing. Electrophoretic karyotypic analysis via PFGE revealed consistent chromosome sizes with just a small reduction in the size of chromosome 9 observed in H99ED and H99C (Figure S8). Probing of the left and right telomeres of this chromosome identified that while the left subtelomere fragments were identical in length for all eight strains tested, the right subtelomeres of H99ED and H99C were ∼25 kb smaller (Figure S9). The new chromosome endpoints in these strains were characterized via PCR. The missing region contains nine genes that currently lack functional annotation and all of which have duplicates elsewhere in the genome.

Strains H99ED, H99W and H99S, were sequenced to 30× coverage using 72-nt paired-end Illumina reads. Comparison revealed 11 single nucleotide polymorphisms (SNPs) and 11 insertion/deletion (indel) events acquired in the passaged isolates (Table S9), enabling a more detailed pedigree of the H99 series to be determined (Figure 1). To identify which of these mutations conferred the phenotypic changes observed in the H99 series, a cross between the attenuated, largely infertile, less melanized isolate H99C and the virulent, fertile, melanized strain KN99**a** was undertaken. Twenty-seven F1 meiotic progeny were obtained from rare sexual reproduction events and characterized phenotypically and genotypically for the mutations identified in the parental strains (Table 2, Figure S10). Multiple linear (melanization phenotype) and multinomial logistic (mating phenotype) regression analyses revealed linkage between indels 2 and 3 and reduced mating and melanization (mating *p*<0.0001; melanin *p*<0.001). In contrast, no linkage was observed between any phenotype analyzed and the presence or the absence of a truncated chromosome 9 (Table 2).

**Table 2:** Genotypes and phenotypes of progeny set.

Isolate	SNP1	SNP2	SNP4	SNP5	SNP7	SNP8	SNP9	SNP10	Indel1	Indel2	Indel3	Indel4	Indel5	Indel6	Indel7	Indel8	Indel10	Chr 9 genotype	Mating	Melanin
KN99**a**	KN99**a**	KN99**a**	KN99**a**	KN99**a**	KN99**a**	KN99**a**	KN99**a**	KN99**a**	KN99**a**	KN99**a**	KN99**a**	KN99**a**	KN99**a**	KN99**a**	KN99**a**	KN99**a**	KN99**a**	KN99**a**	KN99**a**	8,98
H99C	H99C	H99C	H99C	H99C	H99C	H99C	H99C	H99C	H99C	H99C	H99C	H99C	H99C	H99C	H99C	H99C	H99C	H99C	H99C	1,07
1	KN99**a**	H99C	H99C	H99C	KN99**a**	KN99**a**	KN99**a**	KN99**a**	KN99**a**	KN99**a**	KN99**a**	H99C	KN99**a**	H99C	H99C	H99C	KN99**a**	H99C	H99C	4,15
2	H99C	H99C	KN99**a**	H99C	KN99**a**	H99C	H99C	H99C	KN99**a**	KN99**a**	KN99**a**	H99C	H99C	KN99**a**	H99C	H99C	H99C	KN99**a**	KN99**a**	6,10
3	KN99**a**	H99C	H99C	H99C	KN99**a**	H99C	H99C	H99C	H99C	H99C	H99C	H99C	H99C	KN99**a**	H99C	H99C	KN99**a**	H99C	H99C	7,70
4	KN99**a**	KN99**a**	KN99**a**	KN99**a**	H99C	H99C	H99C	H99C	H99C	H99C	H99C	KN99**a**	KN99**a**	KN99**a**	KN99**a**	H99C	H99C	KN99**a**	No mating	0,66
5	KN99**a**	H99C	KN99**a**	KN99**a**	H99C	H99C	H99C	H99C	KN99**a**	KN99**a**	KN99**a**	H99C	KN99**a**	H99C	H99C	KN99**a**	H99C	H99C	KN99**a**	5,91
6	H99C	H99C	KN99**a**	H99C	H99C	H99C	H99C	H99C	KN99**a**	KN99**a**	KN99**a**	H99C	H99C	H99C	KN99**a**	H99C	H99C	H99C	KN99**a**	4,83
7	KN99**a**	H99C	H99C	KN99**a**	H99C	KN99**a**	KN99**a**	KN99**a**	H99C	KN99**a**	KN99**a**	H99C	H99C	H99C	H99C	KN99**a**	KN99**a**	H99C	H99C	3,60
8	KN99**a**	H99C	H99C	H99C	KN99**a**	KN99**a**	KN99**a**	KN99**a**	KN99**a**	KN99**a**	KN99**a**	H99C	KN99**a**	H99C	H99C	H99C	KN99**a**	KN99**a**	KN99**a**	8,75
9	H99C	H99C	H99C	KN99**a**	H99C	KN99**a**	KN99**a**	KN99**a**	H99C	H99C	H99C	H99C	H99C	KN99**a**	H99C	KN99**a**	H99C	H99C	No mating	0,60
10	KN99**a**	H99C	KN99**a**	KN99**a**	H99C	KN99**a**	KN99**a**	KN99**a**	H99C	KN99**a**	KN99**a**	H99C	KN99**a**	KN99**a**	H99C	H99C	KN99**a**	H99C	H99C	3,53
11	H99C	H99C	KN99**a**	H99C	H99C	H99C	H99C	H99C	KN99**a**	KN99**a**	KN99**a**	KN99**a**	H99C	KN99**a**	H99C	H99C	H99C	KN99**a**	KN99**a**	6,42
12	KN99**a**	KN99**a**	H99C	H99C	H99C	H99C	H99C	H99C	KN99**a**	KN99**a**	KN99**a**	KN99**a**	KN99**a**	KN99**a**	H99C	H99C	H99C	H99C	KN99**a**	8,99
13	KN99**a**	H99C	KN99**a**	H99C	H99C	H99C	H99C	H99C	H99C	KN99**a**	KN99**a**	H99C	KN99**a**	H99C	KN99**a**	KN99**a**	H99C	KN99**a**	H99C	8,62
14	H99C	KN99**a**	KN99**a**	KN99**a**	KN99**a**	KN99**a**	KN99**a**	KN99**a**	H99C	KN99**a**	KN99**a**	KN99**a**	H99C	KN99**a**	KN99**a**	KN99**a**	KN99**a**	H99C	H99C	8,47
15	H99C	KN99**a**	KN99**a**	KN99**a**	KN99**a**	KN99**a**	KN99**a**	KN99**a**	H99C	H99C	H99C	H99C	H99C	KN99**a**	KN99**a**	H99C	KN99**a**	H99C	No mating	0,00
16	KN99**a**	H99C	KN99**a**	KN99**a**	H99C	H99C	H99C	H99C	KN99**a**	H99C	H99C	H99C	KN99**a**	H99C	KN99**a**	H99C	H99C	KN99**a**	No mating	7,30
17	H99C	H99C	KN99**a**	H99C	H99C	H99C	H99C	H99C	KN99**a**	KN99**a**	KN99**a**	KN99**a**	KN99**a**	KN99**a**	H99C	H99C	H99C	H99C	KN99**a**	9,12
18	KN99**a**	KN99**a**	KN99**a**	KN99**a**	KN99**a**	KN99**a**	KN99**a**	KN99**a**	KN99**a**	KN99**a**	KN99**a**	KN99**a**	H99C	H99C	KN99**a**	KN99**a**	KN99**a**	H99C	H99C	9,05
19	H99C	KN99**a**	KN99**a**	KN99**a**	H99C	H99C	H99C	H99C	H99C	KN99**a**	KN99**a**	KN99**a**	H99C	H99C	KN99**a**	KN99**a**	H99C	KN99**a**	KN99**a**	4,89
20	KN99**a**	H99C	H99C	H99C	KN99**a**	KN99**a**	KN99**a**	KN99**a**	H99C	KN99**a**	KN99**a**	H99C	KN99**a**	KN99**a**	H99C	H99C	KN99**a**	H99C	H99C	8,18
21	KN99**a**	H99C	H99C	H99C	H99C	H99C	H99C	H99C	KN99**a**	H99C	H99C	H99C	KN99**a**	KN99**a**	H99C	KN99**a**	H99C	H99C	No mating	0,80
22	KN99**a**	H99C	KN99**a**	H99C	KN99**a**	KN99**a**	KN99**a**	KN99**a**	H99C	H99C	H99C	H99C	H99C	H99C	H99C	H99C	KN99**a**	KN99**a**	No mating	6,44
23	KN99**a**	KN99**a**	H99C	H99C	H99C	H99C	H99C	H99C	KN99**a**	KN99**a**	KN99**a**	KN99**a**	KN99**a**	KN99**a**	KN99**a**	H99C	H99C	KN99**a**	H99C	5,76
24	H99C	H99C	H99C	KN99**a**	KN99**a**	H99C	H99C	H99C	KN99**a**	H99C	H99C	H99C	H99C	H99C	KN99**a**	KN99**a**	H99C	H99C	No mating	0,46
25	H99C	KN99**a**	KN99**a**	KN99**a**	H99C	KN99**a**	KN99**a**	KN99**a**	KN99**a**	KN99**a**	KN99**a**	KN99**a**	H99C	KN99**a**	H99C	H99C	KN99**a**	KN99**a**	KN99**a**	8,32
26	H99C	KN99**a**	H99C	H99C	KN99**a**	KN99**a**	KN99**a**	KN99**a**	KN99**a**	KN99**a**	KN99**a**	KN99**a**	H99C	H99C	H99C	H99C	KN99**a**	KN99**a**	KN99**a**	8,49
27	KN99**a**	H99C	KN99**a**	H99C	H99C	H99C	H99C	H99C	H99C	H99C	H99C	H99C	H99C	KN99**a**	KN99**a**	H99C	H99C	H99C	No mating	1,10

Chromosome 9: KN99**a** contains full-length chromosome 9. H99C contains a shortened version.

Mating: mating productivity on V8 medium at day 7.

Melanin: grey-scale value of melanin spot on l-DOPA medium.

Indel 3 is present on the left arm of chromosome 2 within the 3′-UTR of CNAG_06730, which encodes a predicted CMGC/GSK protein kinase and is likely to be silent. Indel 2 causes a frame shift within the first exon of CNAG_06765, currently annotated as a hypothetical protein (Table S9). CNAG_06765 is predicted to encode a glutamine-rich protein with a dimerization LisH domain. A BLAST search returns homology from only *Cryptococcus*, while an OrthoMCL database search (Group OG5_131426) reveals orthologs in a range of fungi, but many of these contain domains not present in CNAG_06765.

**Figure 12:**
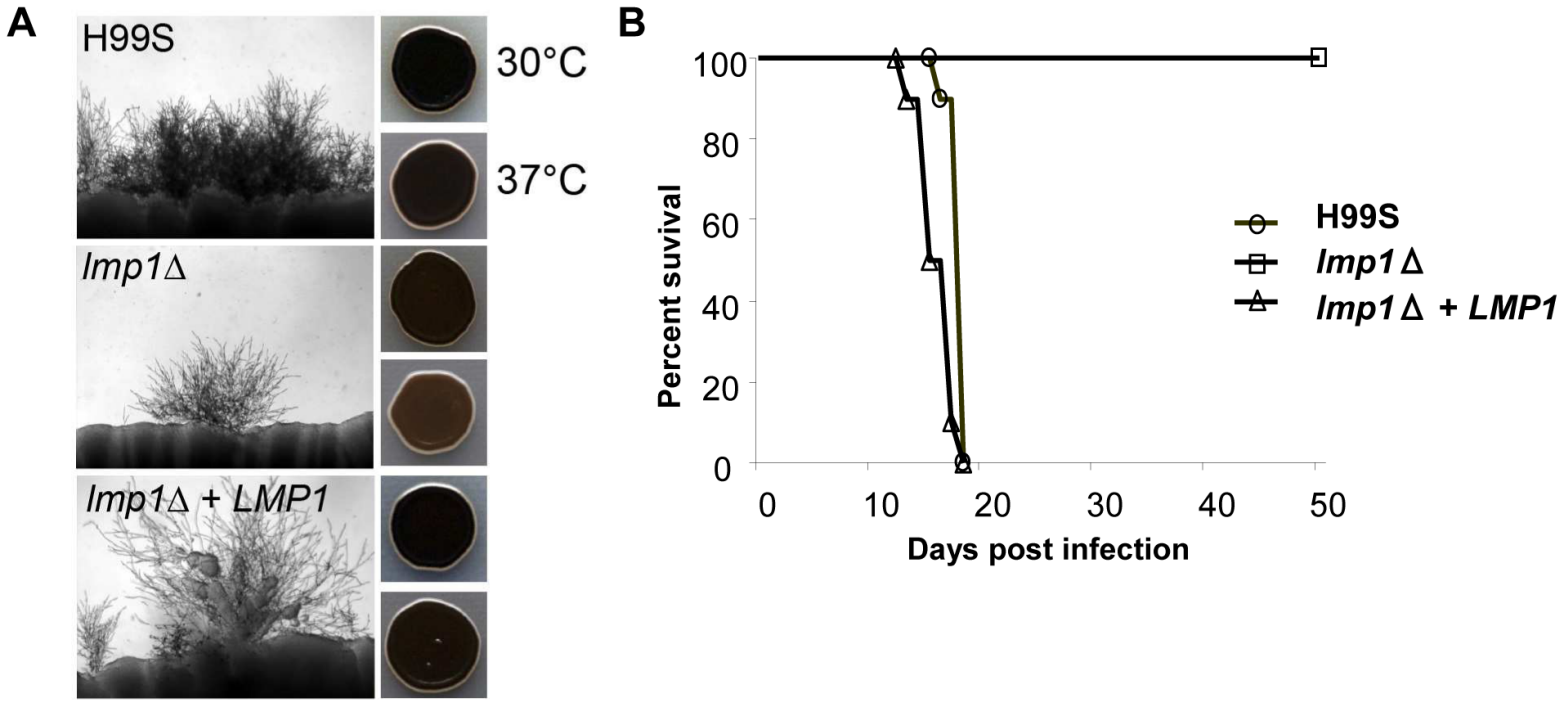
Deletion of *LMP1* reduces mating efficiency, melanization and virulence. **A.** Mating assays on V8 agar incubated at room temperature for 7 days in the dark. Each strain was mated with KN99**a**. One or two mating tufts were observed in the *lmp1*Δ mutant per mating reaction whereas the wild-type and *lmp1Δ*+*LMP1* complemented strain mated robustly. Melanization is reduced in the *lmp1*Δ mutant at 37°C and restored in the *lmp1*Δ+*LMP1* complemented strain in assays on l–DOPA agar incubated at 30°C or 37°C for 2 days. **B.** H99S, *lmp1Δ mutant* strain, and *lmp1Δ*+*LMP1* complemented strain in the murine model of infection. Per each strain, a group of 10 animals was each infected with an inoculum of 5.0×10^5^ cells via intranasal instillation. The results illustrate the complete loss of virulence in the *lmp1*Δ mutant with the virulence restored back to the H99S level in the *lmp1*Δ+*LMP1* complemented strain.

A gene deletion mutant of CNAG_06765 was isolated in the H99S background, which is the most mating-prolific H99 strain. The mutant showed a significant reduction in both mating with KN99**a** and melanization compared to the wild-type H99S control, although neither was completely abolished (Figure 12). For mating, where H99S produced mating hyphae across the entire periphery of each mating reaction, only one or two mating tufts were observed for the CNAG_06765 deletion mutant. We therefore dubbed the gene *LMP1* for *l*ow *m*ating *p*erformance. Interestingly, *lmp1Δ* mutant strains were completely avirulent in a mouse model of infection (Figure 12). As expected, re-introduction of the wild-type *LMP1* gene complemented the *lmp1Δ* mutation and restored the original strain phenotypes, although mating remained less dense than typically observed in H99S (Figure 12). These results suggest strongly that indel 2, which occurred during laboratory passage, is responsible at least in part for the phenotypes of the strains H99ED, H99W, H99C, and H99E. It is also important to stress the strength of whole genome sequencing and comparative genome analyses to identify virulence factors in pathogens [71].

### Conclusions

This study reveals the complexity of the *C. neoformans* transcriptome and the dynamic nature of its genome structure. While the number of sequenced fungal genomes continues to increase, (http://www.ncbi.nlm.nih.gov/genomes/static/gpstat.html), only a few have been fully annotated, and most of this annotation information has been generated through comparison and automatic sequence analysis pipelines [72], [73]. In fact, only a handful of fungal genome annotations relying on strand-specific RNA sequence data and manual curation of gene structures have been published [74], [75]. For this reason, the 3′ and 5′-UTRs as well as antisense transcripts are very rarely annotated and, when they are, the degree of annotation is limited to few loci [38], [76]. The information presented herein on the genome and transcriptome of *C. neoformans* var. *grubii* is exceptional and will most probably serve as a reference genome for a large number of fungal species. Considering the intron density in basidiomycetes [23] and the fact that the current genome annotations available in this genus are mainly based on predictions by bioinformatics (see for example, [77]–[80]) a reference genome annotation supported by deep RNA sequencing data is critical and will be widely used, similar to the genomes and transcriptomes of *S. cerevisiae* and *S. pombe* for ascomycetes. This study also opens new avenues for virulence studies. For example, the role of the miscRNAs in the biology of *C. neoformans* remains to be determined. Finally, due to tremendous progress in sequencing technologies, projects to sequence multiple *Cryptococcus* isolates are ongoing, with more than 400 sequencing projects registered in NCBI for *C. neoformans* and *C. gattii*. These ambitious projects aim to understand the population biology of these pathogens and will undoubtedly utilize the information presented herein as the reference for their studies.

## Materials and Methods

### Ethics statement

This study was performed in strict accordance with the recommendations in the Guide for the Care and Use of Laboratory Animals of the National Institutes of Health. The protocols were approved by the Animal Studies Committee at Washington University (Animal Assurance number A3381-01) and by the Duke University Institutional Animal Care and Use Committee (Animal Assurance number A003-13-01).

### Media

*Cryptococcus* strains were cultured in YPD (1% yeast extract, 2% Bacto-peptone, 2% glucose) and maintained at 4°C on YPD solidified with 2% agar or stored at −80°C in 15% glycerol. Sexual reproduction assays were conducted by mixing each strain with either alternate mating type KN99**a** or KN99α on V8 medium (pH 5) [81] or Murashige and Skoog (MS) medium supplemented with vitamins (*Phyto*Technology Laboratories, Shawnee Mission, KS). l-3,4-dihydroxyphenylalanine (l-DOPA) medium with 10 mM nitrogen source for melanization assays was prepared as described [82]. Niger seed medium was prepared with 700 g ground niger seed, 1 g glucose, and 2% agar in 1 L plus 5 mL Tween 20 (20%). Pigeon guano medium was prepared as previously described [83]. Starvation medium was prepared with 0.2 g glucose, 0.05 g ammonium sulfate, and 0.1 MOPS (morpholinopropane sulfonic acid) in 1 L of 1X YNB (Difco) solution (pH 7.2). Immediately before use, 30 µL of 0.72 M tBOOH (*tert*-butyl hydroperoxide) were added to each liter of medium.

### Genome sequencing of H99 strains

#### Sanger sequencing and initial annotation

Paired-end sequence was generated using Sanger technology from plasmid, fosmid, and bacterial artificial chromosome (BAC) clones (Table S10). Reads were filtered and assembled using Arachne [84] (assembly version 1). Gap closure of the initial assembly of 210 scaffolds produced a final assembly of 14 scaffolds in which each scaffold corresponds to a unique chromosome (assembly version 2). The centromeric region of chromosome 14 in version 2 was replaced with that of version 1; new junctions and sequences were confirmed using aligned WGS Illumina reads from H99. This updated assembly was released and submitted to NCBI (assembly version 3).

An initial gene set from this assembly was generated by combining the predictions from *ab initio* methods, sequence conservation, cDNA sequence, and mapping gene calls predicted for the initial Arachne assembly. Expressed sequence tags (ESTs) obtained from GenBank (in 2008) were aligned to the genome using BLAT [85]. Gene structures were predicted directly from aligned ESTs. Conserved coding loci were identified based on BLAST [86] sequence similarity of the genome to the non-redundant protein database (downloaded from NCBI on August 1, 2007). Gene models were predicted using GeneMark [87], which is self-training. Gene models matching full length EST-derived ORFs were used to train Augustus [88] and Twinscan [89]. Results from the *ab initio* gene prediction programs Geneid [90] and Glean [91] were also incorporated. Genes predicted for the initial draft assembly using Genewise [92] (trained with the JEC21 *C. neoformans* serotype D gene set), Twinscan (trained on the genome with a smoothed empirical model of intron length distribution), and Glean were transferred to the chromosome-based assembly using alignment coordinates that were generated using PatternHunter [93]. The best gene model at each locus was selected computationally based on concordance with EST and BLAST evidence. Genes with aberrant structures were examined and corrected manually. Probable repetitive elements were removed, including genes overlapping with RepeatMasker (http://www.repeatmasker.org) hits, genes with Pfam domains known to occur in repetitive elements, and genes with BLAST similarity to a locally maintained repeat library. Additional probable repeats were identified using a BLAST self-alignment of the draft gene set to the genomic sequence, requiring 90% nucleotide identity over 100 nt; genes that matched other locations in the genome eight times or more were removed. Gene models whose best BLAST hit was a repetitive element were also removed. With all of these filters, genes with non-repeat Pfam domains were retained in the gene set. This resulted in an initial set of 6,697 predicted protein-coding genes (Broad Institute release 12/08/2008 version 4). Ribosomal RNA features were manually annotated based on RNAMMER [94] and BLAST similarity with ribosomal RNA features (downloaded from GenBank on 10/2/2006); tRNA features were annotated using TRNAscan-SE [95]. The genome sequence data has been deposited in NCBI (see Table S11).

#### Genome sequencing of the H99 passaged strains

Paired-end reads (72 nt) were generated for H99S, H99E, and H99W to 30× coverage. Reads were mapped using BWA 0.5.9 [96] using the H99 genome as a reference. BWA was run with default settings. Marking of duplicate reads, realignment of reads around indels, and recalibration of quality scores were then undertaken following the Genome Analysis Toolkit (GATK) pipeline, culminating in SNP and indel detection [97], [98]. The centromeres were excluded from this analysis. Further variation detection was undertaken using BreakDancer [99], CREST [6], and Dindel [100]. Sanger sequencing for progeny genotyping was undertaken at BGI (Shenzhen, China) and the Australian Genome Research Facility (Brisbane, Queensland) and analyzed using CLC Genomics Workbench (5.5, CLC bio, Denmark). These genome sequence data have been deposited in NCBI (see Table S11).

### RNA extraction, sequencing, and read analyses

Total RNA was extracted from *C. neoformans* strain KN99α cells grown under different conditions using a previously described protocol [45]. We performed each extraction experiment in independent duplicates.

#### 100-bp paired end sequencing and alignments

For high-throughput sequencing, paired-end cDNA libraries were prepared from 10 µg of total RNA using the Illumina mRNA-Seq-Sample Prep Kit according to manufacturer's instructions. cDNA fragments of ∼400 bp were purified from each library and confirmed for quality by Bioanalyzer (Agilent). Then, 100 bp were sequenced from both ends using an Illumina HiSeq2000 instrument according to the manufacturer's instructions (Illumina).

Quality-based trimming of reads was performed using an in-house perl script. According to the HiSeq2000 outputs, it is not possible to have confidence in a base with a «B» quality (Phred p-value = 2). Therefore, we systemically removed the first base of all reads because of low quality and then trimmed every read according to the position of the first «B». We kept paired reads only if both mates were ≥70 bp. Reads from each dataset were aligned to the 14 *C. neoformans* H99 chromosomal contigs using TopHat [13]. TopHat was set to detect between 30 and 4,000 nt in length. We compared two different versions of TopHat (version 1 and version 2). TopHat1 displayed higher sensitivity whereas TopHat2 had higher specificity. In fact, one main advantage of TopHat1 over TopHat2 is its ability to detect introns surrounding short exons. On the other hand, the number of reads aligned to the genome was higher with TopHat2. Therefore, we decided to use a two-step mapping procedure. We first ran TopHat1 to detect the widest intron population and then applied TopHat2 (microexon search activated, min-intron-length = 30 nt; min-coverage-intron = 30 reads, min-segment-intron = 30 nt, max-intron-length = 4,000 nt, max-multihits = 1 nt), supplying it with an up-to-date set of gene model annotations. These RNA-Seq data have been deposited in the NCBI database (Table S11).

#### Strand-specific RNA sequencing and gene structure improvement

RNA was prepared from two biological replicates each of H99O cultured in three conditions: YPD, starvation medium, and pigeon excreta broth (PG) media. Strand-specific libraries were constructed for poly(A)-selected RNA samples using the dUTP second strand marking method [101], [102] as previously described [103]. Libraries were sequenced on an Illumina HiSeq to generate an average of 76 million paired-end reads (101 nt) per sample.

The sequence from one replicate of each condition was compared to the updated gene set described above. First, reads were assembled using Inchworm [104] by first aligning the reads to the H99 genome using BLAT. Inchworm assemblies were then used by PASA to update protein-coding gene structures. Novel predicted genes (for example, CNAG_00229, CNAG_20209, and CNAG_06049) and merged genes (for example, CNAG_07820 and CNAG_07821 were merged to generate CNAG_20182) were manually reviewed to confirm these predictions. The RNA-Seq data suggested alternative splice sites and translation start sites, and added UTRs. This update resulted in UTR predictions for 6,738 genes, with a median length of 139-bp 5′-UTRs and 198-bp 3′-UTRs. The final set of 6,962 genes includes 7,813 transcripts, with alternative transcripts for 741 genes (of which 93 have more than two transcripts). These RNA-Seq data have been deposited in the NCBI database (Table S11).

In addition, we used the strand-specific and non-specific RNA-Seq data to identify miscRNAs. miscRNAs were defined as transcript active regions larger than 100 bp, with no open reading frame or only a small ORF (<100 aa) without a BLASTp hit in GenBank (p>10^−20^). The combined gene set, including protein coding genes and miscRNA genes, was submitted to GenBank under accession number CP003820-CP003834.

### Identification of polyadenylation sites

The poly(A) sites were identified as previously described [26]. Briefly, reads containing 5 or more consecutive “A” nucleotides at their end (or “T” at their beginning, which were reverse complemented for subsequent analyses) were selected from each of the libraries, and redundant reads were removed. These non-redundant reads were pooled. The A stretches at the end were trimmed, and reads exceeding 18 nt after trimming were mapped to the reference genome using TopHat2. To distinguish poly(A) tracks of true polyadenylation from poly(A) tracks of internal poly(A) stretches on the mRNAs themselves (i.e. false positives), we analyzed the base composition surrounding the end of the mapped reads and discarded those that might not represent true polyadenylation. Reads with the following properties were regarded as false positives and removed: 1) reads with ≥5 A nt immediately downstream of the terminus; 2) depending on the actual length of the poly(A) stretch of the read (e.g. N nt), reads for which 70% of N nt downstream of the end site are As; and 3) reads with ≥8 A nt within 10 nt immediately upstream of the end site. The polyadenylation site was then defined as the base immediately downstream of the read. To ensure that the identified polyadenylation sites were not false positives derived from low quality base calls, reads with quality scores <20 in any of the 5 nt flanking the polyadenylation site were removed. These procedures should serve to remove false positives derived from internal poly(A) stretches and low quality base calls.

### Assigning the poly(A) site clusters to gene models

As most of the observed polyadenylation sites appeared as clusters, we grouped the poly(A) sites into clusters by allowing an optimal maximum intra-cluster distance (at 15 nt) between sites. A poly(A) cluster was then represented by the poly(A) site with the highest number of supporting reads (i.e. peak), and these peak positions were reused in all downstream analyses. A poly(A) cluster was defined as valid when the number of reads at the peak position was ≥2. To assign poly(A) tails to mRNAs, we first searched for poly(A) clusters within 800 nt downstream of their stop codons on the same strand and recorded the size of the coverage gap between the poly(A) clusters and the stop codon. Introns in UTRs were excluded from coverage gap consideration. A poly(A) tail for an mRNA was defined as valid when coverage of a gap was ≤50 nt. Finally, we manually curated this information through visual examination of the read alignments. Length of the 3′-UTR of an mRNA was defined as the distance between the farthest valid poly(A) clusters and its stop codon.

### Discovering sequences motifs for polyadenylation

The sequences immediately upstream and downstream (50 nt on each side) of the poly(A) site of all mRNAs were used to scan for conserved motifs using DREME [33]. DREME performs discriminative discovery of motifs that are enriched in a positive set in comparison to a negative set. The sequences immediately upstream or downstream were thus used as the positive sets, and the upstream (at position −200) or downstream (at position +150) sequences of the same length were used as the negative sets. A highly stringent E-value (10^−50^) was chosen to avoid spurious motifs. To investigate the positional enrichment of these discovered motifs surrounding the polyadenylation sites, the total occurrence of these motifs was searched along the sequences surrounding (200 nt) the poly(A) sites.

### Differential expression analysis

We used differential expression analysis scripts in the Trinity pipeline [104], [105] to process the strand-specific RNA-Seq data generated from three conditions (pigeon guano, starvation media, and rich media [see above]), with two biological replicates from each condition. We aligned the RNA-Seq reads to full transcript sequences (including UTRs) using bowtie [12]. The alignments were used to quantify transcript abundances by RSEM [106]. Differential gene expression analysis was conducted using edgeR with TMM normalization [107], [108], and p-values were corrected for multiple testing [109]. The FPKM values for the most differentially expressed genes (corrected *p*-value <0.001 and log_2_ fold change >2) were hierarchically clustered using Euclidian distance and complete clustering methods; six clusters of genes with similar expression conditions across these conditions were identified using kmeans clustering.

### Comparative genomics

Protein conservation was examined using ORTHOMCL (version 1.4 with a Markov inflation index of 1.5 and a maximum e-value of 1×10^−5^). PFAM and TIGRFAM domains within each gene were identified with Hmmer3 [110] using the PFAM27 and TIGRFAM13 release versions. Domain counts between genomes were compared using Fisher's Exact test, with q-value correction for multiple testing [111].

### Pulsed-field gel electrophoresis

Preparation of agarose-embedded intact *Cryptococcus* chromosomal DNA was performed as previously described [112]. Chromosomes were separated in 1% pulsed-field certified agarose gels using a CHEF-DRIII pulsed-field gel electrophoresis system (Bio-Rad, Richmond CA) in 0.5× TBE running buffer. Running conditions were as follows: ramped switch time from 1.5 sec to 10 sec, 120°, 6 V/cm, 24 h, performed at 14°C using a Bio-Rad cooling module. Chromosomes were stained and visualized with ethidium bromide. Southern blotting of pulsed-field gels was performed as previously described [113] onto Hybond-XL nylon membranes (GE Healthcare, Chalfont St Giles, UK). Blots were UV crosslinked with 100 mJ UV using a Stratagene UV Stratalinker 2400. Radiolabelled probes were prepared using the GE Healthcare Rediprime II Random Prime Labeling System (GE Healthcare) with 20 µCi α-^32^P dCTP (Perkin Elmer, Waltham MA). Hybridizations were performed overnight at 65°C. Probes were detected by exposing the blots to Fujifilm Super RX medical X-ray film (Fujifilm, Tokyo JA).

### Identification of largest ORF-free regions and mapping of transposons in *C. neoformans*

We scanned the genome of *C. neoformans* by using the genome map feature already available in the *C. neoformans* genome database (http://www.broadinstitute.org/annotation/genome/cryptococcus_neoformans/GenomeMap.html) searching for ORF-free regions on each chromosome. This was followed by the determination of the largest ORF-free regions on each chromosome. The DNA sequences of each of the transposons (e.g. Tcn1–Tcn6) have been previously reported [114]. The nucleotide sequences of these Tcn elements were used as query sequences in a BLASTn analysis to identify the transposable elements present in the genome. The BLAST hits against each of the transposons in all chromosomes were obtained and mapped on each of these ORF-free regions.

### Molecular techniques

*C. neoformans* genomic DNA was prepared using the CTAB method [115]. Constructs for targeted replacement of DNA regions in *C. neoformans* were made using overlap PCR with primers listed in Table S12. The *lmp1*Δ mutant strain was isolated in the H99S background by replacing the *LMP1* coding sequence with the neomycin resistance marker from plasmid pJAF1 [116]. For complementation, *LMP1* plus 1 kb flanking region was amplified from the H99S strain, cloned into pCR2.1-TOPO (Life Technologies), and subsequently subcloned into the plasmid pCH233, which contains the nourseothricin resistance marker. Biolistic transformation was performed as previously described [117].

The constructs to truncate the left and right ends of chromosome 14 comprised 4- to 5-kb fragments fused to the nourseothricin *(NAT)* resistance marker and a seed sequence for the telomere. For the truncation of the left end of chromosome 14, a 5-kb region for homologous recombination (HR) was amplified with primers GI008–GI009. A construct of the correct orientation was generated by fusing this fragment with the *NAT* marker amplified with primers GI003–GI013, while for the construct of the opposite orientation *NAT* was amplified with primers GI003 through GI0014. For the truncation of the right end of chromosome 14, a 4-kb region for HR was amplified with primers GI010–GI005. A construct of the correct orientation was generated by fusing this fragment with the *NAT* marker amplified with primers GI003–GI015, while the construct of the opposite orientation and *NAT* was amplified with primers GI003–GI0016. The constructs were used for biolistic transformation of the diploid strain AI187 of *C. neoformans* var. *grubii*. Transformants were selected on YPD+100 µg/mL of nourseothricin, and homologous integration strains were identified by PCR.

### Chromatin immunoprecipitation

ChIP assays were conducted as previously described with some modifications [118], [119]. Briefly, *C. neoformans* was grown in 100 mL YPD until the exponential phase and was crosslinked with 1% formaldehyde at room temperature for 35 min and quenched by adding glycine to a final concentration of 125 mM. The cells were harvested and resuspended in 10 mL of distilled water containing 0.5 mL β-mercaptoethanol and incubated for 1 hour in a shaker incubator at 150 rpm at 30°C. Cells were washed and resuspended in spheroplasting buffer (1 M sorbitol/0.1 M sodium citrate, pH 5.8, and 0.01 M EDTA, pH 8.0) with 40 mg of lysing enzyme from *Trichoderma harzianum* (Sigma) and incubated for 4–5 hours at 37°C. After achieving 90% spheroplasts, the cells were washed as previously described [118], and chromatin was finally resuspended in 1 mL extraction buffer (50 mM HEPES, pH 7.5/140 mM NaCl/1 mM EDTA/0.1% Na-deoxycholate/1% Triton-X) containing protease inhibitor cocktail (Sigma). The lysates were sonicated to obtain chromatin fragments of an average size of 300–500 bp (14× bursts at 30% amplitude with 10 sec pulse using a SONICS Vibra cell). After centrifuging (13,000 rpm, 10 min, 4°C), chromatin was divided to obtain total and IP DNA (with or without antibodies) preparations.

*Total DNA (T)*: Approximately 100 µL of lysate were added to 0.4 mL of elution buffer (1% SDS/0.1M NaHCO_3_) with 20 µl of 5M NaCl. The reaction was incubated at 65°C overnight to reverse the crosslinking. DNA was extracted as described previously [119] and resuspended in 25 µL of MilliQ water containing RNase (10 µg/mL).

*Immunoprecipitated material (IP)*: The remaining lysate (900 µL) was distributed into two 1.5-mL Eppendorf tubes (0.45 mL in each). In one of the tubes, 20 µL of RFP-TRAP beads (ChromoTek) were added and used as IP DNA with antibodies. In another tube, 20 µL of control beads were added to serve as a negative control. Both tubes were incubated overnight at 4°C on a roller. The IP materials were processed as described previously with some modifications [119]. The washing step with high salt buffer was done twice, while the LiCl buffer washing was done only once, and beads were pelleted at 5,400 rpm for two minutes. The isolated DNA was then dried and the pellet was resuspended in 20 µL MilliQ water containing RNase (10 µg/ml). The ChIP sequencing analysis was done as previously described [55]. Briefly, ChIP-Seq analysis was performed at Genotypic Technology. In total, 6 million single-end 36-nt reads for IP and 24 million reads for input DNA were generated on the Illumina GAIIx platform. Raw reads were processed using SeqQC (version 2.2). Reads were aligned to the target *C. neoformans* (*C. neoformans* GCA_000149245.2 with new chromosome 14 assembly) using Bowtie version 0.12.8 and the parameters “-v 3 –best -m 1”. About 90% of the aligned reads were obtained per sample. Peak calling was performed using Homer v3.13 in “histone” mode using default parameters and fold changes of 1.5 and 3. Chromosome-wise read distribution and read depth graphs were generated using R scripts (proprietary to Genotypic Technology, Bangalore, India).

### Analysis of replication intermediates

Cells were grown to mid-log phase in YEPD (2–3×10^7^ cells/mL), mixed with 0.5 volumes of ice-cold Azide stop buffer (0.5 M NaOH, 0.4 M Na_2_EDTA, 2% w/v NaN_3_), collected by filtration through a Nylon filter, and resuspended in cold sterile distilled H_2_O. DNA was prepared from nuclei as described [120]. After digestion with restriction enzymes as indicated, DNA was electrophoresed on neutral-neutral 2D gels, blotted, and hybridized as described [121].

### Statistical analysis for progeny of crosses

Multiple linear regression was used to fit each of the continuous response variables (level of melanization on niger seed and l-DOPA agar) on the basis of all the binary SNP and indel marker values and chromosome 9 genotype. The isolates were treated as a random sample from the *Cryptococcus* population. Multinomial logistic regression was used to predict mating phenotype, categorized as either no mating, resembling H99C or resembling KN99**a**. Further analysis was conducted by collapsing the mating phenotype categories into the following: no mating or like H99C (category 0) or like KN99**a** (category 1). This was considered reasonable because the H99C strain mates much less frequently than the KN99**a** strain. A Bonferroni correction was applied to keep the family-wise error rate at 0.05. Stata (StataCorp LP, College Station, TX) was used for the statistical analysis.

### Stress sensitivity tests

Each H99 passaged strain was incubated overnight (about 16 h) at 30°C in liquid YPD, washed, serially diluted (1 to 10^4^ dilutions) with dH_2_O, and spotted (3 µL) onto solid YPD containing the indicated concentration of stress inducers, such as SDS, CdSO_4_, or fludioxonil. To test oxidative stress, cells were spotted onto solid YPD containing the indicated concentration of *tert*-butyl hydroperoxide (tBOOH), menadione, and diamide. To examine antifungal drug resistance, amphotericin B (AMB), flucytosine (5-FC), and azole drugs, including itraconazole (ICZ), ketoconazole (KCZ), and fluconazole (FCZ), were used. To evaluate ER stress, cells were spotted onto solid YPD containing the indicated concentration of ER stress inducers, such as tunicamycin (TM) or dithiothreitol (DTT). Cells were incubated at 30°C and photographed during the incubation period.

### Urease test

Each strain was cultured overnight (about 16 h) at 30°C in liquid YPD and resuspended in dH_2_O. Equal numbers of *Cryptococcus* cells (10^8^ cells/mL) were spotted (5 µL) onto Christensen's urea agar [122] and incubated at 30°C for two to five days. Each plate was photographed during the incubation period.

### Western blot analysis of Hog1 phosphorylation

Each H99 strain was grown to mid-logarithmic phase in YPD at 30°C. Cultures were resuspended in lysis buffer (50 mM Tris-HCl pH 7.5, 1% sodium deoxycholate, 5 mM sodium pyrophosphate, 10 nM sodium orthovanadate, 50 mM NaF, 0.1% [w/v] SDS, and 1% [v/v] Triton X-100) containing 1× protease inhibitor cocktail (Calbiochem) with 0.5 mm zirconia/silica beads (BioSpec Products, Inc.) and disrupted. Protein concentrations were determined using Pierce BCA Protein Assay Kit (Thermo Scientific), and equal amounts of protein were loaded into a 10% Tris-glycine gel (Novex) and transferred to Immuno-blot PVDF membrane (Bio-Rad). A rabbit p38-MAPK-specific antibody (Cell Signaling Technology) was used to detect of phosphorylated Hog1. A rabbit polyclonal anti-Hog1 antibody (Santa Cruz Biotechnology) was used as a loading control.

### Virulence assays

#### Rabbit virulence assays

Briefly, cryptococcal strains were prepared by growth at 30°C for 2 days in YPD broth. The cells were centrifuged and washed with endotoxin-free phosphate buffered saline (PBS). 10^8^ yeast cells in a volume of 0.3 mL were inoculated intracisternally into 2–3 kg immunosuppressed New Zealand White rabbits (3 rabbits per strain) that had been first sedated with ketamine/xylazine [123]. Rabbits were sedated on days 2, 4, 7 and 10 after inoculation and cerebrospinal fluid was withdrawn, diluted in PBS and plated on YPD agar to assess for quantitative yeast counts. To induce and maintain immunosuppression, rabbits were given an intramuscular injection of a hydrocortisone acetate suspension (5 mg/kg/d) one day prior to inoculation of the yeast cells and daily during infection.

#### Murine virulence assays

Strains of *C. neoformans* were grown overnight in YPD broth. The cells were centrifuged and washed with PBS. Virulence studies were performed using a murine nasal inhalation model of infection. Eight week old CBA/J female mice were inoculated by dripping 0.05 mL of PBS containing the *C. neoformans* cells into the nares of anesthetized mice suspended by their incisors [124]. Mice were monitored daily and those showing the signs of being morbidity (weight loss of greater than 25% or extension of the cerebral portion of the cranium) were sacrificed by CO_2_ asphyxiation.

#### *G. mellonella* virulence assays

For virulence in the wax moth assay, each *G. mellonella* larva was injected in the terminal pseudopod with *C. neoformans* cells (1×10^5^ in 5 µL PBS). Larvae were incubated at 30°C, and virulence was measured by scoring the survival of the larvae every 24 h as previously described [125].

## Supporting Information

Figure S1 *Cryptococcus* protein conservation. A. Conserved protein counts *for C. neoformans* var. *grubii* (H99), *C. neoformans* var. *neoformans* (JEC21), and *C. gattii* (WM276). Counts of proteins in conserved gene clusters, as defined by OrthoMCL [127], are listed in overlapping regions of the Venn diagram. Counts for proteins (including orthologs and paralogs) in individual species (H99, JEC21, and WM276 are shown in red, blue, and green respectively) and the total number of conserved clusters (bold black type) are shown. B. Protein identity of single copy orthologs. OrthoMCL protein clusters with one ortholog per species were aligned with MUSCLE [128] and pairwise identity was computed for each species pair.
(PDF)

Figure S2 A. Relationship between the distance between sites within a cluster and the number of poly(A) clusters. B. Distance between the poly(A) clusters within a single mRNA.
(PPT)

Figure S3 Additional examples of differential expression of miscRNAs antisense of a coding gene as observed by Northern blot. RNA was extracted from cells growing in YPD (2×10^8^ cells/mL) at 30°C (condition 1), YPD (5×10^7^ cells/mL) at 30°C (condition 2), YPD with 0.01% SDS (5×10^7^ cells/mL) at 30°C (condition 3), YPD with 10 mg/mL fluconazole (5×10^7^ cells/mL) at 30°C (condition 4), YPD (5×10^7^ cells/mL) at 37°C (condition 5), and YP galactose (2×10^8^ cells/mL) at 30°C (condition 6) in duplicate. Then, 5 µg were separated on a denaturing electrophoresis agarose gel, electrophoresed, and transferred to a nylon membrane. RNAs were then hybridized with strand-specific probes. Black lanes represent the positions of probes. Schematics of the genome loci organizations are given.
(PPT)

Figure S4 RNA-Seq analysis of centromeric regions. Low transcript levels are observed between the last genes bordering the centromeric regions in each chromosome. The coordinates indicate the position of the part of the chromosome visualized through Artemis.
(PPT)

Figure S5 Plasmid replication intermediates analysis of two *C. neoformans* plasmids (pPM8 and pCSN5) shows that linear plasmids cannot be used to identify *bona fide* replication origins in *Cryptococcus*. (Left upper panel) The 2D gel patterns of overlapping fragments of pPM8, which show strong arcs of Y-shaped intermediates and weaker complete replication bubble arcs, indicate that replication initiates throughout the linear plasmid, although the bubble signal is more intense in the right part of the molecule containing *URA5*. (Right upper panel) The 2D gel patterns of pCSN5 replication intermediates show a strong arc of Y-shaped molecules and a weaker pattern of replication termination intermediates, which are replicated by converging forks, indicating that replication initiates at or near the telomeres of the plasmid. (Lower panels) 2D gel patterns of the 3,858-bp *Stu*I and the 3,127-bp *Msc*I fragments from the chromosomal region containing *URA5*, diagrammed below. Restriction fragments of this region contain only Y-shaped replication intermediates, indicating that replication does not initiate at detectable levels within the *URA5* locus on the chromosome. The arcs containing bubble-shaped (B), Y-shaped (Y), and termination (T) replication intermediates are labeled on the 2D gel pattern. The red arrows at the ends of the plasmid molecules represent telomeres.
(PPT)

Figure S6 Phenotypic variations in response to environmental cues and antifungal drug resistance among different H99 passage strains. (A–F) Each *C. neoformans* strain (H99O, H99F, H99S, H99W, H99E, KN99α, KN99a, and H99C) was incubated overnight (about 16 h) at 30°C in liquid YPD medium, washed, serially diluted (1 to 10^4^ dilutions) with dH_2_O, and spotted (3 µL) onto solid YPD containing the indicated concentration of stress inducers or antifungal drugs (0.5 mM tBOOH; 0.02 mM menadione; 2.5 mM diamide; 0.2 µM CdSO_4_; 0.03% SDS; 0.3 µg/mL TM; 20 mM DTT; 0.04 µg/mL ICZ; 0.2 µg/mL KCZ; 13 µg/mL FCZ; 1.1 µg/mL AMB; 800 µg/mL 5-FC; and 1.5 µg/mL fludioxonil). (G) Different H99 passaged strains were cultured to mid-logarithmic phase in YPD at 30°C, and total protein extracts were prepared for western blot analysis as described in the Materials and Methods. To examine Hog1 phosphorylation levels, a rabbit antibody specific to dually phosphorylated p38-MAPK was used. The same blot was stripped and then probed with polyclonal anti-Hog1 antibody as a loading control.
(PPT)

Figure S7 Urease production in different H99 passaged strains. Each *C. neoformans* strain (H99O, H99F, H99S, H99W, H99E, KN99α, KN99**a**, and H99C) was cultured overnight (about 16 h) at 30°C in liquid YPD and resuspended with dH_2_O. Then, 5 µL of a suspension containing 10^8^ cells/mL were spotted onto solid urea-containing agar (Christensen's medium) and incubated at 30°C for two to five days. Urea is a nitrogen source and is converted to ammonia by urease secreted in *C. neoformans*, which increases the pH of the medium. An increased pH is indicated by a change in color from yellow to red-violet color due to the inclusion of phenol red, a pH indicator. Each plate was photographed during the incubation period.
(PPT)

Figure S8 Electrophoretic karyotypic analysis via PFGE of the H99 strains revealed a size reduction of chromosome 9 in H99ED and H99C. Probing of the left and right telomeres following in-gel digestion with *Swa*I and *Sfi*I of chromosomal plugs revealed that while the left subtelomere fragments of chromosome 9 were identical in length for all eight strains tested, the right subtelomere of H99ED and H99C was ∼25 kb smaller (approximate position marked with “?”). The *Swa*I-digested blot was hybridized to the chromosome 9L probe (yellow arrow) while the *Sfi*I-digested blot was hybridized to the chromosome 9R probe (green arrow). The size of the band in reference to the band size of the laboratory reference strain H99O indicates whether any telomeric length changes have taken place.
(PPT)

Figure S9 Sequencing the end of the subtelomere of chromosome 9R in H99ED. The new chromosome endpoint in these strains was characterized via PCR to determine the precise nucleotide at which they were truncated, confirming the loss of a region containing nine genes, all hypothetical proteins (CNAG_07002, CNAG_07786, CNAG_07787, CNAG_07788, CNAG_06953, CNAG_06954, CNAG_07789, CNAG_07790, CNAG_07791). Importantly, while it was confirmed that the segment was deleted, all of these genes have duplicates elsewhere in the genome, as is the case with most *C. neoformans* subtelomeric genes. Strain H99O was used as a negative control. The PCR product obtained in the UQ1261/UQ618 reaction was sequenced and aligned against the H99O sequence.
(PPT)

Figure S10 Phenotypic analysis of F1 progenies. A. Mating phenotype segregates in progeny set. Mating assays with KN99**a** (H99C and progeny 1, 3, 7, 9, 10, 13, 14, 18, 20, 23, and 27) and KN99α (KN99**a** and progeny 2, 4, 5, 6, 8, 11, 12, 15, 16, 17, 19, 21, 22, 24, 25, and 26) on V8 agar incubated at room temperature for seven days in the dark. B. Melanin phenotype segregates in progeny set. Melanization assays on (left) l–DOPA agar or, (right) niger seed agar incubated at 37°C for two to three days.
(PPT)

Table S1 List of the modifications of the *C. neoformans* genome annotation.
(DOC)

Table S2 Compared sequence similarities between the new and the former protein set and the protein set of *S. cerevisiae*.
(XLS)

Table S3 List of protein families amplified in the *Cryptococcus* lineage.
(XLS)

Table S4 Genes expressed without an intron in *C. neoformans* var. *grubii*.
(DOC)

Table S5 List of the genes with overlapping CDS.
(XLS)

Table S6 Coordinates of the centromeric regions in *C. neoformans* H99.
(DOC)

Table S7 Positions of the replication origin in *C. neoformans*.
(DOC)

Table S8 *Cryptococcus* orthologs of DNA replication initiation proteins.
(DOC)

Table S9 SNPs and indels identified in H99 series.
(DOC)

Table S10 Sequencing read statistics.
(DOC)

Table S11 List of the Bioprojects associated with the present study.
(XLS)

Table S12 Primers used in this study.
(DOC)

Text S1 History of the H99 strain and consult note of February 14, 1978.
(DOC)
