# Supplementary figures and images for: Analysis of the Genome and Transcriptome of Cryptococcus neoformans var. grubii Reveals Complex RNA Expression and Microevolution Leading to Virulence Attenuation
Source: PLoS Genet. 2014 Apr 17;10(4):e1004261. doi: 10.1371/journal.pgen.1004261 (PMC3990503; doi:10.1371/journal.pgen.1004261)

**A**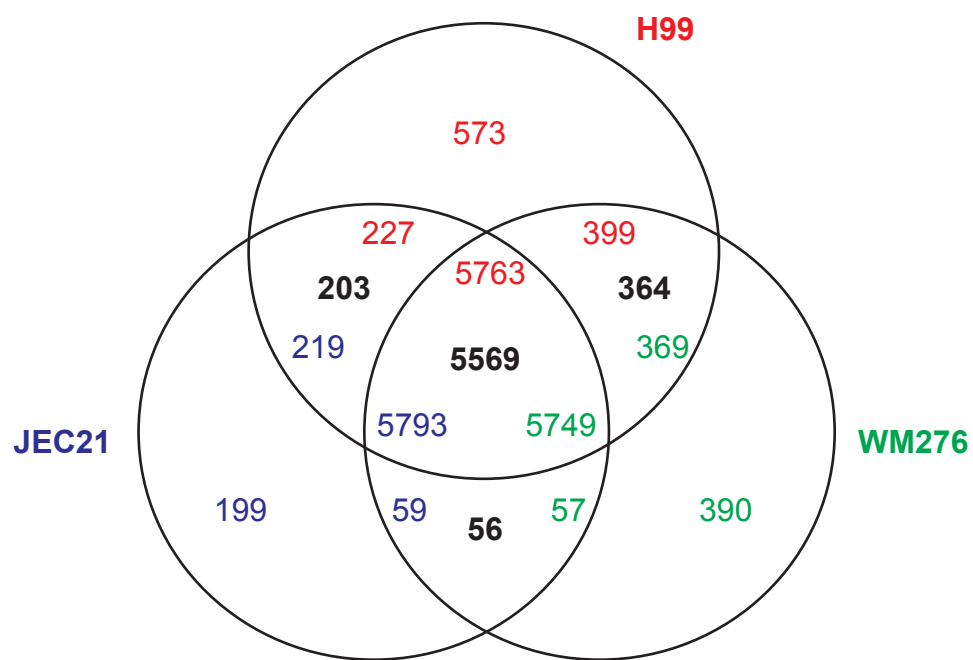**B**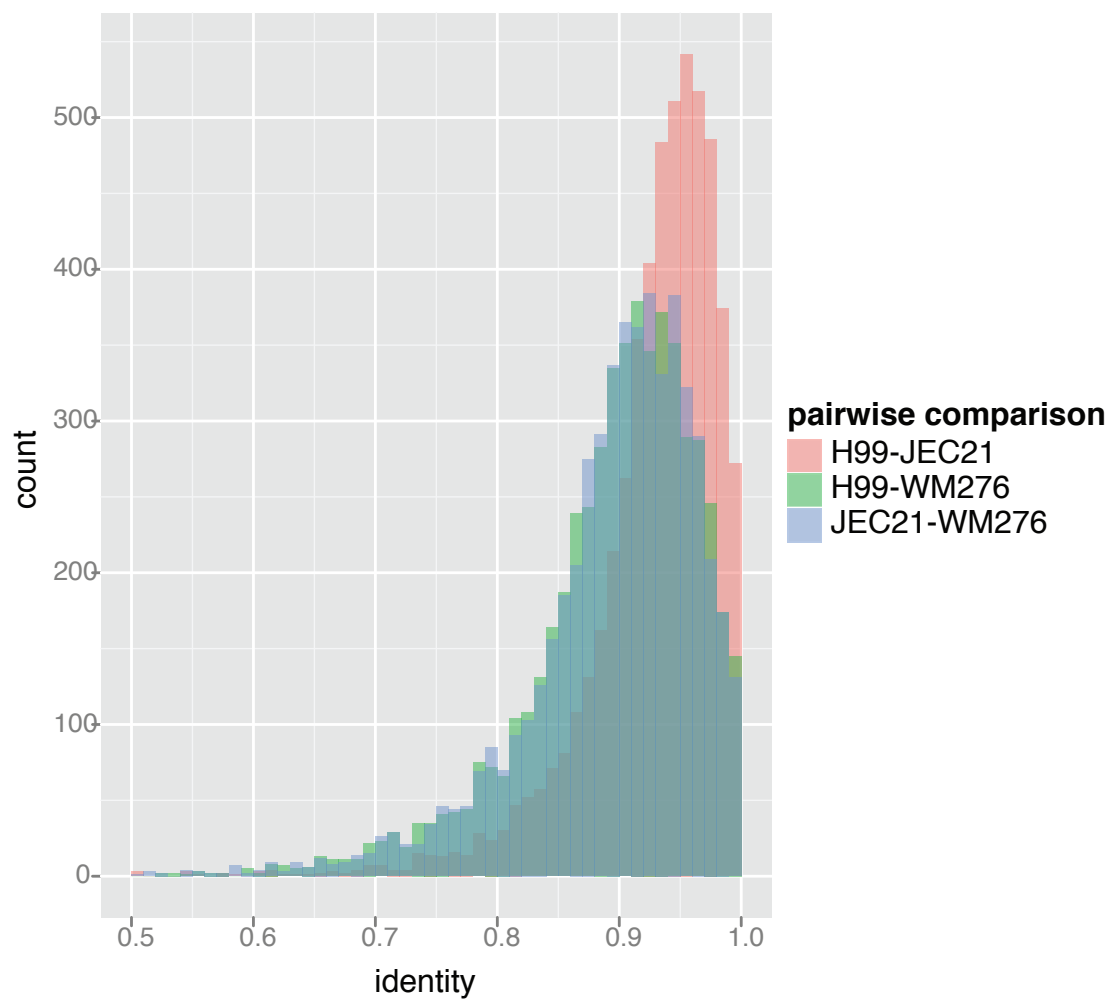

Supplement: Figure S1 — Cryptococcus protein conservation. A. Conserved protein counts for C. neoformans var. grubii (H99), C. neoformans var. neoformans (JEC21), and C. gattii (WM276). Counts of proteins in conserved gene clusters, as defined by OrthoMCL [127], are listed in overlapping regions of the Venn diagram. Counts for proteins (including orthologs and paralogs) in individual species (H99, JEC21, and WM276 are shown in red, blue, and green respectively) and the total number of conserved clusters (bold black type) are shown. B. Protein identity of single copy orthologs. OrthoMCL protein clusters with one ortholog per species were aligned with MUSCLE [128] and pairwise identity was computed for each species pair. (PDF) [file pgen.1004261.s001.pdf]

## Slide 1
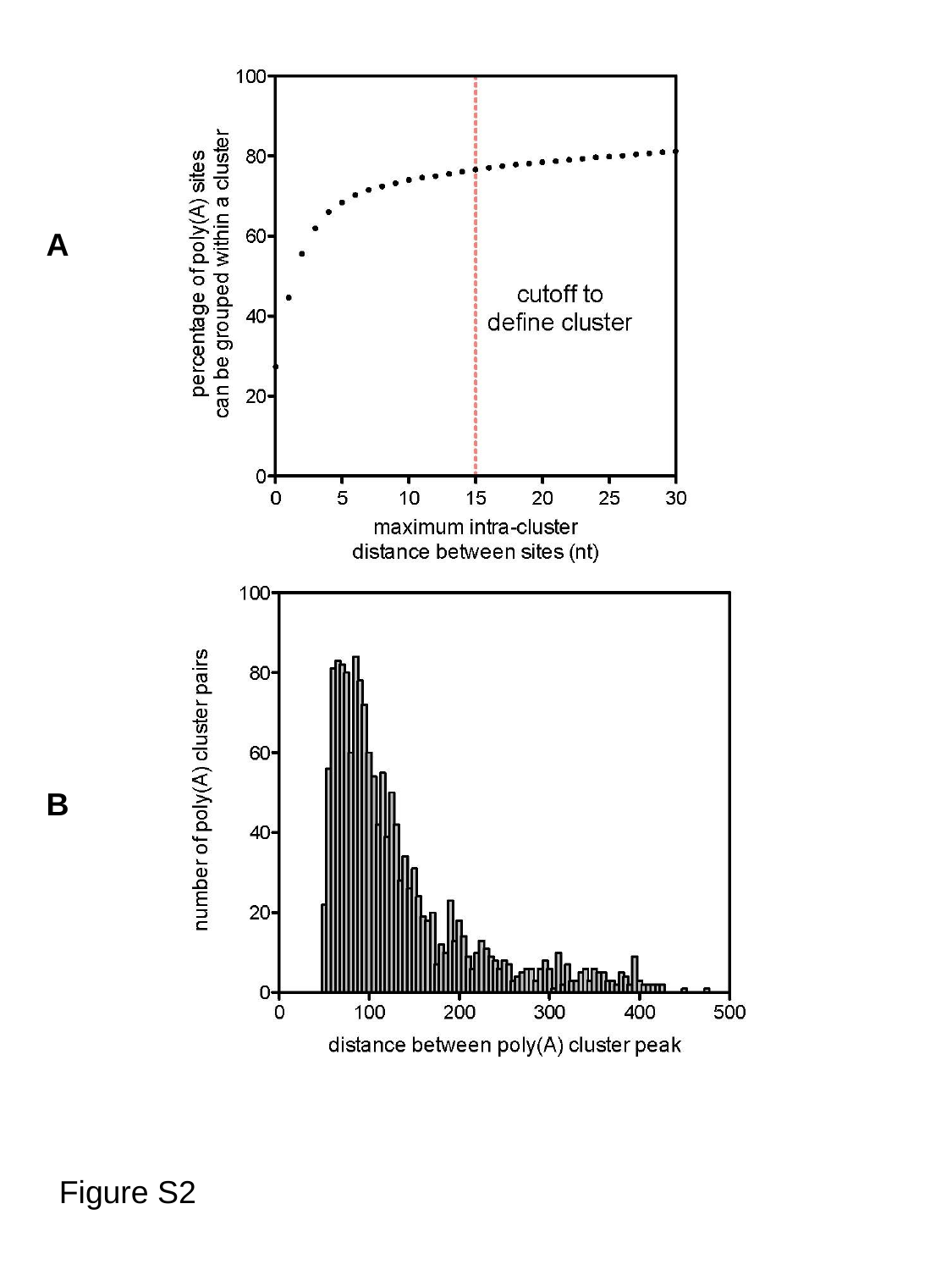

A
B
Figure S2

Supplement: Figure S2 — A. Relationship between the distance between sites within a cluster and the number of poly(A) clusters. B. Distance between the poly(A) clusters within a single mRNA. (PPT) [file pgen.1004261.s002.ppt]

## Slide 1
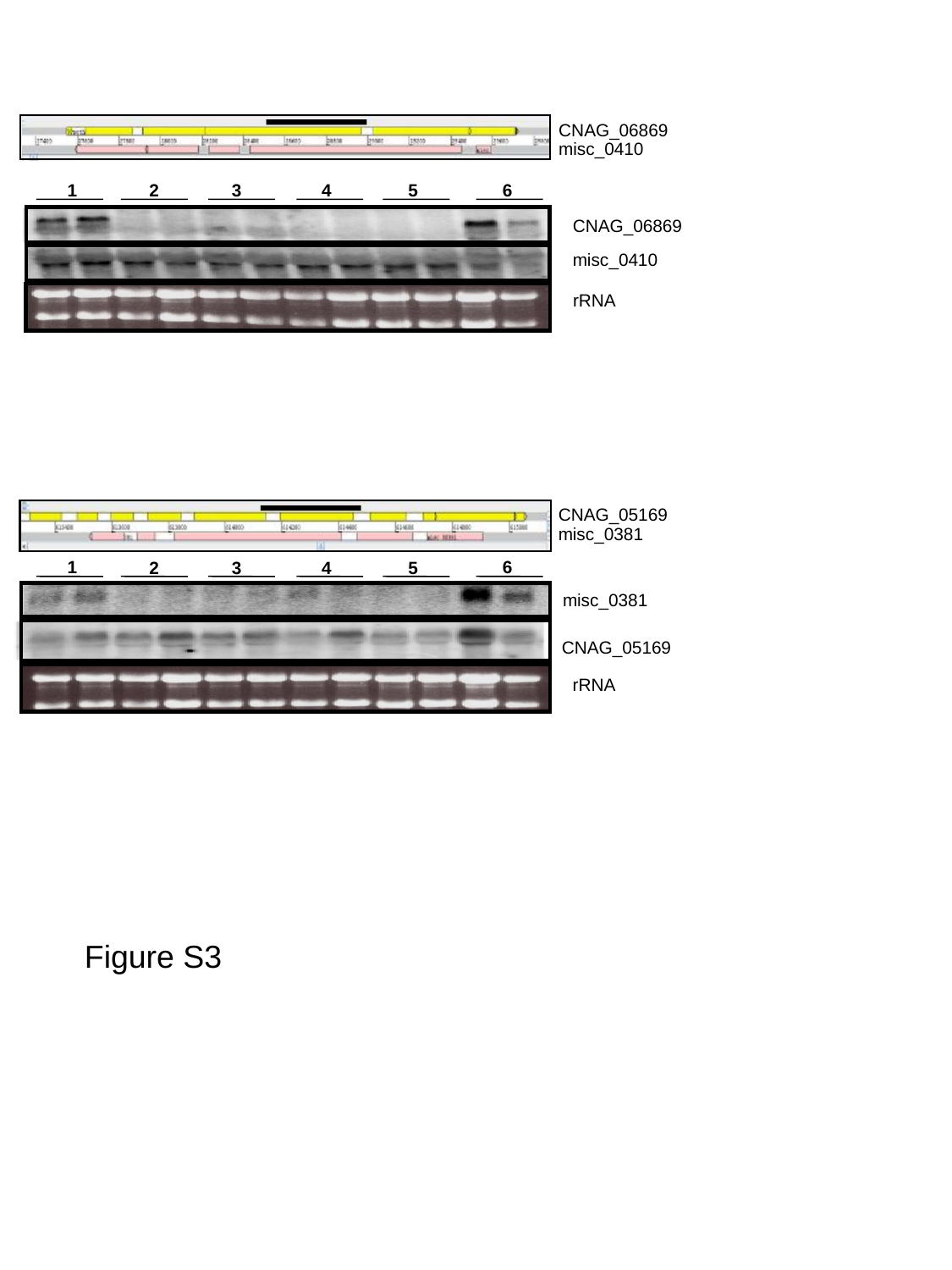

CNAG_06869
misc_0410
1
6
2
3
4
5
CNAG_06869
misc_0410
rRNA
CNAG_05169
misc_0381
1
6
2
3
4
5
misc_0381
CNAG_05169
rRNA
Figure S3

Supplement: Figure S3 — Additional examples of differential expression of miscRNAs antisense of a coding gene as observed by Northern blot. RNA was extracted from cells growing in YPD (2×10^8^ cells/mL) at 30°C (condition 1), YPD (5×10^7^ cells/mL) at 30°C (condition 2), YPD with 0.01% SDS (5×10^7^ cells/mL) at 30°C (condition 3), YPD with 10 mg/mL fluconazole (5×10^7^ cells/mL) at 30°C (condition 4), YPD (5×10^7^ cells/mL) at 37°C (condition 5), and YP galactose (2×10^8^ cells/mL) at 30°C (condition 6) in duplicate. Then, 5 µg were separated on a denaturing electrophoresis agarose gel, electrophoresed, and transferred to a nylon membrane. RNAs were then hybridized with strand-specific probes. Black lanes represent the positions of probes. Schematics of the genome loci organizations are given. (PPT) [file pgen.1004261.s003.ppt]

## Slide 1
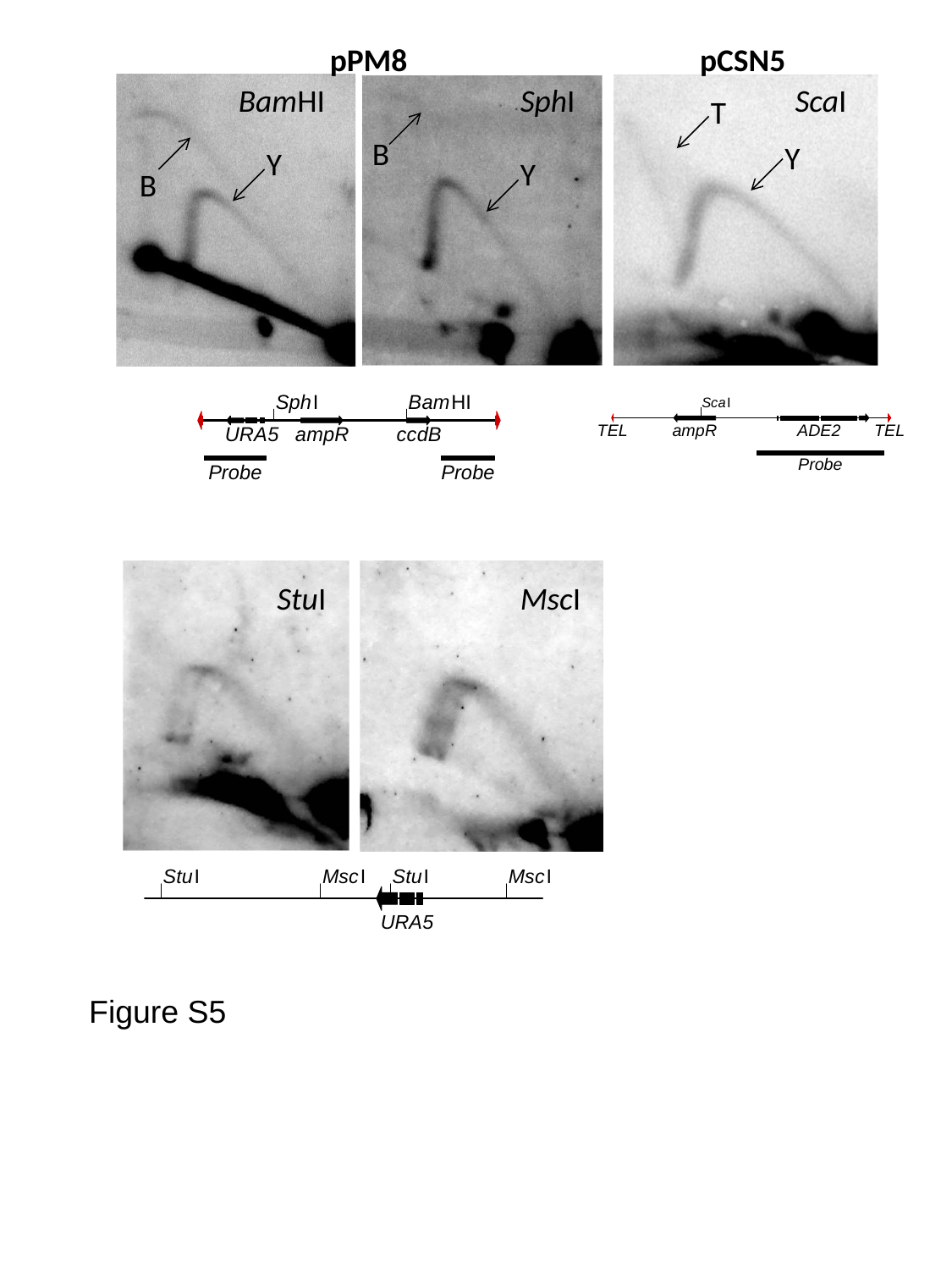

pPM8
pCSN5
BamHI
SphI
ScaI
T
B
Y
Y
Y
B
StuI
MscI
Figure S5

Supplement: Figure S5 — Plasmid replication intermediates analysis of two C. neoformans plasmids (pPM8 and pCSN5) shows that linear plasmids cannot be used to identify bona fide replication origins in Cryptococcus. (Left upper panel) The 2D gel patterns of overlapping fragments of pPM8, which show strong arcs of Y-shaped intermediates and weaker complete replication bubble arcs, indicate that replication initiates throughout the linear plasmid, although the bubble signal is more intense in the right part of the molecule containing URA5. (Right upper panel) The 2D gel patterns of pCSN5 replication intermediates show a strong arc of Y-shaped molecules and a weaker pattern of replication termination intermediates, which are replicated by converging forks, indicating that replication initiates at or near the telomeres of the plasmid. (Lower panels) 2D gel patterns of the 3,858-bp StuI and the 3,127-bp MscI fragments from the chromosomal region containing URA5, diagrammed below. Restriction fragments of this region contain only Y-shaped replication intermediates, indicating that replication does not initiate at detectable levels within the URA5 locus on the chromosome. The arcs containing bubble-shaped (B), Y-shaped (Y), and termination (T) replication intermediates are labeled on the 2D gel pattern. The red arrows at the ends of the plasmid molecules represent telomeres. (PPT) [file pgen.1004261.s005.ppt]

## Slide 1
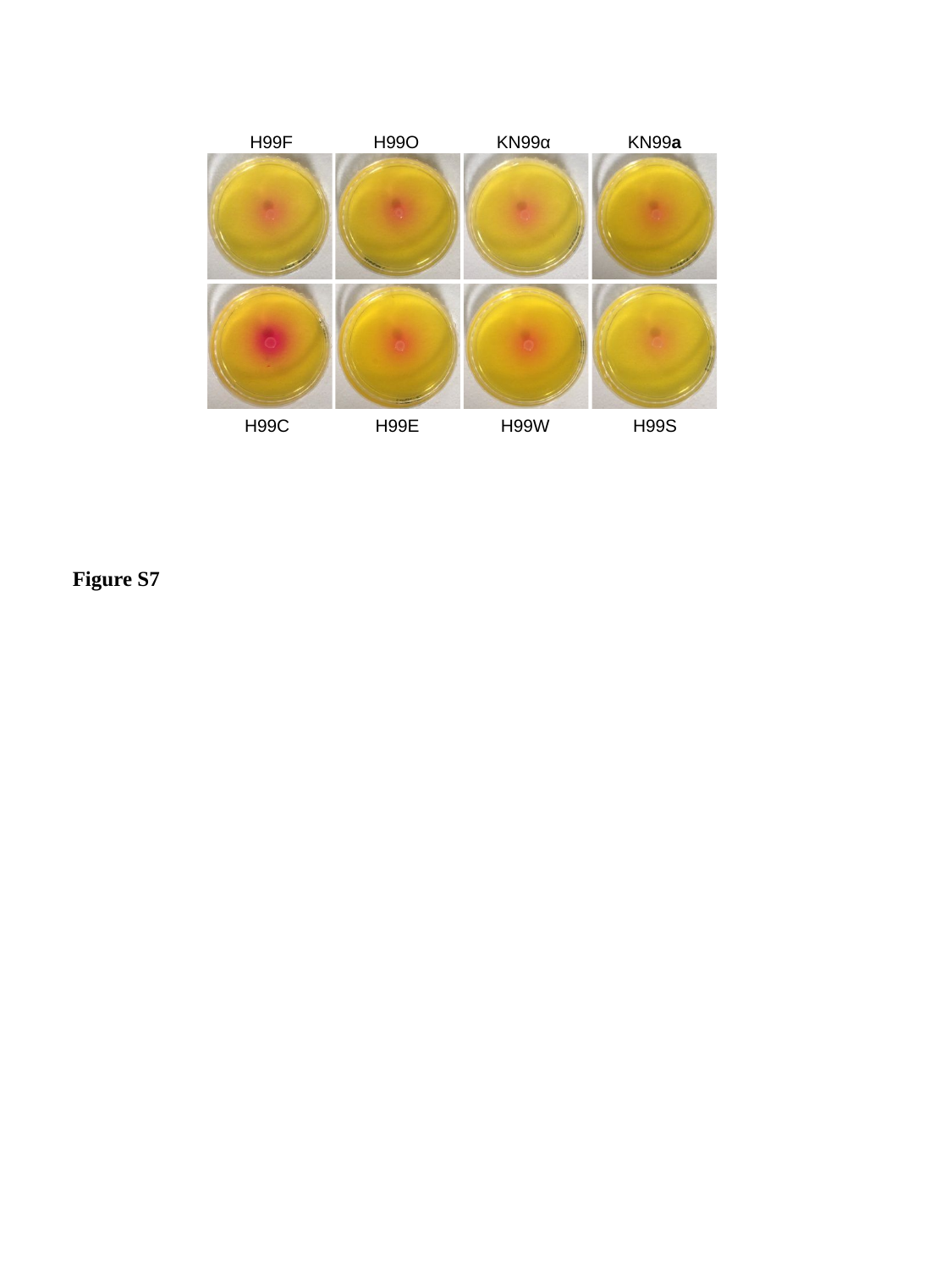

H99F
H99O
KN99α
KN99a
H99S
H99C
H99E
H99W
Figure S7

Supplement: Figure S7 — Urease production in different H99 passaged strains. Each C. neoformans strain (H99O, H99F, H99S, H99W, H99E, KN99α, KN99a, and H99C) was cultured overnight (about 16 h) at 30°C in liquid YPD and resuspended with dH2O. Then, 5 µL of a suspension containing 108 cells/mL were spotted onto solid urea-containing agar (Christensen's medium) and incubated at 30°C for two to five days. Urea is a nitrogen source and is converted to ammonia by urease secreted in C. neoformans, which increases the pH of the medium. An increased pH is indicated by a change in color from yellow to red-violet color due to the inclusion of phenol red, a pH indicator. Each plate was photographed during the incubation period. (PPT) [file pgen.1004261.s007.ppt]

## Slide 1
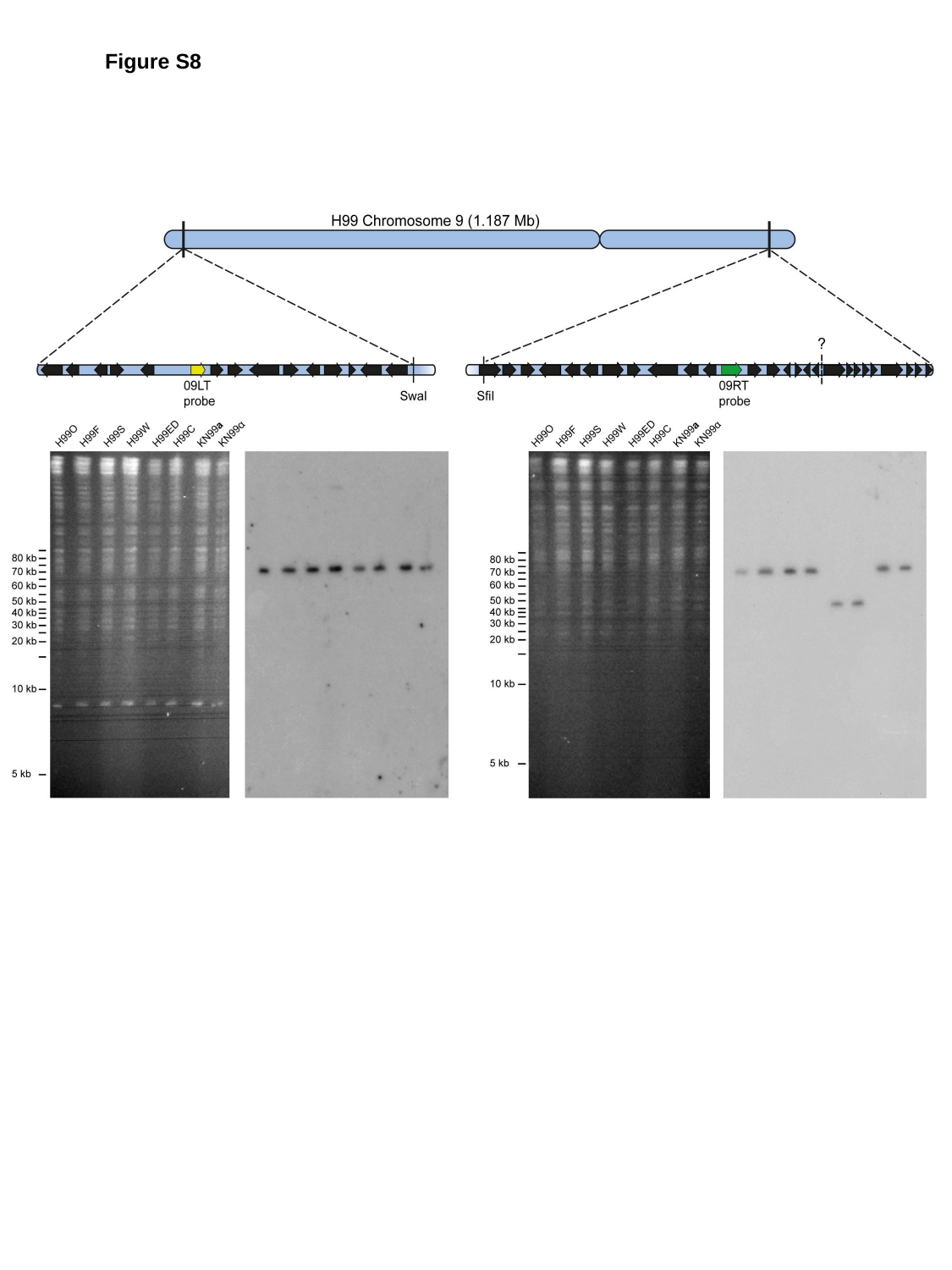

Figure S8

Supplement: Figure S8 — Electrophoretic karyotypic analysis via PFGE of the H99 strains revealed a size reduction of chromosome 9 in H99ED and H99C. Probing of the left and right telomeres following in-gel digestion with SwaI and SfiI of chromosomal plugs revealed that while the left subtelomere fragments of chromosome 9 were identical in length for all eight strains tested, the right subtelomere of H99ED and H99C was ∼25 kb smaller (approximate position marked with “?”). The SwaI-digested blot was hybridized to the chromosome 9L probe (yellow arrow) while the SfiI-digested blot was hybridized to the chromosome 9R probe (green arrow). The size of the band in reference to the band size of the laboratory reference strain H99O indicates whether any telomeric length changes have taken place. (PPT) [file pgen.1004261.s008.ppt]

## Slide 1
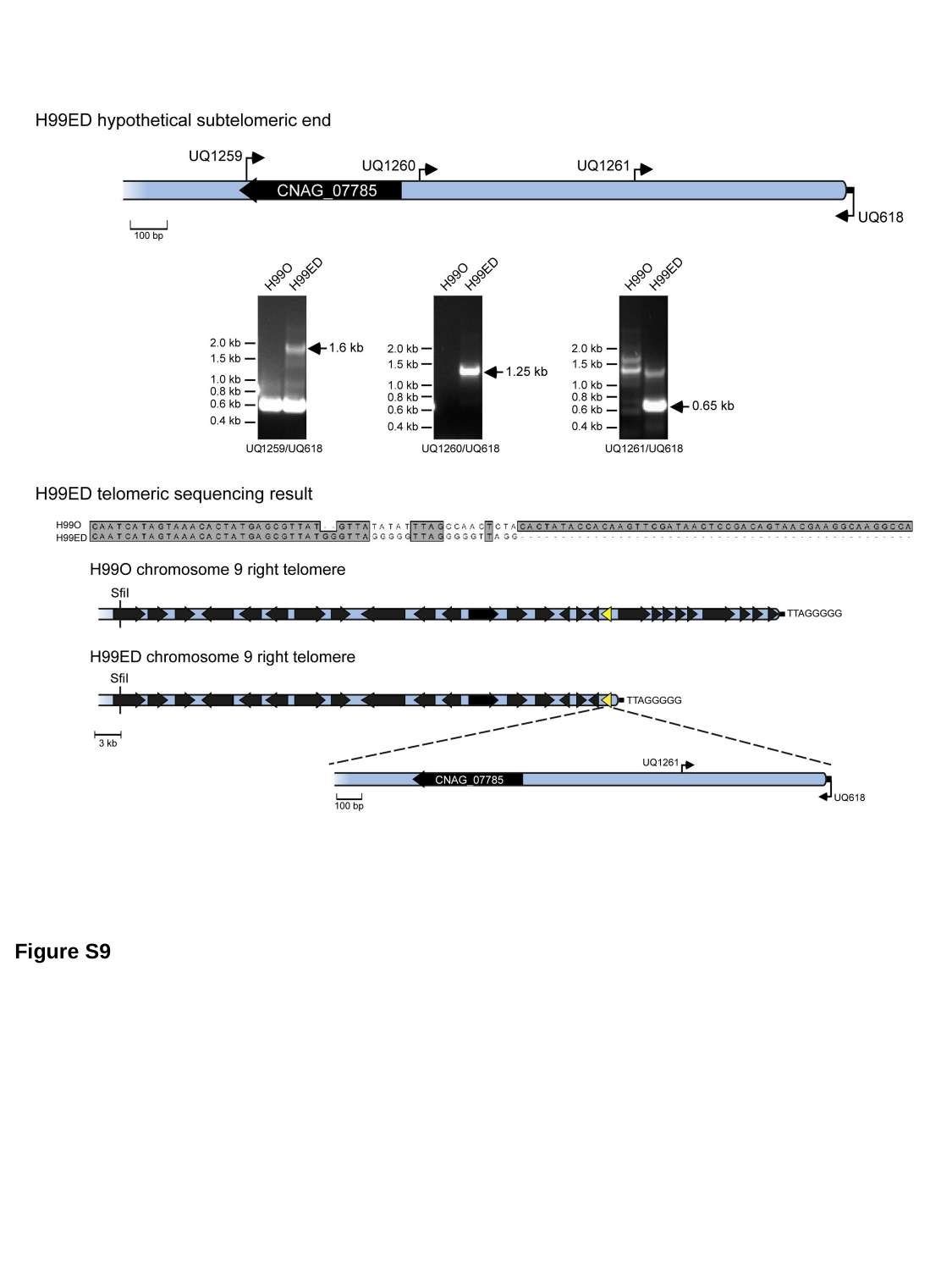

Figure S9

Supplement: Figure S9 — Sequencing the end of the subtelomere of chromosome 9R in H99ED. The new chromosome endpoint in these strains was characterized via PCR to determine the precise nucleotide at which they were truncated, confirming the loss of a region containing nine genes, all hypothetical proteins (CNAG_07002, CNAG_07786, CNAG_07787, CNAG_07788, CNAG_06953, CNAG_06954, CNAG_07789, CNAG_07790, CNAG_07791). Importantly, while it was confirmed that the segment was deleted, all of these genes have duplicates elsewhere in the genome, as is the case with most C. neoformans subtelomeric genes. Strain H99O was used as a negative control. The PCR product obtained in the UQ1261/UQ618 reaction was sequenced and aligned against the H99O sequence. (PPT) [file pgen.1004261.s009.ppt]

## Slide 1
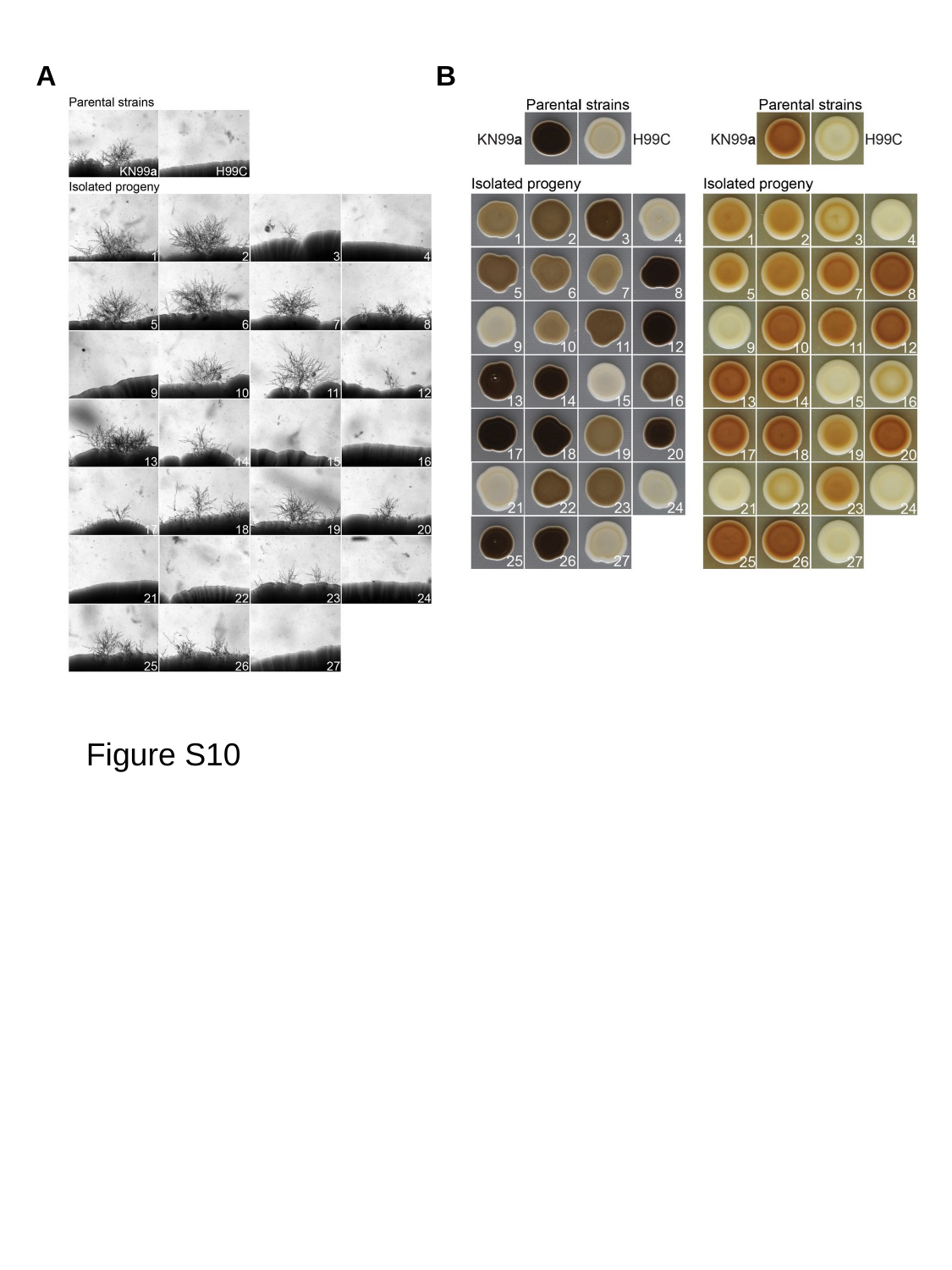

A
B
Figure S10

Supplement: Figure S10 — Phenotypic analysis of F1 progenies. A. Mating phenotype segregates in progeny set. Mating assays with KN99a (H99C and progeny 1, 3, 7, 9, 10, 13, 14, 18, 20, 23, and 27) and KN99α (KN99a and progeny 2, 4, 5, 6, 8, 11, 12, 15, 16, 17, 19, 21, 22, 24, 25, and 26) on V8 agar incubated at room temperature for seven days in the dark. B. Melanin phenotype segregates in progeny set. Melanization assays on (left) l–DOPA agar or, (right) niger seed agar incubated at 37°C for two to three days. (PPT) [file pgen.1004261.s010.ppt]
