# Supplementary material for: Analysis of the Genome and Transcriptome of Cryptococcus neoformans var. grubii Reveals Complex RNA Expression and Microevolution Leading to Virulence Attenuation
Source: PLoS Genet. 2014 Apr 17;10(4):e1004261. doi: 10.1371/journal.pgen.1004261 (PMC3990503; doi:10.1371/journal.pgen.1004261)
Supplement: Figure S4 — RNA-Seq analysis of centromeric regions. Low transcript levels are observed between the last genes bordering the centromeric regions in each chromosome. The coordinates indicate the position of the part of the chromosome visualized through Artemis. (PPT) [file pgen.1004261.s004.ppt]

## Slide 1
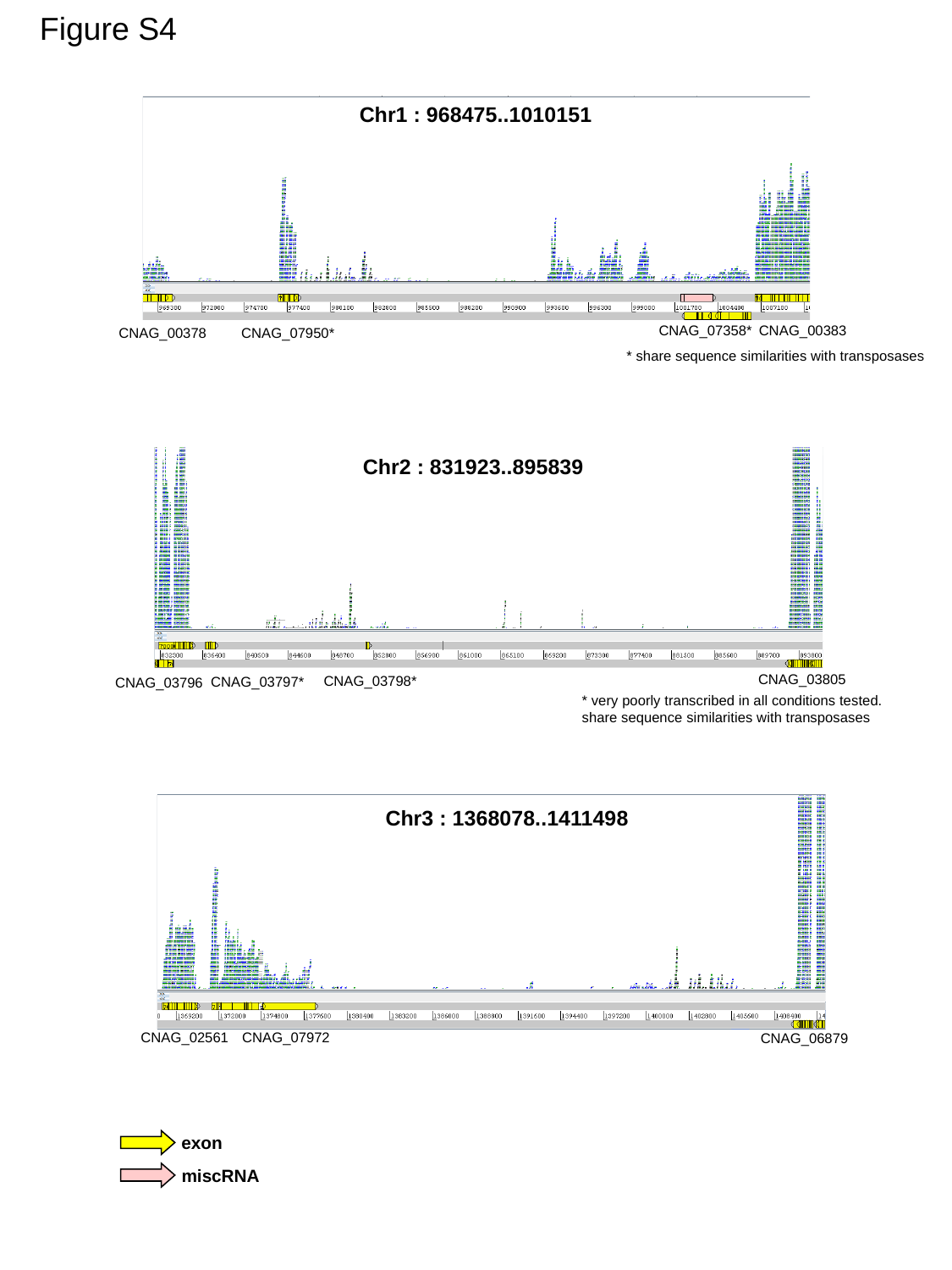

Figure S4
Chr1 : 968475..1010151
CNAG_07358*
CNAG_00383
CNAG_00378
CNAG_07950*
* share sequence similarities with transposases
Chr2 : 831923..895839
CNAG_03805
CNAG_03798*
CNAG_03797*
CNAG_03796
* very poorly transcribed in all conditions tested. share sequence similarities with transposases
Chr3 : 1368078..1411498
CNAG_02561
CNAG_07972
CNAG_06879
exon
miscRNA

## Slide 2
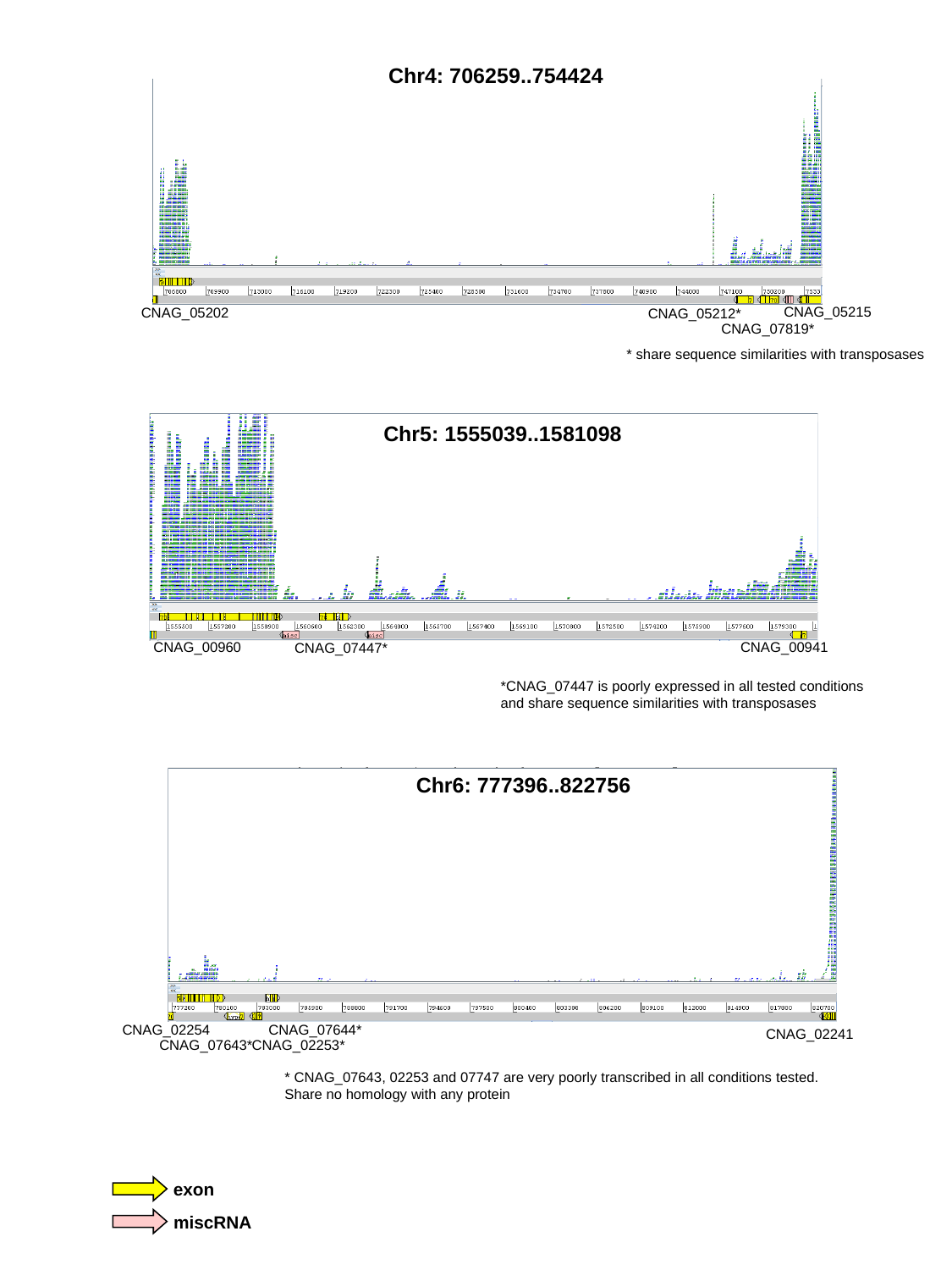

Chr4: 706259..754424
CNAG_05215
CNAG_05202
CNAG_05212*
CNAG_07819*
* share sequence similarities with transposases
Chr5: 1555039..1581098
CNAG_00941
CNAG_00960
CNAG_07447*
*CNAG_07447 is poorly expressed in all tested conditions
and share sequence similarities with transposases
Chr6: 777396..822756
CNAG_02254
CNAG_07644*
CNAG_02241
CNAG_07643*
CNAG_02253*
* CNAG_07643, 02253 and 07747 are very poorly transcribed in all conditions tested.
Share no homology with any protein
exon
miscRNA

## Slide 3
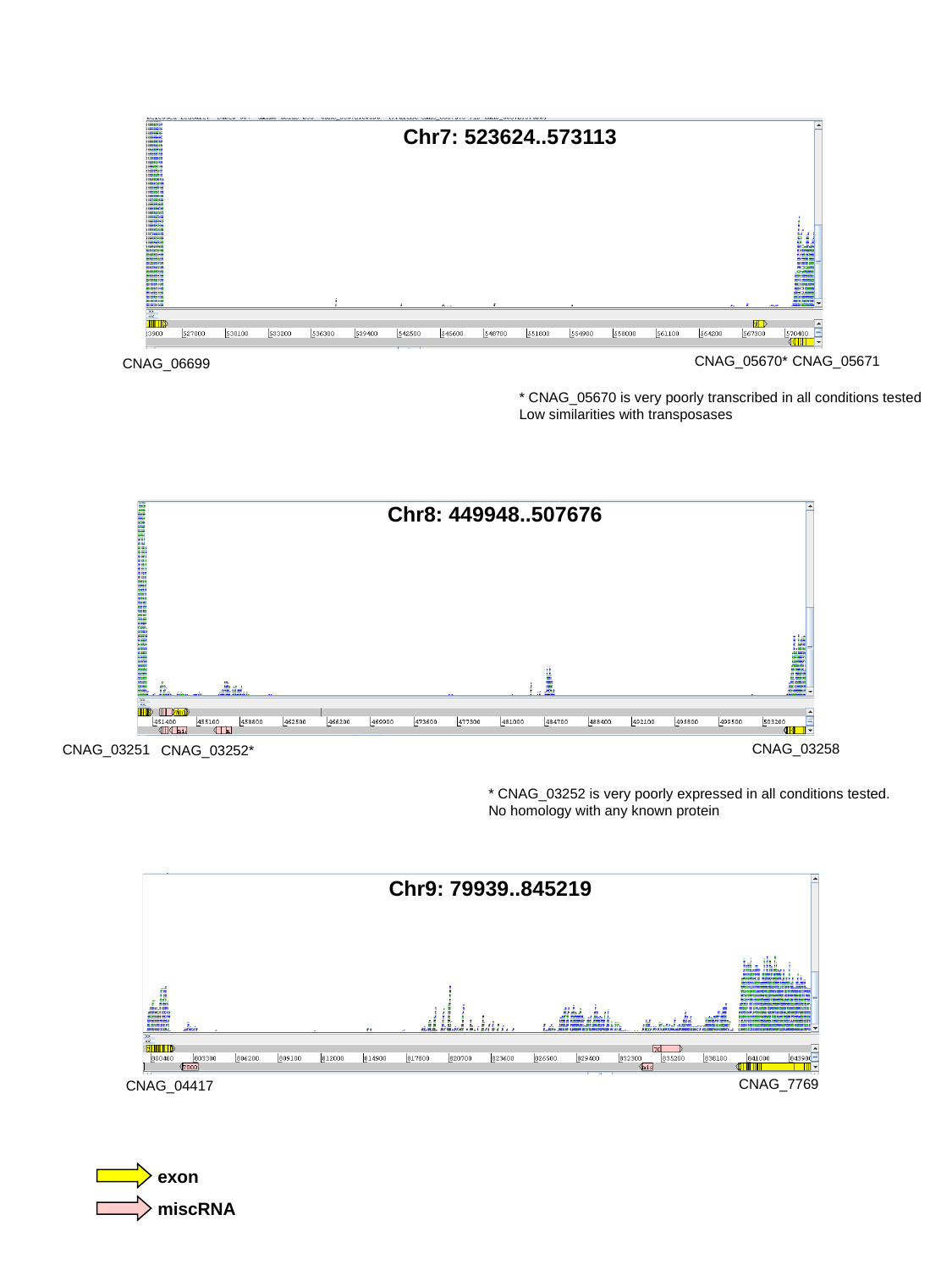

Chr7: 523624..573113
CNAG_05670*
CNAG_05671
CNAG_06699
* CNAG_05670 is very poorly transcribed in all conditions tested
Low similarities with transposases
Chr8: 449948..507676
CNAG_03258
CNAG_03251
CNAG_03252*
* CNAG_03252 is very poorly expressed in all conditions tested. No homology with any known protein
Chr9: 79939..845219
CNAG_7769
CNAG_04417
exon
miscRNA

## Slide 4
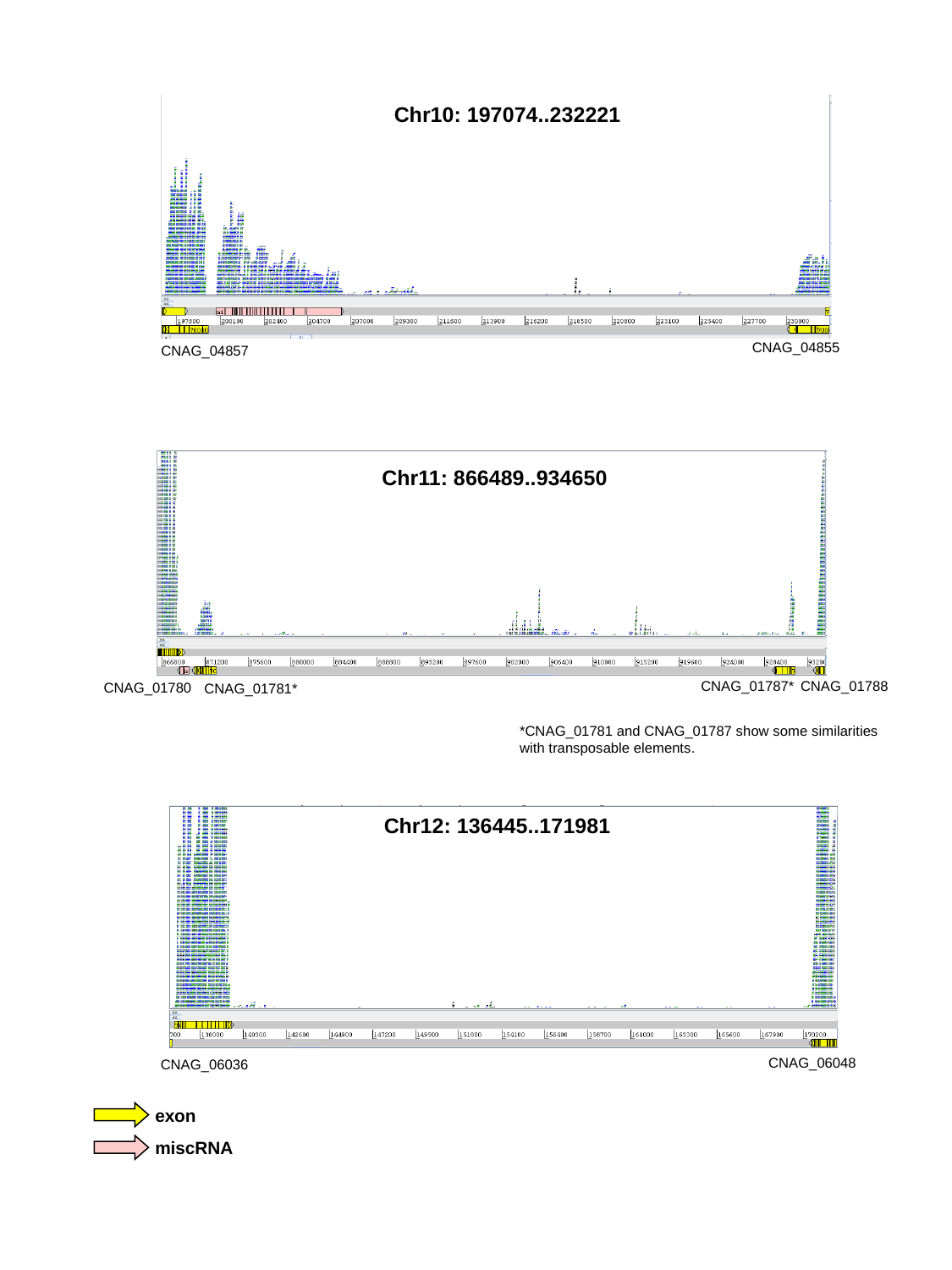

Chr10: 197074..232221
CNAG_04855
CNAG_04857
Chr11: 866489..934650
CNAG_01787*
CNAG_01788
CNAG_01780
CNAG_01781*
*CNAG_01781 and CNAG_01787 show some similarities with transposable elements.
Chr12: 136445..171981
CNAG_06048
CNAG_06036
exon
miscRNA

## Slide 5
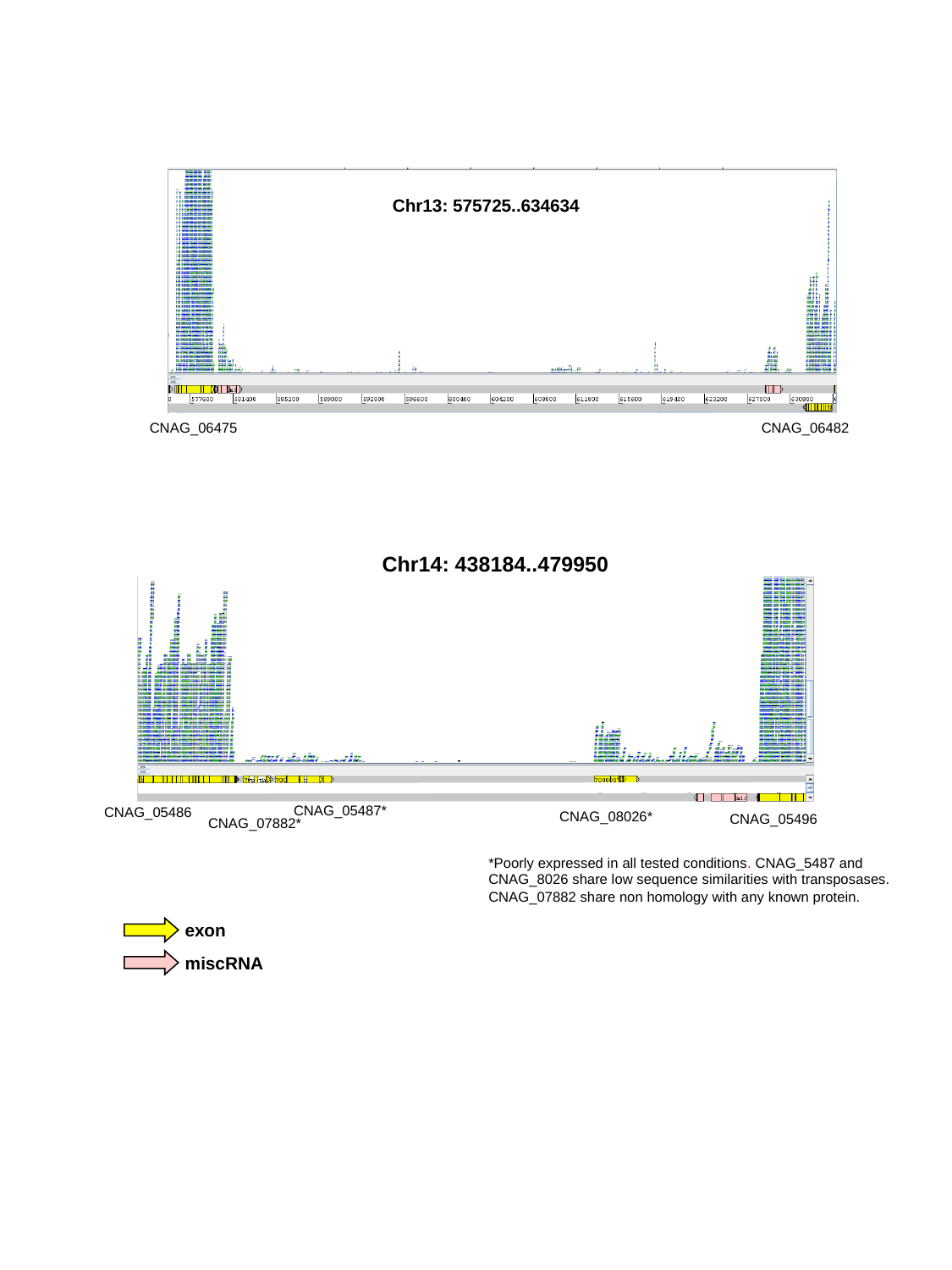

Chr13: 575725..634634
CNAG_06482
CNAG_06475
Chr14: 438184..479950
CNAG_05487*
CNAG_05486
CNAG_08026*
CNAG_05496
CNAG_07882*
*Poorly expressed in all tested conditions. CNAG_5487 and CNAG_8026 share low sequence similarities with transposases. CNAG_07882 share non homology with any known protein.
exon
miscRNA
