# Supplementary material for: Analysis of the Genome and Transcriptome of Cryptococcus neoformans var. grubii Reveals Complex RNA Expression and Microevolution Leading to Virulence Attenuation
Source: PLoS Genet. 2014 Apr 17;10(4):e1004261. doi: 10.1371/journal.pgen.1004261 (PMC3990503; doi:10.1371/journal.pgen.1004261)
Supplement: Figure S6 — Phenotypic variations in response to environmental cues and antifungal drug resistance among different H99 passage strains. (A–F) Each C. neoformans strain (H99O, H99F, H99S, H99W, H99E, KN99α, KN99a, and H99C) was incubated overnight (about 16 h) at 30°C in liquid YPD medium, washed, serially diluted (1 to 104 dilutions) with dH2O, and spotted (3 µL) onto solid YPD containing the indicated concentration of stress inducers or antifungal drugs (0.5 mM tBOOH; 0.02 mM menadione; 2.5 mM diamide; 0.2 µM CdSO4; 0.03% SDS; 0.3 µg/mL TM; 20 mM DTT; 0.04 µg/mL ICZ; 0.2 µg/mL KCZ; 13 µg/mL FCZ; 1.1 µg/mL AMB; 800 µg/mL 5-FC; and 1.5 µg/mL fludioxonil). (G) Different H99 passaged strains were cultured to mid-logarithmic phase in YPD at 30°C, and total protein extracts were prepared for western blot analysis as described in the Materials and Methods. To examine Hog1 phosphorylation levels, a rabbit antibody specific to dually phosphorylated p38-MAPK was used. The same blot was stripped and then probed with polyclonal anti-Hog1 antibody as a loading control. (PPT) [file pgen.1004261.s006.ppt]

## Slide 1
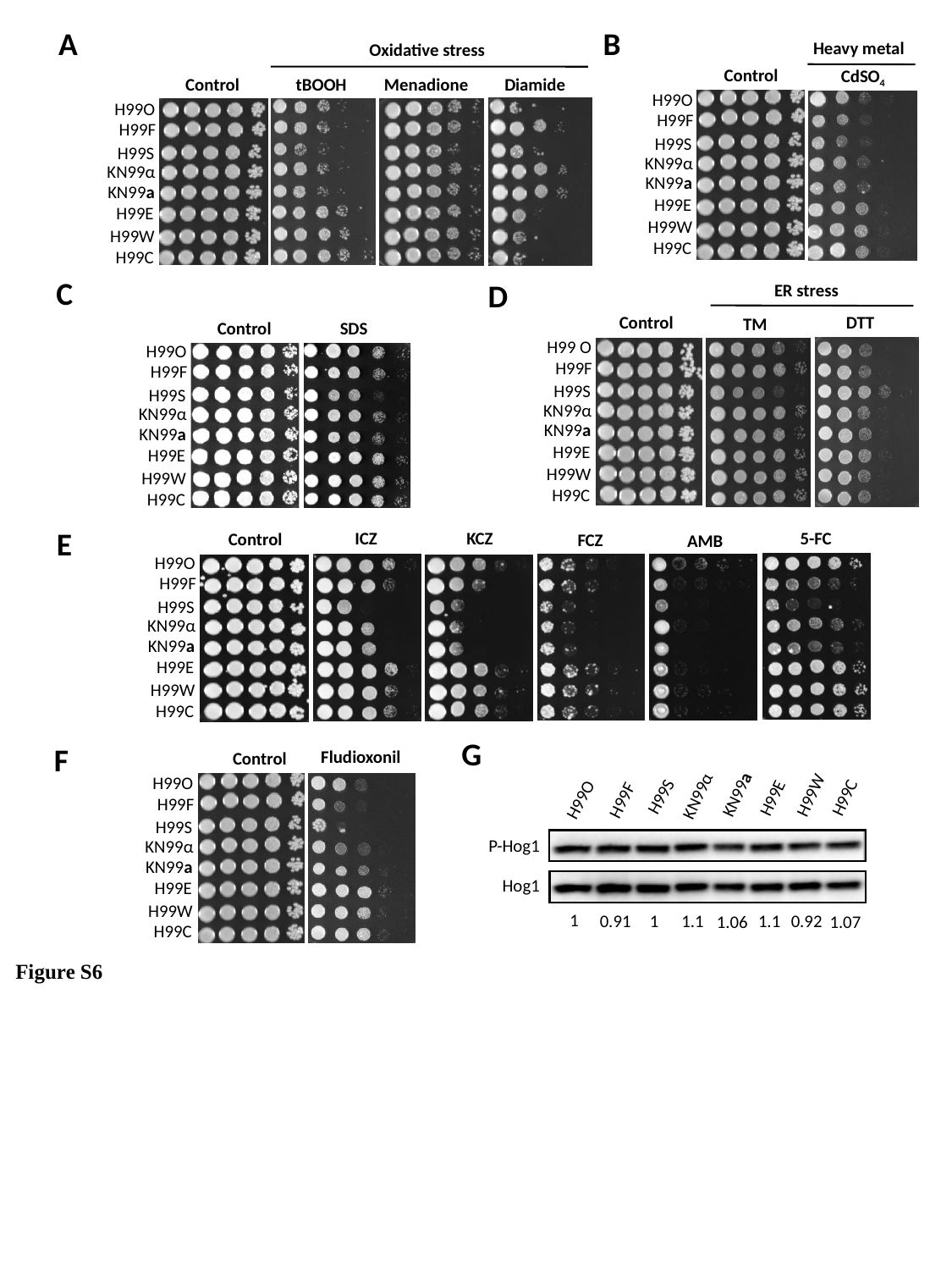

A
B
Heavy metal
Oxidative stress
Control
CdSO4
Control
tBOOH
Menadione
Diamide
H99O
H99O
H99F
H99F
H99S
H99S
KN99α
KN99α
KN99a
KN99a
H99E
H99E
H99W
H99W
H99C
H99C
C
D
ER stress
Control
DTT
TM
Control
SDS
H99 O
H99O
 H99F
 H99F
H99S
H99S
KN99α
KN99α
KN99a
KN99a
H99E
H99E
H99W
H99W
H99C
H99C
E
ICZ
5-FC
KCZ
Control
FCZ
AMB
H99O
 H99F
H99S
KN99α
KN99a
H99E
H99W
H99C
G
F
Fludioxonil
Control
H99O
H99E
H99F
H99W
KN99α
H99O
KN99a
H99S
H99C
H99F
H99S
P-Hog1
KN99α
KN99a
Hog1
H99E
H99W
1
1.1
0.91
1
1.1
0.92
1.06
1.07
H99C
Figure S6
