# Supplementary material for: Analysis of the Genome and Transcriptome of Cryptococcus neoformans var. grubii Reveals Complex RNA Expression and Microevolution Leading to Virulence Attenuation
Source: PLoS Genet. 2014 Apr 17;10(4):e1004261. doi: 10.1371/journal.pgen.1004261 (PMC3990503; doi:10.1371/journal.pgen.1004261)
Supplement: Table S1 — List of the modifications of the C. neoformans genome annotation. (DOC) [file pgen.1004261.s011.doc]

Table S1. List of the modifications of the *C. neoformans* genome annotation.

| Modification  type | Specific  change | Gene numbers | Running count | Change description |
| --- | --- | --- | --- | --- |
| add |  | 47 | 47 | A gene has been added to a region that previously had none |
| change | extended | 1 | 48 | CDS change: splice agreement, new model is longer; has upstream start and downstream stop |
| change | extended - new start | 452 | 500 | CDS change: splice agreement and same stop; new model is longer, has upstream start |
| change | other Change | 1094 | 1594 | CDS change: other model change not covered by another category |
| change | shift | 2 | 1596 | CDS change: splice agreement; new model has upstream start and stop OR downstream start and stop |
| change | splice site changed | 1247 | 2843 | CDS change: same start and same stop; internally, a splice site moved |
| change | truncated- new start | 17 | 2860 | CDS change: splice agreement and same stop; new model is shorter, has downstream start |
| change | UTR changed | 3834 | 6694 | Splice agreement and same start and same stop, but differ in UTR |
| delete |  | 34 |  | Gene no longer present at this locus |
| merge |  | 48 | 6718 | Multiple old genes have been merged into one |
| split |  | 12 | 6730 | A single old gene has been split into multiple new genes |
| unchange |  | 232 | 6962 | Primary transcript is identical |
| totals |  | 6962 |  |  |
