# Supplementary material for: Analysis of the Genome and Transcriptome of Cryptococcus neoformans var. grubii Reveals Complex RNA Expression and Microevolution Leading to Virulence Attenuation
Source: PLoS Genet. 2014 Apr 17;10(4):e1004261. doi: 10.1371/journal.pgen.1004261 (PMC3990503; doi:10.1371/journal.pgen.1004261)
Supplement: Table S4 — Genes expressed without an intron in C. neoformans var. grubii. (DOC) [file pgen.1004261.s014.doc]

**Table S4:** Genes expressed without an intron in *C. neoformans* var. *grubii.*

| locus | Protein aa | Putative function |
| --- | --- | --- |
| CNAG_06099 | 222 | hyp prot crypto specific |
| CNAG_04246 | 359 | hyp prot crypto specific |
| CNAG_03740 | 335 | hyp prot crypto specific |
| CNAG_01645 | 403 | hyp prot basidio specific |
| CNAG_04309 | 293 | hyp prot basidio specific |
| CNAG_05916 | 331 | hyp prot crypto specific |
| CNAG_07471 | 191 | hyp prot crypto specific |
| CNAG_06028 | 413 | hyp prot basidio specific |
| CNAG_02425 | 290 | nicotinamide/nicotinate mononucleotide adenylyltransferase domain |
| CNAG_01906 | 388 | mitochondrial inheritance component mdm12 |
| CNAG_02910 | 428 | hyp prot crypto specific |
| CNAG_03930 | 296 | hyp prot basidio specific |
| CNAG_05476 | 222 | hyp prot basidio specific |
| CNAG_07776* | 511 | pseudouridylate synthase |
| CNAG_06771 | 431 | hyp prot crypto specific |
| CNAG_04901 | 236 | hyp prot crypto specific |
| CNAG_01603 | 331 | hyp prot crypto specific |
| CNAG_05547 | 507 | hyp prot basidio specific |
| CNAG_04294 | 182 | hyp prot crypto specific |
| CNAG_04094 | 179 | hyp prot cupin 2 |
| CNAG_02495 | 421 | hyp prot concerved |
| CNAG_01166 | 327 | UreD-domain-containing protein |
| CNAG_01220 | 215 | mitochondrial FAD-linked sulfhydryl oxidase ERV1 |
| CNAG_03083 | 188 | hyp prot cupin 2 |
| CNAG_03293 | 214 | processing of 20S pre-rRNA-related protein |
| CNAG_03961 | 252 | hyp prot Ribosomal protein L10 family |
| CNAG_04675 | 234 | hyp prot crypto specific |
| CNAG_03185 | 213 | NADPH-dependent FMN reductase domain |
| CNAG_04045 | 225 | hyp prot basidio specific |
| CNAG_01843 | 369 | elongation factor ts |
| CNAG_03082* | 189 | hyp prot cupin 2 |
| CNAG_02933 | 350 | quinone oxidoreductase |
| CNAG_05507 | 165 | mitochondrial ribosomal protein mrps8 |
| CNAG_01789 | 170 | ribosomal protein L31 |
| CNAG_06602 | 252 | cysteine-type peptidase |

* These genes possess a clear antisense transcript.
