# Supplementary material for: Analysis of the Genome and Transcriptome of Cryptococcus neoformans var. grubii Reveals Complex RNA Expression and Microevolution Leading to Virulence Attenuation
Source: PLoS Genet. 2014 Apr 17;10(4):e1004261. doi: 10.1371/journal.pgen.1004261 (PMC3990503; doi:10.1371/journal.pgen.1004261)
Supplement: Table S6 — Coordinates of the centromeric regions in C. neoformans H99. (DOC) [file pgen.1004261.s016.doc]

**Table S6. Coordinates of centromeric regions in *Cryptococcus neoformans* H99.**

| Chromosome no. | Coordinate start  (ORF no.) | Coordinate end  (ORF no.) | Size (bp) | AT% |
| --- | --- | --- | --- | --- |
| 1 | 970169 (CNAG_00378) | 1006713 (CNAG_00383) | 36545 | 52.5% |
| 2 | 835384 (CNAG_03796) | 892713 (CNAG_03805) | 57330 | 51.3% |
| 3 | 1378288 (CNAG_07972) | 1409632 (CNAG_06879) | 31345 | 53.3% |
| 4 | 708804 (CNAG_05202) | 750080 (CNAG_05215) | 41276 | 51.3% |
| 5 | 1559983 (CNAG_00960) | 1580220 (CNAG_00941) | 20238 | 52% |
| 6 | 780649 (CNAG_02254) | 821762 (CNAG_02240) | 41114 | 52.4% |
| 7 | 525714 (CNAG_06699) | 570802 (CNAG_05671) | 45089 | 51.9% |
| 8 | 451162 (CNAG_03251) | 505241 (CNAG_03258) | 54080 | 50.7% |
| 9 | 801830 (CNAG_04417) | 840435 (CNAG_07769) | 38606 | 53.5% |
| 10 | 199434 (CNAG_04857) | 230149 (CNAG_04855) | 30716 | 52.8% |
| 11 | 868824 (CNAG_01780) | 933658 (CNAG_01788) | 64835 | 52.6% |
| 12 | 139633 (CNAG_06036) | 170629 (CNAG_06048) | 30997 | 52.3% |
| 13 | 579772 (CNAG_06475) | 632167 (CNAG_06482) | 52396 | 51.5% |
| 14 | 441845 (CNAG_05486) | 477986 (CNAG_05496) | 36141 | 51.3% |
