# Supplementary material for: Analysis of the Genome and Transcriptome of Cryptococcus neoformans var. grubii Reveals Complex RNA Expression and Microevolution Leading to Virulence Attenuation
Source: PLoS Genet. 2014 Apr 17;10(4):e1004261. doi: 10.1371/journal.pgen.1004261 (PMC3990503; doi:10.1371/journal.pgen.1004261)
Supplement: Table S7 — Positions of the replication origin in C. neoformans. (DOC) [file pgen.1004261.s017.doc]

**Table S7: Positions of the replication origin in *C. neoformans.***

| **Origin^1^** | ***C. neoformans* var. *neoformans* JEC21 genome location** | ***C. neoformans* var. *grubii* H99 genome location** | **Initiation zone** |
| --- | --- | --- | --- |
| rDNA ORI | Chromosome 2  281,586-284,562 | Chromosome 2  278,336-381,505 |  |
| CnORI1.127 | Chromosome 1  1,276,347-1,279,903 | No clear homology—see note below | Yes; complete bubble arc in overlapping PvuII fragment to the left |
| CnORI1.168 | Chromosome 1  1,682,502-1,690,243 | Chromosome 1  1,663,480-1,670,393  1,662,381-1,662,502  1,662,561-1,662,351,  1,662,718-1,662,926  1,662,275-1,663,351 | Yes; complete bubble arc in overlapping ClaI-SacII fragment to the right |
| CnORI1.228 | Chromosome 1  2,283,347-2,288,106 | Chromosome 1  2,262,389  2,266,884 | Maybe |
| CnORI3.017 | Chromosome 3  171,730-177,313 | Chromosome 3  175,317-180,919 | Not examined |
| CnORI4.113 | Chromosome 4  1,130,539-1,136,359 | Chromosome 5  689,980-684,183 | Confined to RI fragment |
| CnORI5.097 | Chromosome 5  974,955-979549—RI  972,555-979549-bubble | Chromosome 6  941,112-945,666  940,237-945,666  939,213-939,524  939,483-940,113 | Yes; complete bubble arc in overlapping HindIII fragment |
| CnORI7.010 | Chromosome 7  101,961-107,491 | Chromosome 8  1,285,877-1,280,354 |  |

^1^The origins identified have been named systematically in a way that describes their genomic positions. ‘CnORI’ specifies a Cryptococcus origin, and the numbers following indicate the chromosome number, followed by a decimal point and three digits that indicate the position on the chromosome to the nearest 10 kb.

**Note about CnORI1.127:** This origin is within a 7,987-bp region extending from coordinate 1,272,161 to 1,280,147 that is bounded by sequences with high homology to transposon T1 that Joe Heitman’s lab has reported inserting into the FRR gene. The right side matches with nucleotides 1729-1753 of the XW238 element, and the left side is bound by an inverted segment containing nucleotides 2598-2753 of the transposon.  The coordinates are based on the sequence of the mobile element, not including the FRR gene.  The middle of the region contains another segment that stretches from nucleotides 1118-1727 of the transposon.

The other sequences included in the region match some annotated proteins in the JEC21 genome.  There are some poor matches to H99 chromosomes 4 and 14.  However, sequences on both sides of this insertion show robust matches to H99 chromosome 1.
