# Supplementary material for: Analysis of the Genome and Transcriptome of Cryptococcus neoformans var. grubii Reveals Complex RNA Expression and Microevolution Leading to Virulence Attenuation
Source: PLoS Genet. 2014 Apr 17;10(4):e1004261. doi: 10.1371/journal.pgen.1004261 (PMC3990503; doi:10.1371/journal.pgen.1004261)
Supplement: Table S8 — Cryptococcus orthologs of DNA replication initiation proteins. (DOC) [file pgen.1004261.s018.doc]

**Table S8. *Cryptococcus* orthologs of DNA replication initiation proteins.**

| **Protein** | **H99** | **JEC21** | **Annotation in H99 database** |
| --- | --- | --- | --- |
| **Replicative helicase orthologs** | | | |
| MCM2 | CNAG_03341 | CNG02380 | DNA replication licensing factor cdc19 |
| MCM3 | CNAG_00099 | CNA00900 | MCM ATP-dependent helicase |
| MCM4 | CNAG_06182 | CNM01820 | MCM DNA unwinding related protein |
| MCM5 | CNAG_04052 | CNB05360 | MCM ATP-dependent helicase |
| MCM6 | CNAG_03962 | CNB04510 | MCM DNA unwinding related protein |
| MCM7 | CNAG_05825 | CNF01550 | MCM ATP-dependent helicase |
|  |  |  |  |
| **ORC protein orthologs** | | | |
| ORC1 | CNAG_02195 | CNE03440 | replication control protein 1 |
| CDC6 | CNAG_04703 | CNJ01340 | DNA clamp loader |
| SIR3 | CNAG_04696 | CNJ01260 | DNA clamp loader |
|  |  |  |  |
| ORC2 | CNAG_07162 | CNK01310 | DNA replication origin binding protein |
| ORC3 | CNAG_00362 | CNA03410 | hypothetical protein |
| ORC4 | CNAG_06183 | CNM01830 | origin recognition complex subunit 4 |
| ORC5 | CNAG_00310 | CNA02930 | hypothetical protein |
| ORC6 | No hit | No hit |  |
|  |  |  |  |
| **Other initiation proteins** | | | |
| CDC6 | CNAG_04703 | CNJ01340 | DNA clamp loader, notes homology to C-terminus of Cdc6 |
| CDT1 (TAH11) | CNAG_02413 | CNE01430 |  |
