# Supplementary material for: Analysis of the Genome and Transcriptome of Cryptococcus neoformans var. grubii Reveals Complex RNA Expression and Microevolution Leading to Virulence Attenuation
Source: PLoS Genet. 2014 Apr 17;10(4):e1004261. doi: 10.1371/journal.pgen.1004261 (PMC3990503; doi:10.1371/journal.pgen.1004261)
Supplement: Table S9 — SNPs and indels identified in H99 series. (DOC) [file pgen.1004261.s019.doc]

Table S9. SNPs and indels identified in the H99 derived series.

| **ID** | **Chromosome; Coordinates** | **H99 reference sequence** | **Mutation** | **Details** |
| --- | --- | --- | --- | --- |
| **SNP1** | 1; 825,487 | A | G | CNAG_00321, intron |
| **SNP2** | 2; 1,568,847 | C | T | CNAG_04078, non-synonymous, F3S |
| **SNP3** | 5; 688,951 | A | T | miscRNA CNAG_12447 |
| **SNP4** | 5; 1,286,519 | G | A | CNAG_01069, synonymous; CNAG_07441, 3’ UTR |
| **SNP5** | 10; 174,524 | C | T | CNAG_04864, synonymous |
| **SNP6** | 10; 344,002 | C | T | Intergenic |
| **SNP7** | 11; 1,457,622 | G | A | CNAG_01987, intron |
| **SNP8** | 11; 223,290 | G | T | CNAG_01536, synonymous |
| **SNP9** | 11; 241,670 | G | A | CNAG_01541, synonymous |
| **SNP10** | 11; 375,655 | C | T | CNAG_01594, non-synonymous, T64I |
| **SNP11** | 13; 529,185 | C | G | CNAG_06456, non-synonymous, Q298E |
| **Indel1** | 1; 12,714 | G | GA | Intergenic |
| **Indel2** | 2; 99,355 | C | CCTGGCCG | CNAG_06765, frameshift at residue 145 of 1195 |
| **Indel3** | 2; 208,187 | CGCAT | C | CNAG_06730, 3’ UTR |
| **Indel4** | 2; 1,059,379 | ACCTACATTC  GTTACTG | A | CNAG_03873, 5’ UTR |
| **Indel5** | 6; 538,398 | A | ACTC | CNAG_07634, insertion of E1053 of 1458 |
| **Indel6** | 7; 150,814 | C | CT | Intergenic |
| **Indel7** | 7; 1,361,217 | G | GA | CNAG_05970, 3' UTR |
| **Indel8** | 8; 1,289,136 | C | CG | Intergenic |
| **Indel9** | 9; 5,478 | C | CA | Intergenic |
| **Indel10** | 11; 550,343 | CT | C | CNAG_07595, frameshift at residue 605 of 770 |
| **Indel11** | 14; 497,587 | TA | T | CNAG_05505, intron |
