# Supplementary material for: Analysis of the Genome and Transcriptome of Cryptococcus neoformans var. grubii Reveals Complex RNA Expression and Microevolution Leading to Virulence Attenuation
Source: PLoS Genet. 2014 Apr 17;10(4):e1004261. doi: 10.1371/journal.pgen.1004261 (PMC3990503; doi:10.1371/journal.pgen.1004261)
Supplement: Table S10 — Sequencing read statistics. (DOC) [file pgen.1004261.s020.doc]

| Table S10 Sequencing Read Statistics | | | |  |  |
| --- | --- | --- | --- | --- | --- |
| library | Sequencing center | Mean insert length | reads | Total bases | coverage |
| Plasmid | Broad (WIBR*) | 5008 | 223889 | 189633983 | 7.90 |
| Plasmid | Duke | 2095 | 95052 | 63589788 | 2.65 |
| Plasmid | Broad (WIBR*) | 11995 | 60176 | 41401088 | 1.73 |
| Fosmid | Broad (WIBR*) | 40953 | 28639 | 21794279 | 0.91 |
| BAC | Broad (WIBR*) | 93560 | 6602 | 3908384 | 0.16 |
| BAC | Duke | 104793 | 4705 | 2950035 | 0.12 |

*WIBR: Whitehead Institute for Biomedical Research
