# Supplementary material for: Analysis of the Genome and Transcriptome of Cryptococcus neoformans var. grubii Reveals Complex RNA Expression and Microevolution Leading to Virulence Attenuation
Source: PLoS Genet. 2014 Apr 17;10(4):e1004261. doi: 10.1371/journal.pgen.1004261 (PMC3990503; doi:10.1371/journal.pgen.1004261)
Supplement: Table S12 — Primers used in this study. (DOC) [file pgen.1004261.s022.doc]

**Table S12. Primers used in this study**

| **UQ ID** | **Function** | **Sequence** |
| --- | --- | --- |
| UQ198 | Chr 9L probe forward | CTGTGCCGTCCTCTCCCATCC |
| UQ199 | Chr 9L probe reverse | TTGCCCATCCATGATCCACTT |
| UQ202 | Chr 9R probe forward | GTTATTGCGAATACCATAGGG |
| UQ203 | Chr 9R probe reverse | AGAGGGCTTCGCATAGGAATC |
| UQ618 | Generic telomere reverse | CCTAACCCCCTAACCCCCTAA |
| UQ1258 | Chr 9 telomere search primer 1 | AGAAGTGGTACAGCGGTGAAA |
| UQ1259 | Chr 9 telomere search primer 2 | ATCCTGGGCTGATTTCCGATT |
| UQ1260 | Chr 9 telomere search primer 3 | ACGGACTATACGACGGTAAAG |
| UQ1261 | Chr 9 telomere search primer 4 | GGAAGGAGGAGACTGGGCACA |
| UQ1423 | Chr 9 breakpoint crossing reverse | CGGCCAGAGAAAGAGTAGCTG |
| UQ2265 | Indel 10 forward | CCCAACCTTACACTATCCATC |
| UQ2266 | Indel 10 reverse | CACCACCATCAACTATCTCCA |
| UQ2267 | Indel 11 forward | CACGTCGTCCTTACTAATATC |
| UQ2268 | Indel 11 reverse | TTCCATACCGCTTCCATAGTC |
| UQ2271 | Indel 8 forward | CAAAGACTGGGTATCATCAT |
| UQ2272 | Indel 8 reverse | TTTACTCCTCTTGCTCAACT |
| UQ2273 | Indel 1 forward | GTCTTTCGTCCACGGTTGTT |
| UQ2274 | Indel 1 reverse | ACAAGCCTTGGCTGTGACTT |
| UQ2275 | Indel 2 forward | ACAATATGGAATGGCAAGAC |
| UQ2276 | Indel 2 reverse | TGAGATGGTTGGAGTTGATG |
| UQ2277 | Indel 3 forward | TTGCGTGGCTAGTGGTTATT |
| UQ2278 | Indel 3 reverse | AGGAAAAGGGAGTATGAGGAG |
| UQ2279 | Indel 4 forward | TTCCCGCCATCCATCATTTT |
| UQ2280 | Indel 4 reverse | ATCAGCCCTCCTCTTCTACA |
| UQ2390 | Indel 5 forward | ATACTGAAAACTTACTGTGCC |
| UQ2282 | Indel 5 reverse | GAGCAGAGATTCAACGGGAT |
| UQ2283 | Indel 6 forward | GATCTTTCTTCCCCCTTTTT |
| UQ2284 | Indel 6 reverse | TTACGTGTCCGTTTTTCCCA |
| UQ2285 | Indel 9 forward | GGCAAAATCACGAAACAAGAC |
| UQ2286 | Indel 9 reverse | ATGACGCTAGATGGAATGAGG |
| UQ2391 | Indel 7 reverse | AAAGTACGTAATGCACTGCCT |
| UQ2392 | Indel 7 forward | TTTCATCCGCTACTGCATTTG |
| UQ2413 | *LMP1*Δ 5' flank forward | TCTTGCCGATAATCACTTCTG |
| UQ2414 | *LMP1*Δ 5' flank overlap reverse | CGCTCTCCAGCTCACATCCTCGCAGCAGTATGGCTGTGATTGTG |
| UQ2415 | *LMP1*Δ 3' flank overlap forward | CTACATCTCTTCCGTGTTAATACAGATAAACCATGCCAGTGACATG |
| UQ2416 | *LMP1*Δ 3' flank reverse | TATGGACAACAGTCTGGACCC |
| UQ2417 | *LMP1*Δ NEO forward | CACAATCACAGCCATACTGCTGCGAGGATGTGAGCTGGAGAGCG |
| UQ2418 | *LMP1*Δ NEO reverse | CATGTCACTGGCATGGTTTATCTGTATTAACACGGAAGAGATGTAG |
| UQ2423 | *LMP1*Δ Sequencing primer 1 | GGCGTACACACCAATACTTTA |
| UQ2424 | *LMP1*Δ Sequencing primer 2 | CAAGGTGTTCAAATAGACAAT |
| UQ2425 | *LMP1*Δ Sequencing primer 3 | ACATCTCTGACTCAGCGTAGA |
| UQ2426 | *LMP1*Δ Sequencing primer 4 | CTACAACCATAGGAATACCTG |
| UQ2427 | *LMP1*Δ Sequencing primer 5 | AATGAACTACAGTGCTCAGAG |
| UQ2428 | *LMP1*Δ Sequencing primer 6 | GGGCTTAATGTTACTAATGAT |
| UQ2429 | *LMP1*Δ Sequencing primer 7 | GATACACGACAACAATCATCA |
| UQ2430 | *LMP1*Δ Sequencing primer 8 | TATCCGCAATCTATATATGAT |
| UQ2431 | *LMP1*Δ 5’ diagnostic primer | CTTCAAAGGGCAGAATAACAA |
| UQ2434 | *LMP1*Δ 3' diagnostic primer | GTTAATAAGTTGGGCAGCAGC |
| CEN14 FP | CENP-A ChIP primer | TGCTCTTCGTTGTAGTGGTC |
| CEN14 RP | CENP-A ChIP primer | GGTAGTTAACGAGGCCGAG |
| LEU2 FP | CENP-A ChIP primer | TCAGCCGTTCTCAAGGATGAG |
| LEU2 RP | CENP-A ChIP primer | ACTTGAGATCAAGCTTGAGATCAG |
| GI008 | LEFT_CEN14 HR region - forward | CCGCTCGAGGGCTTCACTCATCACCGGAA |
| GI009 | LEFT_CEN14 HR region - reverse | CCGCTCGAGTCACCCTCAAAGCCTGTTTCA |
| GI010 | RIGHT_CEN14 HR region - forward | CCGCTCGAGCGATAAATATTTCTGACTC |
| GI005 | RIGHT_CEN14 HR region - reverse | GTCTCCATAAGTTGGGGAAT |
| GI003 | NAT + telomeric sequence | [CCCCCTAA(x11)]GAAGAGATGTAGAAACGAG |
| GI013 | NAT LEFT_CEN14 KO (correct orientation) | TTCCGGTGATGAGTGAAGCCCTCGAGCGGGGGAGGACTCACATAAGC |
| GI014 | NAT LEFT_CEN14 KO (opposite orientation) | TGAAACAGGCTTTGAGGGTGACTCGAGCGGGGGAGGACTCACATAAGC |
| GI015 | NAT RIGHT_CEN14 KO (correct orientation) | CTCCTCGAGATTCCCCAACTTATGGAGACGGGAGGACTCACATAAGC |
| GI016 | NAT RIGHT_CEN14 KO (opposite orientation) | GAGTCAGAAATATTTATCGCTCGAGCGGGGGAGGACTCACATAAGC |
| ai270 | KO screening | ACTTCTCGCAAAGTGAATCC |
| GI033 | KO screening LEFT_CEN14 - reverse | CAGCAATAGCAAGTTCAAGA |
| GI034 | KO screening RIGHT_CEN14 - forward | CCAATGATTGTTAGTTTGCT |
