# Supplementary material for: Analysis of the Genome and Transcriptome of Cryptococcus neoformans var. grubii Reveals Complex RNA Expression and Microevolution Leading to Virulence Attenuation
Source: PLoS Genet. 2014 Apr 17;10(4):e1004261. doi: 10.1371/journal.pgen.1004261 (PMC3990503; doi:10.1371/journal.pgen.1004261)

**Text S1**

**History of the H99 strain**

H99 is a clinical strain of the A serotype of *C. neoformans* var. *grubii*. It was first isolated directly from the cerebrospinal fluid (CSF) of a 28-year-old Caucasian male who had been treated for Hodgkin’s disease. The patient lived in the Piedmont area of North Carolina and had received corticosteroids. The initial exam and CSF parameters are included in the consult note of February 14, 1978 (see below). To summarize the human pathological features of strain H99, it produced a high enough yeast burden in human disease to have a positive India ink examination. Furthermore, it caused increased intracranial pressure and hypoglycorrhachia. The CSF cellular reaction was modest and primarily consisted of lymphocytes. The disease production of this yeast in the host was manifested by headaches and specific neurological findings. The patient was treated for cryptococcal meningoencephalitis for six weeks with amphotericin B at 0.3 mg/kg/d and flucytosine 150 mg/kg/d. After one year of follow-up, the patient appeared to be cured. Since then, strain H99 has been a pivotal strain in the research into mechanisms of fungal pathogenesis. It is valuable because: (1) it represents the major clinical serotype with a detailed history and its immunological impact has been interrogated; (2) it has the demonstrated ability to produce human disease; (3) its pathogenicity is well-described in multiple animal models; (4) it is amenable to molecular techniques such as gene transfer, site-directed mutagenesis, and the production of mutant libraries; (5) it is useful for performing genetic crosses and developing congenic strains and; (6) it has become the type culture for the A serotype and var. *grubii*. In addition, it has been properly stored as a reference isolate and disseminated throughout the research community. With this strong genetic pedigree combined with robust animal models and well-described production of clinical disease, the last two decades have witnessed an explosion of studies on the virulence composite of H99 and the host immunity to this pathogenic encapsulated yeast strain.

**Consult note of February 14, 1978**


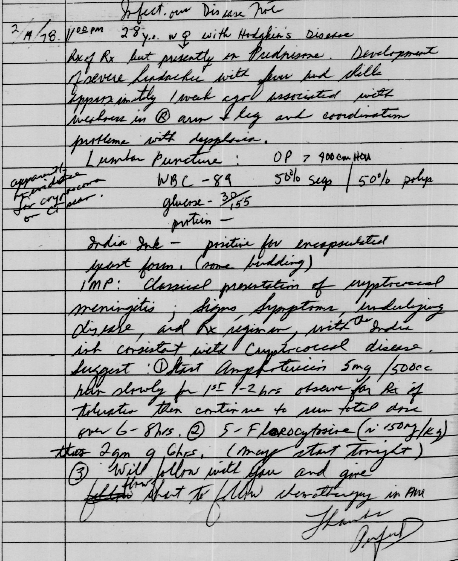

Supplement: Text S1 — History of the H99 strain and consult note of February 14, 1978. (DOC) [file pgen.1004261.s023.doc]
